# Immunomodulatory Effect of *Withania somnifera* (Ashwagandha) Extract—A Randomized, Double-Blind, Placebo Controlled Trial with an Open Label Extension on Healthy Participants

**DOI:** 10.3390/jcm10163644

**Published:** 2021-08-18

**Authors:** Ajit Tharakan, Himanshu Shukla, Irin Rosanna Benny, Matthan Tharakan, Lekha George, Santhosh Koshy

**Affiliations:** 1 Department of Cardiothoracic Surgery, Oklahoma Heart Institute, University of Oklahoma College of Medicine, Tulsa, OK 73104, USA; 2 Department of General Medicine, PGH Hospital, Uttam Nagar, New Delhi 110059, India; himanshu502000@yahoo.com; 3 Department of Pathology, Christian Medical College, Vellore 632004, India; irinrosannabenny@gmail.com; 4 College of Engineering and Natural Sciences, Tulsa University, Tulsa, OK 74107, USA; matthantharakan@yahoo.com; 5 Department of Medicine, Texas Tech Health Science University, Lubbock, TX 79430, USA; Lekha.George@ttuhsc.edu (L.G.); Santhosh.Koshy@ttuhsc.edu (S.K.)

**Keywords:** *Withania somnifera*, withanolide glycosides, immunomodulation, immunity, Th1–Th2 pathway

## Abstract

The immunomodulatory effect of *Withania somnifera* (WS) extract was tested in healthy adults. In this randomized placebo-controlled double-blinded study, subjects were allocated either 60 mg WS extract or placebo. It consists of a blinded 30-day period and an open-label extension study of another 30 days with crossover of only placebo to test. After the 30-day blinded study period, the WS test group reported significant increase (*p* < 0.05) in Ig’s (IgA, IgM, IgG, IgG2, IgG3 and IgG4), Cytokines (IFN-γ, IL4), TBNK (CD45+, CD3+, CD4+, CD8+, CD19+, NK cells) whereas in the placebo group TBNK cells showed significant decrease (*p* < 0.05) and Ig’s and cytokines showed no change (*p* > 0.05). In the extension period on day 60, the subjects on placebo who were crossed over to the WS test group showed significant increase (*p* < 0.05) in Ig’s, cytokines and TBNK cells and the subjects who continued on the WS group showed a further significant improvement (*p* < 0.05) in Ig’s, cytokines and TBNK cells. There were no adverse events reported in the study. WS extract significantly improved the immune profile of healthy subjects by modulating the innate and adaptive immune systems. Boosting the immune system of people at risk of infection and during widespread infections can be targeted with WS extract.

## 1. Introduction

The mammalian immune system consists of three levels of defense: the physical barrier, the innate immune system, and the adaptive immune system [1]. Immunomodulators can be broadly classified into immunostimulants and immunosuppressants. Immunostimulants enhance the defense mechanisms and are used to prevent infections and cancer while immunosuppressants suppress the immune system and prevent autoimmune response and graft rejections. Immunomodulators of plant origin are a promising field of research and many potential candidates have been identified. Among them, Ashwagandha (*Withania somnifera* or WS) which is mentioned in the Rasayana group of medications in the Ayurveda tradition of India has been extensively investigated as an immunomodulatory agent. The components in WS mainly withanolide glycosides exert their immunomodulatory action by mobilizing and activating macrophages and induce proliferation in murine splenocytes [2]. This effect has been suggested to be the idea of its use as a Rasayana in Ayurveda.

However, the effect of WS on the immunoregulatory cellular response has not been tested yet in humans. Hence, the extract of WS can be a future drugs candidate that might address the anti-viral or antimicrobial infections together with the induced immune response to prevent or control the infections. The involvement of immune-regulatory cells induced by WS extracts might have several functions, such as regulating antigen presentation and control of immunosuppressive microenvironment along with a physiological cytokine milieu for an effector T cell function [3].

The current study was an effort to evaluate the immune-modulating effects of WS extract comparing against placebo, in middle to the aged healthy population exposed to environmental influences of seasonal change in a double-blind cross-over design. From the literature available, this appears to be the first human study with WS extract on immunity and the results of the study has significant relevance considering the recent prevalence of viral and microbial infections worldwide.

## 2. Materials and Methods

### 2.1. Study Design

This placebo-controlled, double-blinded, parallel-arm single-center pilot study was conducted at PGH Hospital, Delhi. The study had a treatment period of 30 days with an open-label extension period of another 30 days. In the extension period, the participants in the placebo group were crossed over to the test group. In the test group, crossover to placebo was not done after 30 days because of anticipated carry-over effects of WS and continued with the test group. The primary objective of the study was to determine the immunomodulatory effect of WS at the end of the 30-day study period. The study was prospectively registered (CTRI/2018/07/014792) and was conducted according to the declaration of Helsinki in agreement with the International Conference on Harmonisation (ICH) guidelines on Good Clinical Practice (GCP). All the subjects participating in the study were required to read, understand, and execute an informed consent form in writing.

### 2.2. Subjects and Inclusion/Exclusion Criteria

The study included 24 adult male and female healthy subjects of 45–72 years of age. The main inclusion criteria were a BMI < 30, generally good health status confirmed by clinical history, physical exam, and routine blood analysis. Subjects with prior history or presence of clinically significant cardiovascular, pulmonary, hepatic, renal, hematological, gastrointestinal, endocrine, immunologic, dermatologic, musculoskeletal, neurological or psychiatric disease or those who had undergone surgery during last one year or received organ transplantation or chronic smokers or alcohol/drug abuse or pregnant/lactating women or having any kind of allergy were excluded from the study. The participant must not have taken any vitamin/mineral/dietary or herbal supplements 1 month before enrolling in the study and agrees to not use any new vitamins, minerals, dietary or herbal supplements until after study completion.

### 2.3. Intervention and Dosing

The test product was an ethanolic powder extract of dried roots and leaves of *Withania somnifera*. The herb was visually identified by a qualified botanist and a voucher specimen was kept with the herbarium ID HERB-ED-22. Dried roots and leaves of WS were powdered and underwent hydroalcoholic extraction (raw material to solvent ratio 1:10) and from the concentrated liquid portion, withanolide glycosides were enriched by partitioning with alcohol and water to obtain WS extract with 35% WG with a herb to extract ratio of 40:1. An HPLC with PDA detector and C18 column using a gradient solvent system of potassium dihydrogen phosphate buffer and acetonitrile, wavelength 227 nm was used to quantify WG using withanoside IV as reference standard [4].

Subjects were randomized to receive a daily dose of either one 180 mg capsule containing 60 mg of WS (test product, Shoden (Arjuna Natural Private Ltd., Kerala, India) with 21 mg withanolide glycosides or a matching placebo containing roasted rice powder for 30 days. After the end of the 30-day study period, the subjects in the placebo group were crossed over to the test group, those in the test group continued to take the test product.

### 2.4. Randomization, Blinding and Unblinding

The randomization sequence and master list were generated using a balanced randomization method, GraphPad web version 2017 by an independent statistician. The randomization list was then allocated and concealed using an alphanumeric code and kept with the pharmacist under restricted access. The intervention products were dispensed by the pharmacist. The allocation ratio was 1:1 with 12 subjects in each of the test and the placebo groups.

The study was a double-blinded placebo-controlled study of 30 days with an open-label extension period of another 30 days. The investigator and the subjects were blinded towards the identity of the intervention for the first 30-day study period. The test and placebo capsules were opaque and of similar color, size, and shape. The interventions were packed and sealed in opaque bottles of similar size and label and could only be distinguished by their allocation concealment codes. In the extension period, since only the placebo group was crossed over to the test group, blinding could not be applied. Sealed opaque envelopes containing individual treatment details were used as the unblinding method. In the case of a medical emergency that necessitated knowledge of the treatment given, the individual envelope is opened and documented.

### 2.5. Outcome Assessments

The blood samples for TBNK, cytokines, and immunoglobulins were collected (fasting morning samples) on the day of randomization, on the 30th day (±2 days) of study completion, and on the 30th day (±2 days) of extension completion. The blood samples for biochemical and safety analysis were collected at screening, on the 30th day (±2 days) of study completion and the 30th day of extension completion.

#### 2.5.1. Immunoglobulin Assay (IgG, IgM, IgA)

The BD CBA Human Immunoglobulin Master Buffer Kit (Cat. No: 558683) and BD CBA Flex Set Assays is a bead-based immunoassay capable of measuring human total IgG (G, G2, G3, G4), IgM and IgA in serum samples using BD FACSDiva 8.5 software. BD CBA Flex Set kit consists of Human Total IgG Bead C6 (Cat. No: 558679), Human IgG2 Flex Set Bead C5 (Cat. No: 558676), Human IgG3 Flex Set Bead C6 (Cat. No: 558677), Human IgG4 Flex Set Bead C7 (Cat. No: 558678), Human IgM Flex Set Bead C8 (Cat. No: 558680), Human IgA Flex Set Bead C9 (Cat. No: 558681) and Phycoerythrin detection reagent. The gating methodology of immunoglobulins is described in Supplementary File 1-Figure S1.

#### 2.5.2. Cytokines Assay

The BD^TM^ Cytometric Bead Array (CBA) Human Th1/Th2 Cytokine Kit (Cat No:550749) was used to quantitatively measure Th1 (interferon-γ) and Th2 (interleukin-4) in a serum sample using flow cytometry and analyzed using BD FACSDiva 8.5 software. The gating methodology of cytokines is described in Supplementary File 1-Figure S2.

#### 2.5.3. TBNK Assay

BD Multitest^TM^ 6-color TBNK reagent (direct immunofluorescence reagent) with BD Trucount tubes kit (Cat. No: 337166) was used with a BD FACSCanto™ flow cytometer with multicheck control to identify and determine the absolute count of human lymphocytes in whole blood. T lymphocytes are identified as CD3+ cells, subset population T helper cells as CD3+ CD4+, T cytotoxic cells as CD3+ CD8+, B cells as CD3− CD19+, and NK cells as CD3− CD16+ CD56+. The gating methodology of TBNK cells are described in Supplementary File 1-Figure S3.

### 2.6. Biochemical and Safety Analysis

CBC, Lipid profile, LFT, RFT, and FBS were analyzed using Cobas c311. Vitals and Adverse events were assessed as treatment-emergent adverse events (TEAEs) throughout the study. These were measured at screening, day 30 and 60.

### 2.7. Statistical Analysis

Twelve subjects were chosen per each arm based on the recommendation for pilot studies considering feasibility, precision about the mean and variance, and regulatory considerations [5]. All the participants who completed the study were included in the analysis. The blinded allocation codes were unblinded by the independent statistician at the time of analysis. Parametric data in immunoglobulins, cytokines, TBNK, and safety profiles were analyzed using the *t*-test for equal variances and non-parametric data were analyzed using Mann–Whitney analysis. Since subjects with high baseline values might have high post-treatment immune response values but with a relatively small increment or vice versa, the Bland–Altman plot method of analysis was used to compare two measurements of the same variable by plotting the difference against the average. The difference would be the mean estimated bias, the systematic difference between the two time-point data and the standard deviation (SD) of the differences would measure random fluctuations around this. In Bland–Altman plot analysis bias, limits of agreement (LOA), and 95% confidence intervals (CIs) for the bias and LOA are calculated. The mean difference is calculated to know the overall bias between baseline data and day-30 data and also day-30 data and day-60 data for each parameter within each group along with corresponding confidence interval for the mean difference. The bias is considered to be significant if the CI of bias does not include the line of equality. The 95% LOA is also calculated as mean diff ± 1.96 SD. In our study, a (non) significant negative or positive bias means that there is a (non) significant increase or decrease respectively in the variables between baseline visit to day 30 or day 30 to day 60 due to the intervention.

## 3. Results

**Figure 1:**
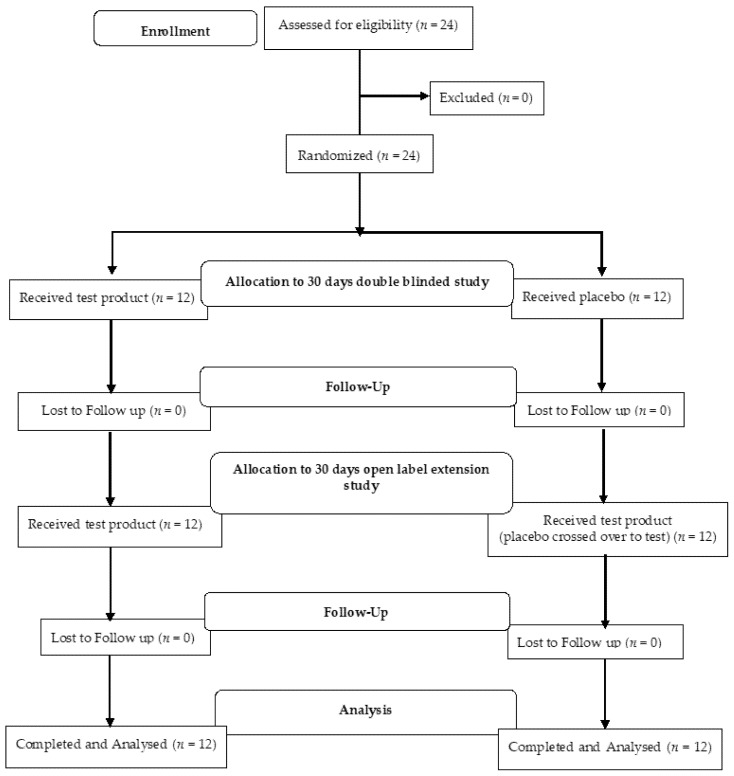
Participants flow diagram.

In the study, all 24 participants screened were randomized and enrolled in the study (Figure 1). The participants were enrolled from mid-August to mid-December and ambient temperature in the region ranged from 34–27 °C to 22–9 °C. The mean age, height, weight, and BMI were similar in both groups at the baseline (Table 1).

### 3.1. Analysis of the Immunoglobulin Level in the Test Group Compared to Placebo

**Figure 2:**
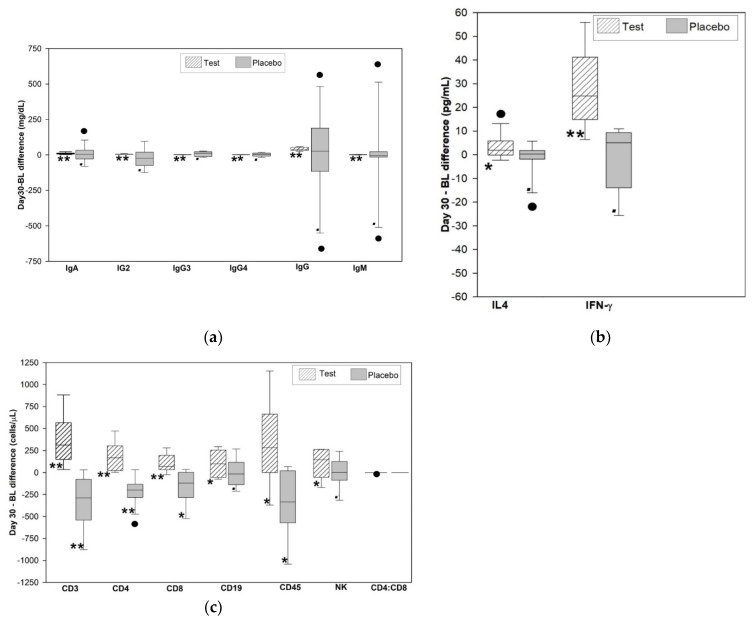
Box plot of the difference between the day 30 and baseline (BL, day0) in participants treated with test (WS) and placebo. (**a**) Comparison of immunoglobulin level (mg/dL) in serum (*n* = 12/group) for the study period (day 0–30) (**b**) Comparison of mean difference (bias) and confidence intervals for the bias of cytokine level (pg/mL) in serum (*n* = 12/group) for the study period (day 0–30). (**c**) Comparison of mean difference (bias) and confidence intervals for the bias of T cells (CD3+ CD4+, CD8+), B cells (CD19+), CD45+, and NK absolute cell counts (cells/µL) in whole blood (*n* = 12/group) for the study period (day 0–30). * Significant at *p* < 0.05. ** Significant at *p* < 0.01. • Not significant (*p* > 0.05).

The within-group analysis of the immunoglobulins (Ig) indicates that only test group showed a significant increase in the level of IgA, IgG2, IgG3, IgG4, IgG, and IgM at the end of the 30-day study (Figure 2a). Levels of all the Ig in the participants crossed over to the test group in the extension study showed a significant increase. The participants who continued to take the test intervention for the total period of 60 days had a significant increase in all their immunoglobulin levels (Table 2). The immunoglobulins were similar between the groups at baseline and the end of 30 days.

**Figure 3:**
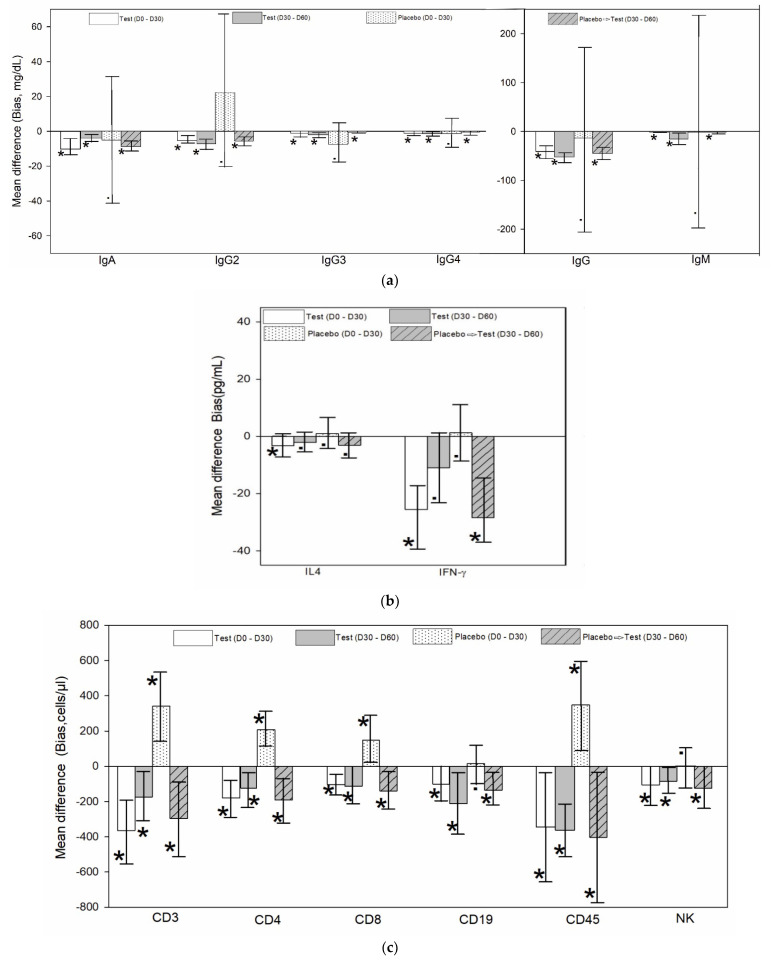
Representative figure for the Bland–Altman analysis data in participants treated with test (WS) and placebo. (**a**) Comparison of mean difference (bias) and confidence intervals for the bias of immunoglobulin level (mg/dL) in serum (*n* = 12/group) for the study period (day 0–30) and extension period (day 30–60). The plots are represented in two scale ranges for better presentation of data. (**b**) Comparison of mean difference (bias) and confidence intervals for the bias of cytokine level (pg/mL) in serum (*n* = 12/group) for the study period (day 0–30) and extension period (day 30–60). (**c**) Comparison of mean difference (bias) and confidence intervals for the bias of T cells (CD3+, CD4+, CD8+), B cells (CD19+), CD45+, and NK absolute cell counts (cells/µL) in whole blood (*n* = 12/group) for the study period (day 0–30) and extension period (day 30–60). The data show the tip of the column corresponding to the mean difference (bias) and the whiskers showing confidence intervals for the bias. The data are taken from independent Bland–Altman plot generated for each parameter and period. (See Supplementary File 2 for actual Bland–Altman plot and tables for each parameter). Placebo➔ Test indicates placebo group crossed over to the test group at the extension period of the study. * Significant at (*p* < 0.05). • Not significant (*p* > 0.05).

The Bland–Altman analysis indicated a mean difference (bias) between the two time periods (Day 0–30 and day 30–60) in both groups (Figure 3a). This was statistically significant (*p* < 0.05) and clinically relevant for the increase in the level of immunoglobulins in the test group for both periods. The placebo did not have a statistically significant increase at the end of 30 days, but the placebo crossed over to the test intervention had a statistically significant (*p* < 0.05) increase and was clinically relevant. The results of the Bland Altman analysis of immunoglobulins are given in Supplementary File 2.

### 3.2. Analysis of the Cytokines (IFN γ and IL4) Level in the Test Group Compared to Placebo

The within-group analysis of the cytokines indicates that the test group showed significant increase in the level of IFNγ (*p* < 0.001) and IL4 (*p* = 0.047) at the end of the 30-day study (Figure 2b). IFNγ and IL4 levels in the participants crossed over to the test group in the extension study showed a significant increase. The participants who continued to take the test product for the total period of 60 days had a significant increase in their IL4 levels and no significant change in IFNγ. IFNγ and IL4 was similar between the groups at baseline and significantly increased in the test group at the end of 30 days (Table 3).

The Bland Altman analysis indicated a mean difference (bias) between the two time periods (Day 0–30 and day 30–60) in both groups (Figure 3b). This was statistically significant (*p* < 0.05) and clinically relevant for the increase in IL4 and IFNγ in the test group for the first period and not significant for the 2nd period. The placebo did not have a statistically significant increase for IL4 and IFNγ. The placebo crossed over to the test intervention had a statistically significant (*p* < 0.05) increase and clinically relevant for IFNγ but not for IL4. The results of the Bland Altman analysis of cytokines are given in Supplementary File 3.

### 3.3. Analysis of the TBNK Cell Absolute Count in the Test Group Compared to Placebo

The within-group analysis of the absolute counts of lymphocytes in test group indicated a significant increase in the number of CD45+, CD3+, CD4+, CD8+, CD19+, and CD16+/CD56+ cells at the end of the 30-day study. The placebo group showed a significant decrease in CD45+, CD3+, CD4+, and CD8+, and no significant change in the CD19+ and CD16+/56+ cells (see Figure 2c). The CD45+, CD3+, CD4+, CD8+, CD19+ and CD16+/56+ counts in the participants crossed over to the test group in the extension study showed a significant increase. The participants who continued to take the test product for the total period of 60 days also had a significant increase in their CD45+, CD3+, CD4+, CD8+, CD19+ and CD16+/56+ counts. The CD4+:CD8+ ratio was conserved throughout the study with the test group having 1.52 ± 0.11 and the placebo crossover to test having 1.49 ± 0.09 at the end of the extension period. A between the group analysis revealed that CD45+, CD3+, CD4+, and CD8+, CD19+ and CD16+/56+ were similar between the groups at baseline and significantly increased in the test group at the end of 30 days (Table 3).

The Bland–Altman analysis indicated a mean difference (bias) between the two time periods (day 0–30 and day 30–60) in both groups (Figure 3c). This was statistically significant (*p* < 0.05) and clinically relevant for the increase in the level of lymphocytes in the test group for both periods. The placebo had a statistically significant and clinically relevant decrease except for CD19+ and NK cells, but the placebo crossed over to the test intervention had a statistically significant (*p* < 0.05) increase and was clinically relevant. The results of the Bland–Altman analysis of TBNK cells are given in Supplementary File 4.

### 3.4. Biochemical and Safety Analysis

In the test group, there was a statistically significant increase in platelet count (*p* = 0.017), absolute neutrophil count (*p* = 0.001), lymphocytes (*p* < 0.001), and leukocytes count (*p* < 0.001) at the end of the 30-day study period whereas hemoglobin, RBC, hematocrit, eosinophil, basophil, and monocyte did not have any significant difference. In the placebo group, the platelet was significantly increased (*p* = 0.006) but lymphocytes significantly decreased (*p* < 0.001) and other CBC parameters did not have any significant change. In the participants who crossed over to the test, there was a significant mean increase of lymphocytes (*p* = 0.008) at the end of 30 days extension period. In subjects who continued with the test product, there was a significant increase in hematocrit (*p* = 0.021), the counts of platelet (*p* = 0.009), neutrophils (*p* < 0.001), monocytes (*p* = 0.033), lymphocytes (*p* = 0.002), and leukocytes (*p* < 0.001) while the rest of the CBC parameters did not have any significant change. Serum creatinine in test group significantly decreased after 30 days (*p* = 0.020) and 60 days (0.007). The hematology and biochemical results are given in Table 4. There were no adverse events reported in both groups during the study periods.

## 4. Discussion

Immune health is well known to be affected by stress, sleep, and environmental factors. Environmental stressors can lead to fluctuations in immune function among individuals [6,7,8]. For thousands of years, Ayurveda has relied on Rasayana or “rejuvenating” herbs like WS that are considered to possess adaptogenic and anti-stress effects. Emerging evidence suggests these properties of WS may be related to its immunomodulatory effects. In particular, bioactive compounds known as withanolides in the root and leaf are studied for their immunomodulatory effects.

The present study showed WS root and leaf extract standardized for withanolide glycosides (withanoglycosides) can increase both immunoglobulins, as part of innate immunity, while increasing IFN-gamma and T-cells CD3+ and CD4+ in humans. These results suggest a prominent role in supporting innate and adaptive immunity in humans, which is vital for recognizing and responding appropriately to common bacteria, viruses, and allergens. The results from the present study are consistent with previously reported in vivo results, including an increase in the expression levels of T-helper 1 (Th1) cytokines, as well as CD4+ and CD8+ T cell counts. Similarly, enhanced natural killer (NK) cell activity in a dose-dependent manner with the prompt recovery of CD4+ T cells in immune-suppressed animals [9,10]. In the present study, the WS extract used in our study showed a significant increase in CD3+, CD4+, CD8+, CD19+, and NK cells with 30 days of treatment.

Several in vivo studies on WS suggest its ability to impact multiple layers of immune function which span both innate and adaptive immunity. Several animal studies have demonstrated that alcoholic extracts of WS enhance the production of white blood cells [11,12]. While many studies have been performed on Ashwagandha root, WS leaves, like the root, are abundant in withanolides, particularly withaferin A, a compound associated with potent immunomodulating and anti-inflammatory activity. Whole plant extracts of WS, including roots and leaves, increased immune cell (macrophage) activity, specifically lysosomal enzymes which function to detoxify and eliminate byproducts of immune cells. Alcoholic extracts of WS whole plant have also been found to augment the phagocytic activity of macrophages, reduce immune hypersensitivity reactions, and stimulate the generation of T lymphocyte immune cell [13].

The impact of WS on adaptive immunity may be particularly useful after exposure to pathogens, including bacteria and viruses. In mice administered a standardized WS leaf extract for 2 weeks experienced a promoted Th1 response by stimulating the expression of interferon-gamma and B cells while enhancing the expression of T cells CD3+, CD4+, and CD8+, along with co-stimulatory CD80 and integrins [9]. Malik et al. in 2007 found that the root extract of W.S was found to enhance cell-mediated immunity by predominantly enhancing Th1 immunity [14]. Taken together, it may be hypothesized from the available data that extracts of WS root and leaf may have greater immunomodulatory activity than either isolated plant part, although further research should be performed to directly compare the activities of root and leaf to isolated plant parts. (Supplementary File 5).

Khan et al. (2009) and Malik et al. (2007) have shown that both markers based chemically standardized root and leaf extract can stimulate the Th1 cell mediated and Th2 humoral immune response in BALB/c mice [9,14]. The results obtained are in line with earlier studies with WS, which showed a significant increase in the expression levels of CD3+, CD4+, CD8+, CD19+, CD45+, and NK cells with 30 days of treatment. There was a further significant increase in the level of these cells in the 30-day extension study as well, which has not gone beyond the normal level. This indicates positive immunomodulation with continued use of WS.

Two groups of cytokines are produced by different T helper (Th) lymphocytes which favor either cell-mediated and inflammatory immunity (Th1) or antibody-mediated humoral immunity (Th2). The Th2 cytokines, which include IL-4, generally promote B-cell function. These two cytokine groups are also antagonistic in that IFN-γ inhibits Th2 cytokine production, whereas IL-4 inhibits Th1 cytokine production. In general, the balance between Th1 and Th2 cytokines depends on many factors [15], among which are the nature of the antigen, the genetic background of the host, and the cytokines involved in the primary interaction of T cells with the antigen-presenting cells.

In our study, although the level of IFN-γ was increased in the WS group from baseline to day30 and from day30 to day 60, the increase was not so high as compared to the increase in the levels of IL4 (normal range IFN-γ (70–200 pg/mL), IL4 (4–10 pg/mL). This indicates that in WS treated group, there was an initial polarization of Th2 type of response (IL4) followed by Th1 type (IFN-γ). Thus, it can be inferred from these results that the WS intervention group had mounted an effective immune response of Th2 type (IL4). This increase in cytokine levels is also supported by an increase in the B-cells (CD19+).

The perception of seasonal stressors has the potential of compromising immune function unless countered. The pattern in adrenocortical activity suggests a circannual rhythm in cellular immunity, characterized by winter depression and a summer peak in T-cell function. Recent evidence suggests that immune function varies substantially on a seasonal basis [6,16] and this variation occurs independently of the fluctuations in pathogen prevalence. Thus, a postulated winter depression of cellular immune function hypothetically leads to a corresponding enhancement of B-cell function. In our study, this is evident from a decrease of B-cell counts and an increase in antibody in the placebo group whereas in the test group there was significant increase in the lymphocytes and immunoglobulin count.

Sleep is undoubtedly a prominent behavioral state of living beings and a likely modulator of the immune function. Both acute and chronic deprivation is associated with immune changes. WS has been clinically shown to improve the overall sleep quality and sleep efficiency in subjects with non-restorative sleep which is commonly associated with insomnia and lack of restful sleep [17].

The study done by MacMurray et al. (1983) [18] shows: (1) an inverse relationship between ambient temperature and serum IgG levels; (2) an elevated level of serum IgG in the winter as compared to summer; (3) generally higher levels of serum IgA and IgM in the winter; (4) an inverse relationship between B-cell and T-cell activity; and (5) a depressed T-cell activity in the winter which is accompanied by significantly elevated B-cell activity. In our study, it can be seen in the placebo group that there is a non-significant increase in all the subtypes of IgG except IgG2, IgA, and IgM while there is a significant decrease in the B cells and T cells. In a study conducted in the Netherlands, the number of circulating CD8+ T-cells was reportedly lowest during the winter months [19]. In effect, energy deficit will decrease immune cell counts, and thus susceptibility to infections increases. WS is reported to stimulate the cell-mediated immunity, IgM, and IgG and a noticeable improvement in proliferation and differentiation of lymphocytes as indicated by lymphocyte surface markers of T cells (CD3+, CD4+, and CD8+) and B cells (CD19+) [9,10]. This supports our findings of a significant increase of the T cells, B cells, and NK cells in the WS group and also in the placebo group crossed over to the WS group in our study.

The low-circulating concentration of DHEA has been postulated as a concomitant of chronic illness, infection, and stress [20,21]. The present study is more relevant in this context since ashwagandha extract standardized with withanolide glycosides was associated with increases in DHEA-S and lowering of cortisol (stress hormone) levels [22].

Many variables are capable of modulating the effects of stressors on the human immune system and neither a genetic predisposition, exposure to a pathogen nor the experience of a stressful event is able, by itself, to trigger a predictable progression of the disease. Instead, each of these contributes to the psychological and physiological responses of the organism and ultimately determines the balance point between good health and disease. Hence a holistic approach is best suited to develop immunity for which WS, the ayurvedic Rasayana ingredient with clinically proven efficacy in improving immunity will be an ideal approach.

### Limitations and Future Directions of Study

The present study was done in a sample size of 12 healthy middle aged Indian men and women in each arm. Although the results showed statistical significance for a number of the biomarkers analyzed, additional studies with larger sample size would be necessary to confirm these pilot study findings. In this study, dietary, environmental, occupational, lifestyle and general health conditions were not individually monitored, and their impact on the results was not determined. However, the follow-up cross-over extension study does help to rationalize any effects. Future studies to address these limitations will help confirm these findings.

## 5. Conclusions

This pilot, randomized, and controlled clinical study demonstrates for the first time that the leaf and root extract of WS standardized with withanolide glycosides possesses potent immune-stimulatory properties. It establishes Th2 up-regulating activity as evidenced by enhanced secretion of IL4 along with the enhanced expression of Th1 (IFN-γ) strongly suggesting the role of WS in conditions where Th1/Th2 modulation is required. WS enhanced the proliferation of CD4+/CD8+ without altering its ratio and NK cells along with an increased expression of CD3+/CD19+/CD45+ and increased the circulating antibody and antibody-forming cells. The results of this study demonstrate that WS of defined chemical signature with its immune-stimulatory activities is a valuable addition in the immunity-boosting herbal supplements. These effects span both innate and adaptive immunity, which may partially explain its traditional use as a Rasayana or rejuvenating herb with anti-stress properties.

## Figures and Tables

**Table 1:** Comparison of the baseline demographics of the test group and placebo group.

	Test Group	Placebo Group	Between the Groups
	Mean ± SE	Mean ± SE	*p* Value *
Age (years)	53.92 ± 1.83	56.17 ± 2.39	0.461
Height (cm)	156.50 ± 1.69	156.75 ± 2.12	0.929
Weight (kg)	65.13 ± 2.85	67.75 ± 2.07	0.467
BMI (kg/m^2^)	26.47 ± 0.80	27.51 ± 0.42	0.258

* *t*-test for independent groups.

**Table 2:** Comparison of the level of immunoglobulins (mg/dL) in serum samples of test and placebo group at day 0, 30 and 60 using flow cytometry.

	Test Group			Within Group Analysis of Test Group						Placebo Group		P➔T **	Within Group Analysis				Between Group Analysis	
												Crossover Group	Placebo Group		P➔T ** Crossover Group			
	Day 0	Day 30	Day 60	Day 0–30		Day 30–60		Day 0–60		Day 0	Day 30	Day 60	Day 0–30		Day 30–60		Day 0	Day 30
	Mean ± SE	Mean ± SE	Mean ± SE	% Change	*p* Value ^#^	% Change	*p* Value ^#^	% Change	*p* Value ^#^	Mean ± SE	Mean ± SE	Mean ± SE	% Change	*p* Value ^#^	% Change	*p* Value ^#^	*p* Value ^$^	*p* Value ^$^
IgA	170.06 ± 16.99	180.2 ± 18.28	184.13 ± 18.66	5.96	<0.001	2.18	<0.001	8.27	<0.001	169.9 ± 14.95	174.88 ± 15.76	183.62 ± 16.55	2.93	0.764	5	<0.001	0.994	0.828
IgG2	212.16 ± 16.4	217.5 ± 16.71	224.69 ± 17.25	2.52	<0.001	3.31	<0.001	5.91	<0.001	205.97 ± 21.36	183.74 ± 15.4	189.25 ± 15.86	−10.79	0.286	3	<0.001	0.82	0.151
IgG3	47.63 ± 3.72	48.83 ± 3.81	50.87 ± 3.97	2.52	<0.001	4.18	<0.001	6.8	<0.001	43.03 ± 3.5	50.36 ± 5.38	50.76 ± 5.42	17.03	0.172	0.79	<0.001	0.377	0.819
IgG4	24.96 ± 2.64	25.97 ± 2.76	27.37 ± 2.90	4.05	<0.001	5.39	<0.001	9.66	<0.001	24.95 ± 2.67	26.42 ± 2.47	26.95 ± 2.52	5.89	0.704	2.01	<0.001	0.997	0.906
IgG	801.44 ± 59.19	842.23 ± 62.2	894.14 ± 66.12	5.09	<0.001	6.16	<0.001	11.57	<0.001	822.99 ± 69.52	836.58 ± 70.88	881.75 ± 74.71	1.65	0.879	5.4	<0.001	0.816	0.953
IgM	183.9 ± 34.41	185.43 ± 34.67	200.7 ± 37.54	0.83	<0.001	8.23	<0.001	9.14	<0.001	184.14 ± 55.64	185.17 ± 55.97	186.41 ± 56.34	0.56	0.99	0.67	0.002 *	0.997	0.299 *

^#^ Within group significance using the *t*-test (equal variances). ^$^ Between the group significance using the *t*-test for independent groups. * *p* value using Mann–Whitney test. **P➔T** ** Placebo group crossed over to the test group in the extension study period.

**Table 3:** Comparison of the level of T cells, B cells, NK cells count (cells/µL) in whole blood and cytokines IL4 and IFNγ level (pg/mL) in serum samples of test and placebo group at day 0, 30 and 60 using flow cytometry.

	Test Group			Within Group Analysis of Test Group						Placebo Group		P➔T ** Crossover Group	Within Group Analysis				Between Group Analysis	
													Placebo Group		P➔T ** Crossover Group			
	Day 0	Day 30	Day 60	Day 0–30		Day 30–60		Day 0–60		Day 0	Day 30	Day 60	Day 0–30		Day 30–60		Day 0	Day 30
	Mean ± SE	Mean ± SE	Mean ± SE	% Change	*p* Value ^#^	% Change	*p* Value ^#^	% Change	*p* Value ^#^	Mean ± SE	Mean ± SE	Mean ± SE	% Change	*p* Value ^#^	% Change	*p* Value ^#^	*p* Value ^$^	*p* Value ^$^
CD45	2148.03 ± 159.54	2492.41 ± 105.24	2854.3 ± 92.85	12.07	0.032	14.52	<0.001	32.88	0.001	2201.22 ± 191.39	1852.96 ± 122.25	2255.87 ± 132.64	−15.44	0.009	21.74	0.036	0.833	0.001
CD3	1334.71 ± 98.24	1699.4 ± 76.06	1874.02 ± 90.06	19.46	0.001	10.28	0.026	40.41	0.002	1595.16 ± 109.60	1253.94 ± 68.87	1550.93 ± 96.93	−22.00	0.002	23.68	0.01	0.091	<0.001
CD4	738.23 ± 54.71	918.25 ± 49.85	1042.82 ± 49.84	17.26	0.003	13.57	0.018	41.26	0.001	876.88 ± 59.59	669.3 ± 47.57	860.34 ± 54.87	−24.13	0.001	28.54	0.005	0.101	0.002
CD8	498.95 ± 39.11	603.31 ± 24.77	716.47 ± 52.85	14.57	0.005	18.76	0.028	43.6	0.003	604.37 ± 53.12	455.06 ± 24.24	594.18 ± 43.47	−25.13	0.02	30.57	0.01	0.124	<0.001
CD19	661.53 ± 29.81	761.98 ± 30.87	974.39 ± 63.75	10.31	0.034	27.88	0.021	47.29	0.004	751.06 ± 32.04	734.83 ± 28.08	870.1 ± 42.12	−1.87	0.733	18.41	0.006	0.053	0.522
NK	361.92 ± 66.06	467.36 ± 68.41	552.68 ± 62.51	19.08	0.046	18.26	0.015	52.71	<0.001	341.95 ± 60.83	341.26 ± 42.73	465.47 ± 35.37	−0.15	0.989	36.4	0.027	0.826	0.132
IFNγ	72.15 ± 7.25	100.60 ± 10.67	111.61 ± 7.64	39.44	<0.001	10.94	0.056	54.69	<0.001	73.36 ± 6.11	72.06 ± 5.70	97.65 ± 9.01	−1.77	0.749	35.51	<0.001	0.899	0.028
IL4	3.92 ± 1.42	7.16 ± 1.51	9.30 ± 1.13	82.53	0.047	29.89	0.053	137.06	0.004	4.20 ± 1.94	3.21 ± 1.35	6.29 ± 0.80	−23.50	0.814 *	95.87	0.038 *	0.817	0.03

^#^ Within group significance using the *t*-test (equal variances). ^$^ Between the group significance using the *t*-test for independent groups. * *p* value using Mann–Whitney test. **P➔T** ** Placebo group crossed over to the test group in the extension study period.

**Table 4:** Comparison of biochemical and safety parameters in plasma samples of test and placebo group at day 0, 30 and 60.

	Test Group			Within Group Analysis of Test Group		Placebo		P➔T ** Crossover Group	Within Group Analysis	
									Placebo	P➔T ** Crossover Group
	Day 0	Day 30	Day 60	Day 0–30	Day 0–60	Day 0	Day 30	Day 60	Day 0–30	Day 30–60
	Mean + SE	Mean + SE	Mean + SE	*p*-Value ^#^	*p*-Value ^#^	Mean + SE	Mean + SE	Mean + SE	*p*-Value ^#^	*p*-Value ^#^
Red Blood Cell	4.46 ± 0.08	4.58 ± 0.10	4.49 ± 0.08	0.244	0.755	4.52 ± 0.07	4.56 ± 0.05	4.55 ± 0.10	0.587	0.931
(10^12^/L)										
Hemoglobin (g/dl)	12.98 ± 0.27	13.04 ± 0.29	13.00 ± 0.22	0.792	0.946	12.62 ± 0.32	12.38 ± 0.20	12.49 ± 0.22	0.446	0.567
Hematocrit (%)	44.00 ± 0.85	42.18 ± 1.02	41.34 ± 1.05	0.078	0.021	40.92 ± 1.02	40.78 ± 0.86	41.76 ± 0.84	0.871	0.432
Platelet count (10^9^/L)	248.42 ± 19.20	286.92 ± 21.33	299.25 ± 18.36	0.017	0.009	226.08 ± 12.12	288.08 ± 14.95	301.75 ± 16.56	0.006	0.511
Neutrophil count (cells/mm^3^)	3611.73 ± 92.30	3930.02 ± 73.19	4089.26 ± 126.28	0.001	<0.001	3658.69 ± 138.22	3624.15 ± 107.61	3561.05 ± 118.30	0.772	0.716
Eosinophil count (cells/mm^3^)	149.72 ± 6.01	150.10 ± 5.97	155.05 ± 4.84	0.945	0.241	142.69 ± 5.13	141.24 ± 4.11	142.16 ± 5.29	0.846	0.859
Basophil count	55.46 ± 5.84	61.42 ± 6.14	55.67 ± 5.32	0.358	0.969	57.58 ± 5.84	56.38 ± 4.76	58.09 ± 5.69	0.894	0.826
(cells/mm^3^)										
Monocyte count (cells/mm^3^)	459.25 ± 15.58	457.76 ± 16.11	495.37 ± 14.70	0.899	0.033	464.19 ± 17.59	442.73 ± 14.28	459.03 ± 18.53	0.267	0.532
Leukocytes	5706.67 ± 126.82	5875.81 ± 128.18	6095.51 ± 140.05	<0.001	<0.001	5701.67 ± 138.70	5630.00 ± 115.13	5496.67 ± 162.25	0.087	0.529
(cells/mm^3^)										
Lymphocytes	1830.45 ± 130.18	2297.50 ± 152.05	2656.51 ± 210.26	0.004	0.002	1711.42 ± 46.66	1131.61 ± 95.09	1644.33 ± 132.33	<0.001	0.008
(cells/mm^3^)										
Total Cholesterol (mg/dL)	184.17 ± 7.83	180.67 ± 8.37	171.67 ± 6.88	0.561	0.172	186.83 ± 3.95	168.25 ± 4.11	172.17 ± 4.39	0.009	0.424
Triglycerides (mg/dL)	156.75 ± 5.89	154.68 ± 5.56	150.83 ± 5.77	0.529	0.267	161.00 ± 5.66	151.62 ± 3.59	152.33 ± 4.74	0.077	0.661
LDL Cholesterol (mg/dL)	72.00 ± 3.08	67.58 ± 3.34	63.33 ± 3.31	0.183	0.013	71.75 ± 3.08	67.50 ± 2.66	67.28 ± 1.70	0.035	0.096
VLDL Cholesterol (mg/dL)	32.67 ± 1.32	32.00 ± 1.84	32.36 ± 2.38	0.627	0.883	29.25 ± 1.42	30.28 ± 1.50	27.87 ± 1.52	0.599	0.044
HDL Cholesterol (mg/dL)	32.17 ± 0.84	33.03 ± 0.71	31.08 ± 1.16	0.308	0.469	34.42 ± 4.04	33.03 ± 0.73	32.25 ± 0.66	0.769	0.334
SGOT (U/L)	22.28 ± 1.35	26.25 ± 1.83	26.08 ± 1.07	0.076	0.021	24.00 ± 1.31	27.30 ± 1.52	26.98 ± 1.27	0.079	0.647
SGPT (U/L)	46.20 ± 3.00	51.71 ± 2.43	52.94 ± 2.70	0.115	0.067	48.60 ± 3.82	52.15 ± 3.27	51.33 ± 2.55	0.382	0.711
Blood Urea (mg/dL)	15.32 ± 0.67	15.02 ± 0.69	14.42 ± 0.48	0.499	0.192	14.58 ± 0.87	14.83 ± 0.54	14.95 ± 0.72	0.799	0.792
Serum creatinine (mg/dL)	0.73 ± 0.03	0.56 ± 0.06	0.57 ± 0.05	0.02	0.007	0.72 ± 0.02	0.66 ± 0.06	0.68 ± 0.07	0.408	0.634
Fasting Blood Sugar (mg/dL)	88.17 ± 2.55	88.25 ± 2.55	88.62 ± 2.71	0.984	0.907	90.67 ± 2.60	90.37 ± 2.04	89.19 ± 2.21	0.914	0.561
Systolic Blood Pressure (mm/Hg)	127.08 ± 0.96	126.50 ± 0.89	126.92 ± 0.75	0.669	0.845	128.25 ± 1.56	127.75 ± 1.02	126.92 ± 0.84	0.784	0.372
Diastolic Blood Pressure (mm/Hg)	84.42 ± 1.18	84.67 ± 0.88	85.33 ± 1.09	0.891	0.613	83.92 ± 1.50	85.67 ± 0.87	85.50 ± 1.00	0.358	0.883
Pulse rate (beats/min)	74.25 ± 1.90	71.75 ± 1.15	73.42 ± 1.29	0.106	0.493	75.58 ± 1.11	74.83 ± 1.05	72.42 ± 0.82	0.529	0.092
Temperature (°C)	36.74 ± 0.11	36.63 ± 0.09	36.53 ± 0.09	0.463	0.138	36.79 ± 0.13	36.64 ± 0.12	36.41 ± 0.07	0.523	0.14

^#^ Within group significance using the *t*-test (equal variances). ** Placebo group crossed over to the test group in the extension study period.

## Supplementary Materials

The following are available online at https://www.mdpi.com/article/10.3390/jcm10163644/s1. Supplementary File 1 Gating methodology of Immunoglobulins, cytokines and TBNK cells; Supplementary File 2 Bland Altman analysis of immunoglobulins; Supplementary File 3 Bland Altman analysis of cytokines; Supplementary File 4 Bland Altman analysis of TBNK cells; Supplementary File 5 Studies on Ashwagandha. References [9,10,11,12,13,14,23,24,25,26,27,28] are cited in the supplementary materials.

## Clinical Trial Registration

Clinical Trials Registry—India (CTRI/2018/07/014792 [Registered on: 10/07/2018]—Trial Registered Prospectively). http://www.ctri.nic.in/Clinicaltrials/pdf_generate.php?trialid=22690&EncHid=&modid=&compid=%27,%2722690det%27.
