# Supplementary material for: Immunomodulatory Effect of Withania somnifera (Ashwagandha) Extract—A Randomized, Double-Blind, Placebo Controlled Trial with an Open Label Extension on Healthy Participants
Source: J Clin Med. 2021 Aug 18;10(16):3644. doi: 10.3390/jcm10163644 (PMC8397213; doi:10.3390/jcm10163644)
Supplement: Supplementary file 1 [file jcm-10-03644-s001.zip › Supplementary file 1 - final.pdf]

Supplementary file 1. Gating Methodology of Immunoglobulins, Cytokines and TBNK cells.

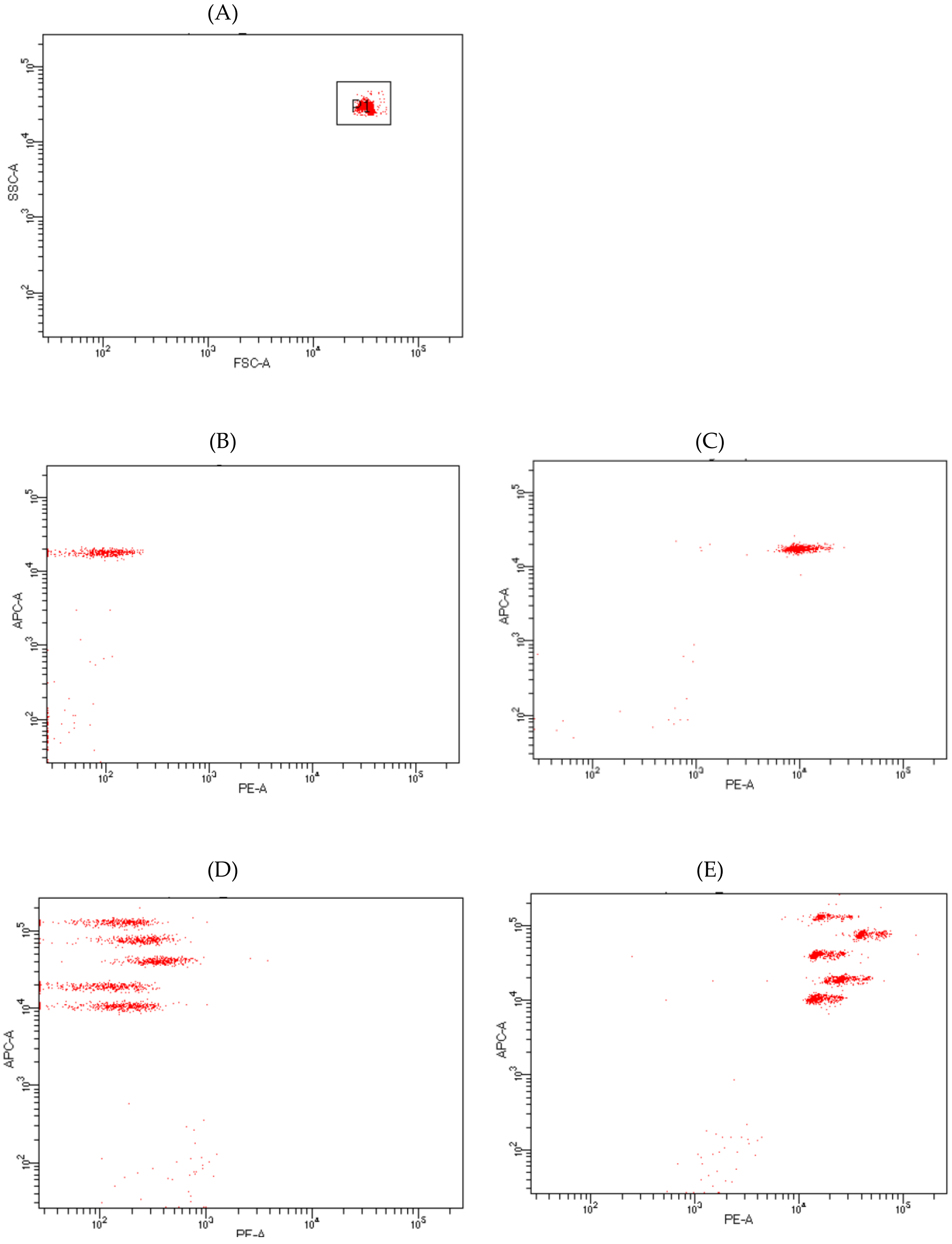

Figure S1. Demonstration of Gating Methodology of Immunoglobulins using BD FACSDiva 8.5 software. (A) The primary singlet gate of Immunoglobulins on forward scattering vs side scattering plot (FSC vs SSC plot). Secondary gating on PE vs APC plot stained with phycoerythrin (PE) or allophycocyanin (APC)–conjugated antibodies (B) negative control for IgG (C) positive control for IgG (D) negative control for IgG2, IgG3, IgG4, IgM, IgA (E) positive control for IgG2, IgG3, IgG4, IgM, IgA.

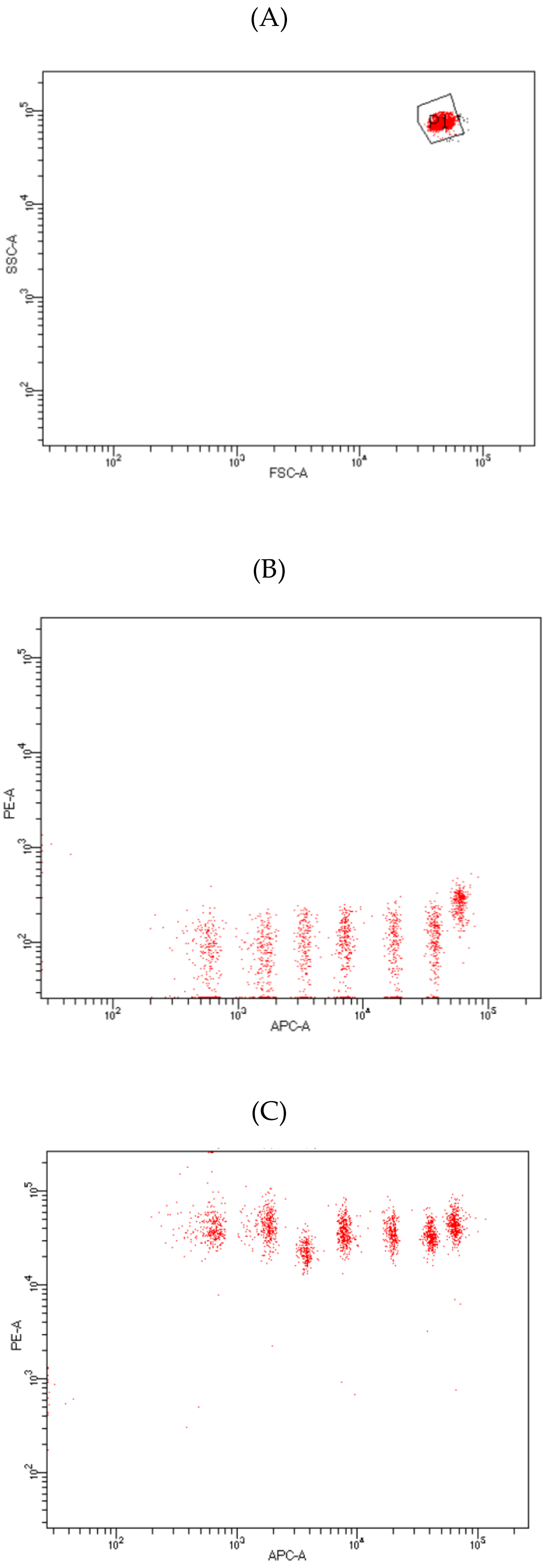

Figure S2. Demonstration of the gating strategy of cytokines on BD FACSDiva 8.5 software. (A) The primary singlet gate for cytokines on FSC vs SSC plot. The secondary gating on PE vs APC plot. (B) Negative control for cytokines IFN-γ & IL-4. (C) positive control for cytokines IFN-γ & IL-4. The 2nd row from left is IFN-γ and the 6th row is the IL-4.

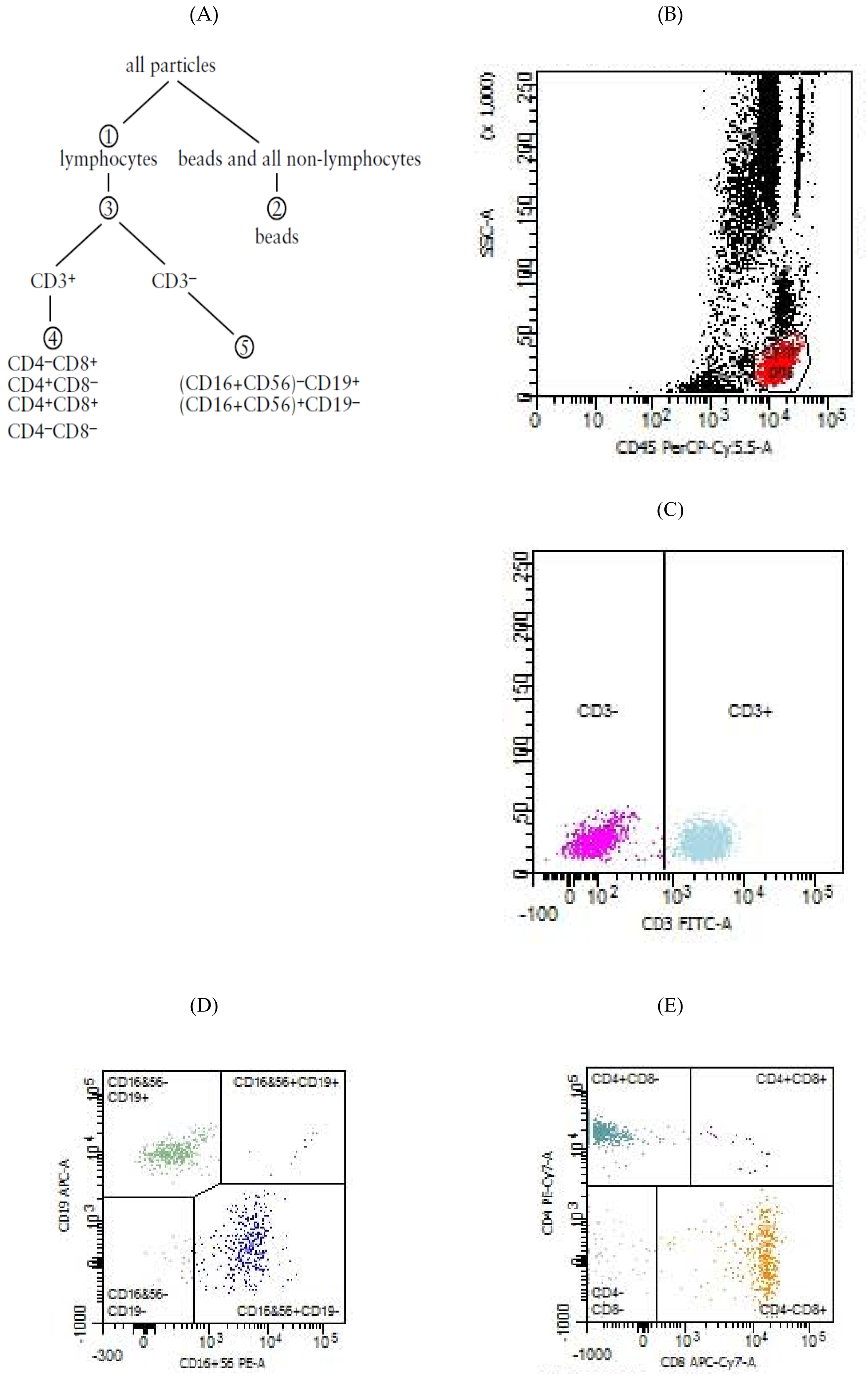
(A)
all particles
① lymphocytes
beads and all non-lymphocytes
② beads
③
CD3+
CD3−
④
CD4−CD8+
CD4+CD8−
CD4+CD8+
CD4−CD8−
⑤
(CD16+CD56)−CD19+
(CD16+CD56)+CD19−
(B)
SSC-A
(x 1,000)
CD45 PerCP-Cy5.5-A
(C)
CD3-
CD3+
CD3 FITC-A
(D)
CD16&56-CD19+
CD16&56+CD19+
CD16&56-CD19-
CD16&56+CD19-
CD19 APC-A
CD16+56 PE-A
(E)
CD4+CD8-
CD4+CD8+
CD4-CD8-
CD4-CD8+
CD4 PE-Cy7-A
CD8 APC-Cy7-A

Figure S3. Demonstration of the gating methodology of T-cells, B-cells and NK-cells on BD FACSCanto™ software v3.1with multicheck control. (A) Flowchart of particle resolution. BD Multitest reagents employ fluorescence triggering, allowing direct fluorescence gating of the lymphocyte population thereby reducing contamination with debris. (B) The primary gating based on CD45 (SSC-A vs CD45 plot) stained with fluorochrome-labeled (PerCP-Cy*) antibodies that specifically bind to leucocyte surface antigens. The red portion is the lymphocyte population appearing as a bright, compact cluster with low SSC. (C) The secondary gating is based on CD3+ & CD3^{-} (SSC-A vs CD3 plot, labeled by FITC**). (D) The tertiary gating applied on CD3+ to resolve the CD4± and CD8±, (CD4 vs CD8 plot). (E) The tertiary gating applied on CD3- resolved to (CD16 CD56) ± & CD19±. Side Scatter is on Linear Scale and other parameters are on Log Scale. PerCP–Cy™* − peridinin chlorophyll protein, which is excited by the 488-nm line of an Argon ion laser and serves as the energy donor, coupled to the cyanine dye Cy™5.5, which acts as the energy acceptor and fluoresces at 695 nm. FITC ** − fluorescein isothiocyanate Blue 488 nm. PE-labeled CD16, CD56, PE-Cy™7– Phycoerythrin labeled CD4 (Blue 488 nm, Green 532 nm, Yellow/Green 561 nm), APC-Allophycocyanin labeled CD19 (Red 633 nm), APC-Cy7 − Allophycocyanin labeled CD8.
