# Supplementary material for: Immunomodulatory Effect of Withania somnifera (Ashwagandha) Extract—A Randomized, Double-Blind, Placebo Controlled Trial with an Open Label Extension on Healthy Participants
Source: J Clin Med. 2021 Aug 18;10(16):3644. doi: 10.3390/jcm10163644 (PMC8397213; doi:10.3390/jcm10163644)
Supplement: Supplementary file 1 [file jcm-10-03644-s001.zip › supplementary file 2 - final.pdf]

Supplementary file 2. Bland-Altman Analysis of Immunoglobulins

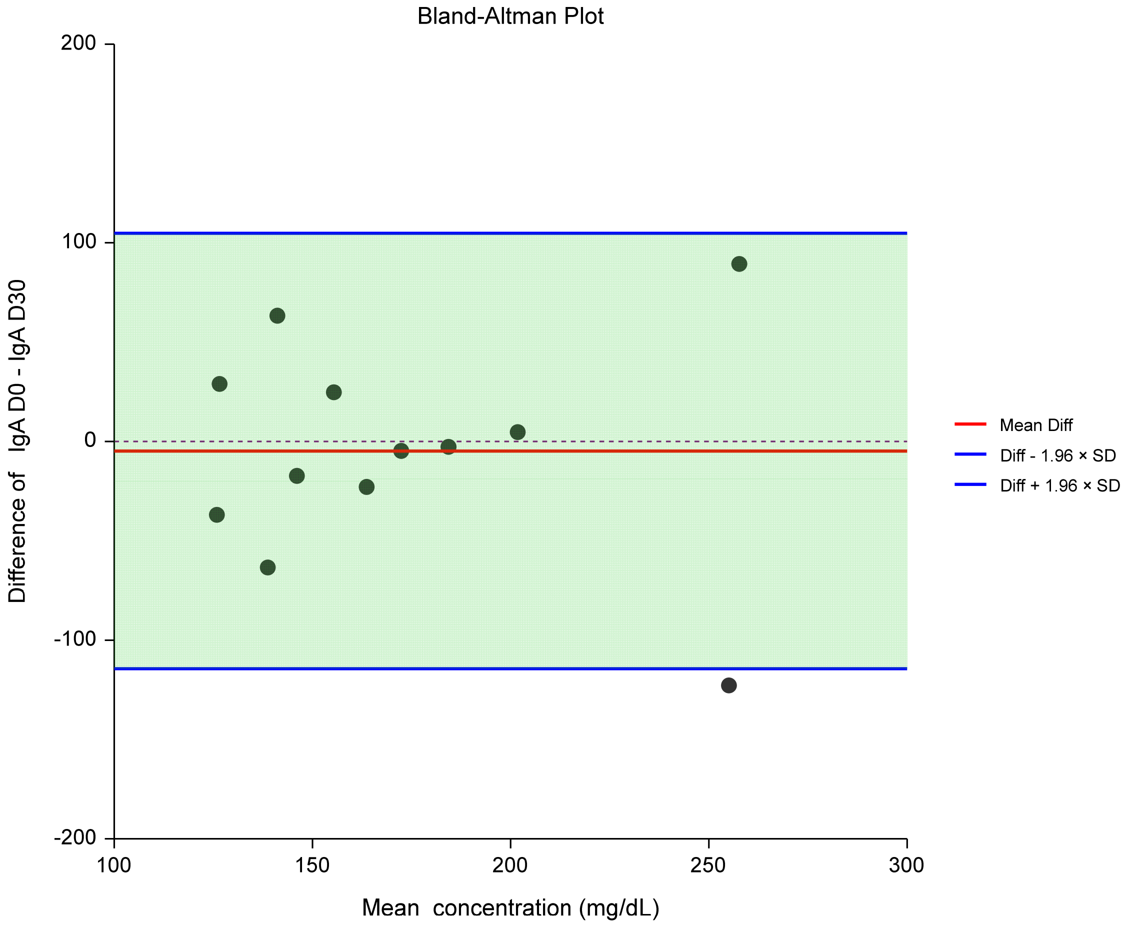

Figure S1. Bland-Altman plot of concentration of IgA for Day 0 and Day 30 in placebo group.

Table S1. Bias and Limits of Agreement of concentration of IgA for Day 0 and Day 30 in placebo group.

Parameter	Count	Value	Standard Deviation	95.0% LCL of Value	95.0% UCL of Value
Bias (Difference)	12	−4.97	55.99	−40.55	30.60
Lower Limit of Agreement	12	−114.72	28.44	−177.31	−52.13
Upper Limit of Agreement	12	104.77	28.44	42.18	167.36

Limits of Agreement = Difference ± 1.96 × (Standard Deviation of Difference).

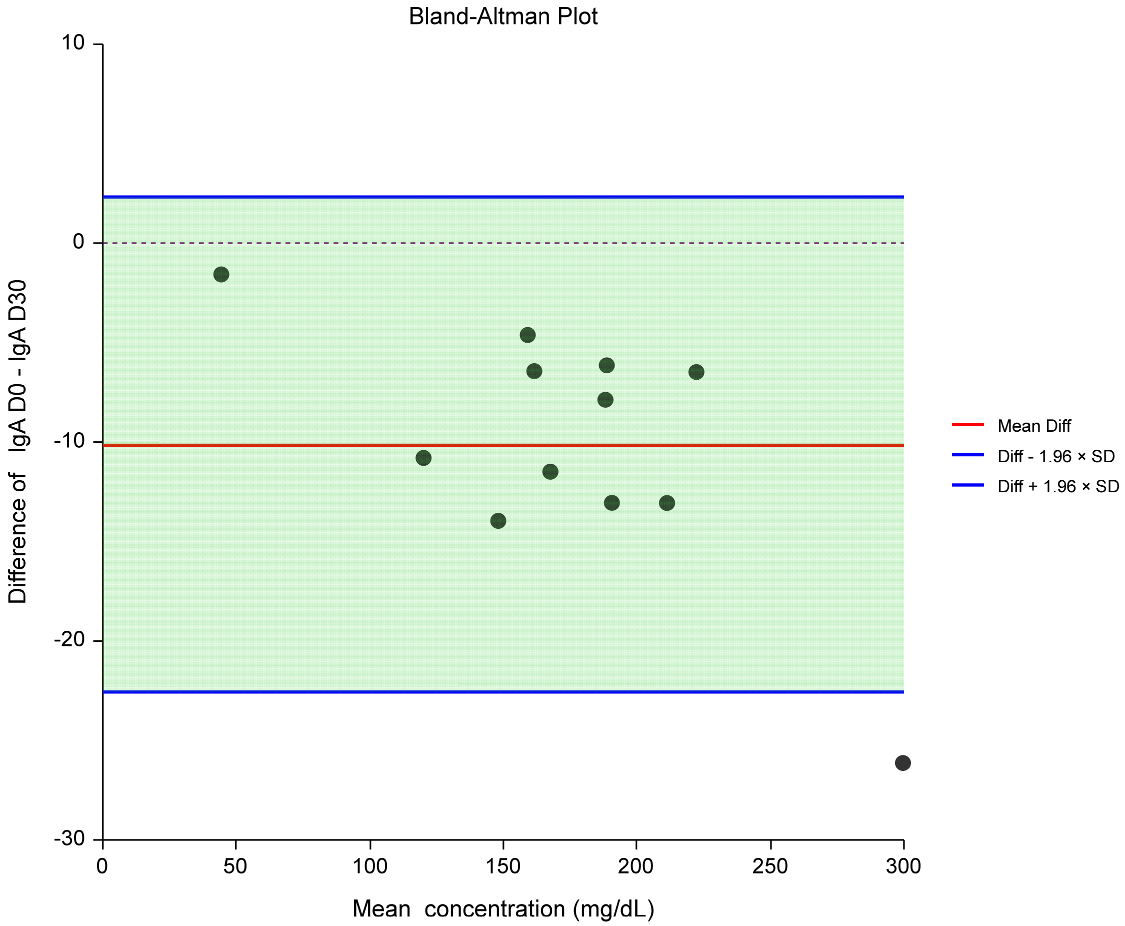

Figure S2. Bland-Altman plot of concentration of IgA for Day 0 and Day 30 in test group.

Table S2. Bias and Limits of Agreement of concentration of IgA for Day 0 and Day 30 in test group.

Parameter	Count	Value	Standard Deviation	95.0% LCL of Value	95.0% UCL of Value
Bias (Difference)	12	−10.13	6.34	−14.16	−6.11
Lower Limit of Agreement	12	−22.55	3.22	−29.64	−15.47
Upper Limit of Agreement	12	2.28	3.22	−4.80	9.37

Limits of Agreement = Difference ± 1.96 × (Standard Deviation of Difference).

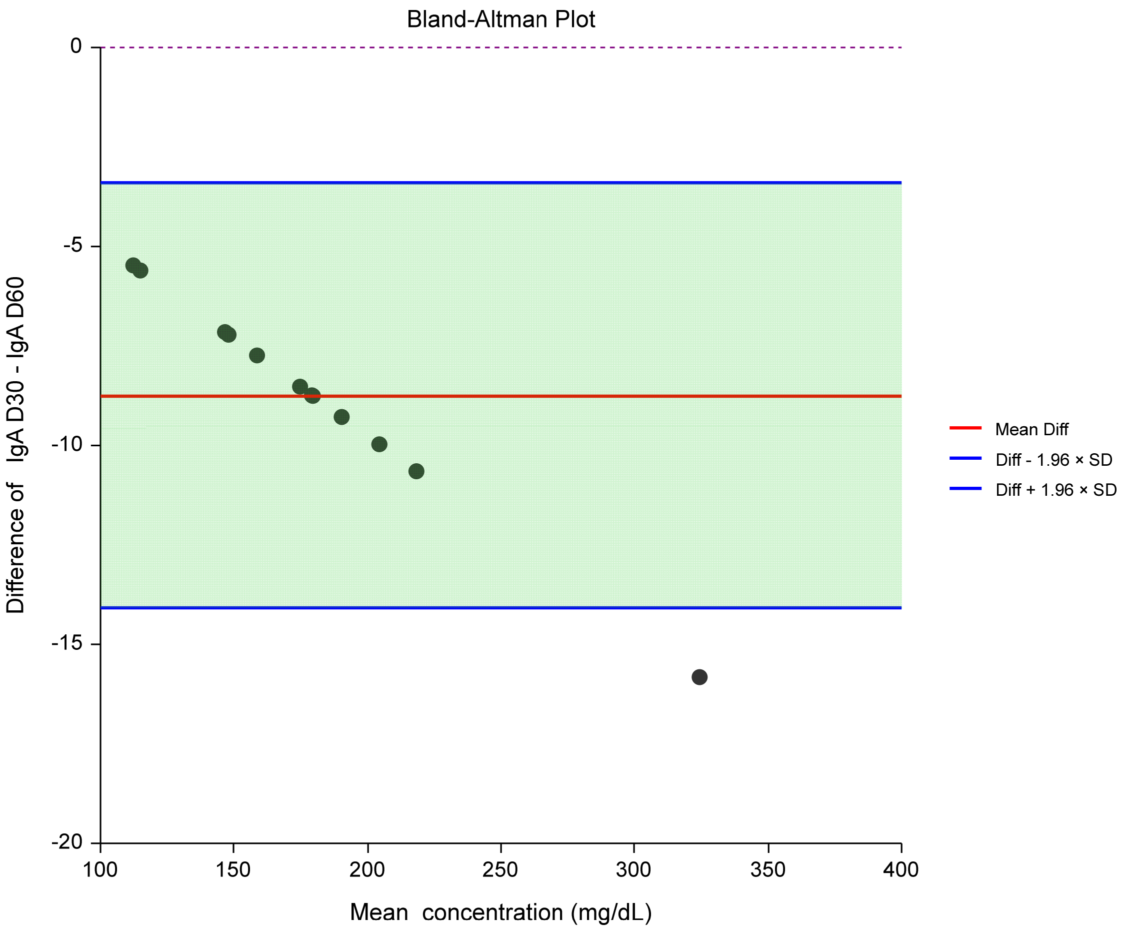

Figure S3. Bland-Altman plot of concentration of IgA for Day 30 and Day 60 in placebo crossed over to test group.

Table S3. Bias and Limits of Agreement of concentration of IgA for Day 30 and Day 60 in placebo crossed over to test group.

Parameter	Count	Value	Standard Deviation	95.0% LCL of Value	95.0% UCL of Value
Bias (Difference)	12	−8.74	2.73	−10.48	−7.01
Lower Limit of Agreement	12	−14.10	1.39	−17.15	−11.04
Upper Limit of Agreement	12	−3.39	1.39	−6.44	−0.34

Limits of Agreement = Difference ± 1.96 × (Standard Deviation of Difference).

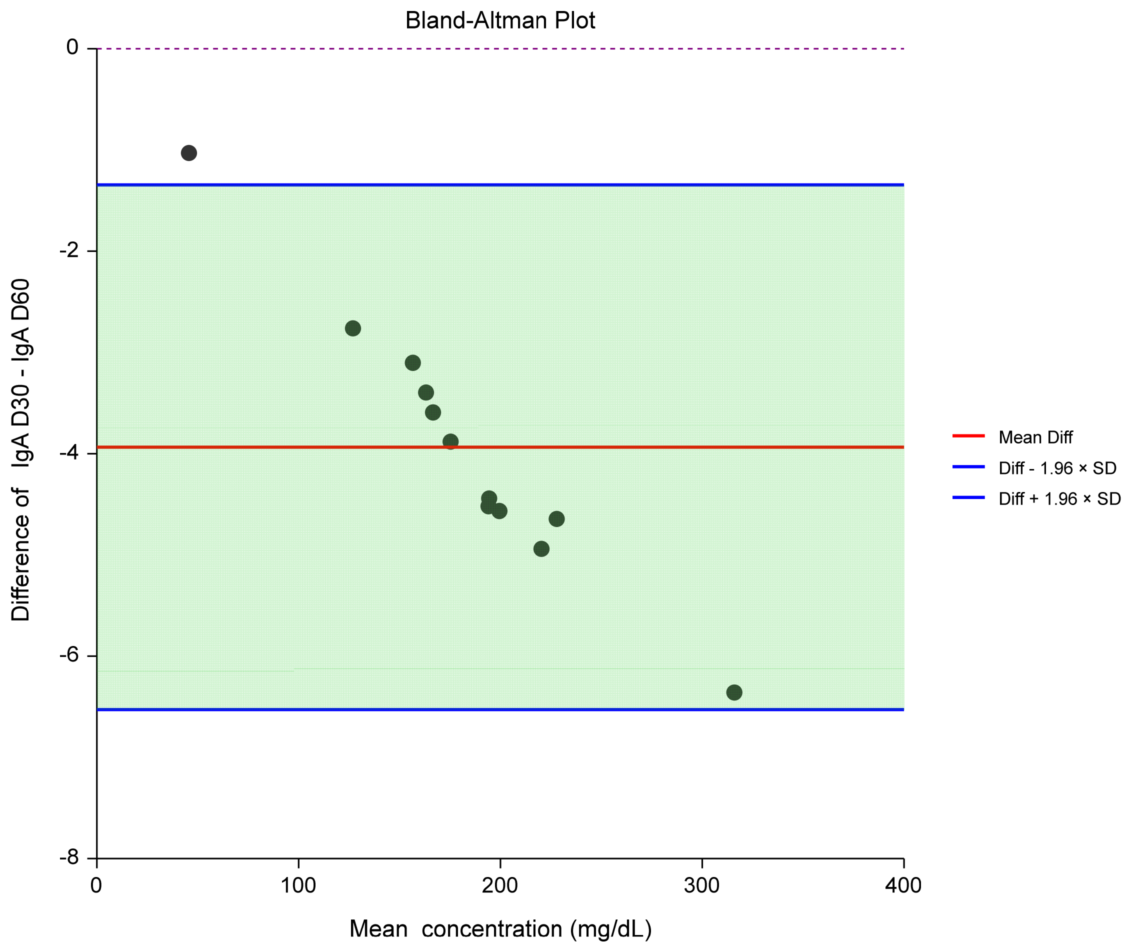

Figure S4. Bland-Altman plot of concentration of IgA for Day 30 and Day 60 in test group.

Table S4. Bias and Limits of Agreement of concentration of IgA for Day 30 and Day 60 in test group.

Parameter	Count	Value	Standard Deviation	95.0% LCL of Value	95.0% UCL of Value
Bias (Difference)	12	−3.94	1.33	−4.78	−3.09
Lower Limit of Agreement	12	−6.53	0.67	−8.01	−5.05
Upper Limit of Agreement	12	−1.34	0.67	−2.82	0.14

Limits of Agreement = Difference ± 1.96 × (Standard Deviation of Difference).

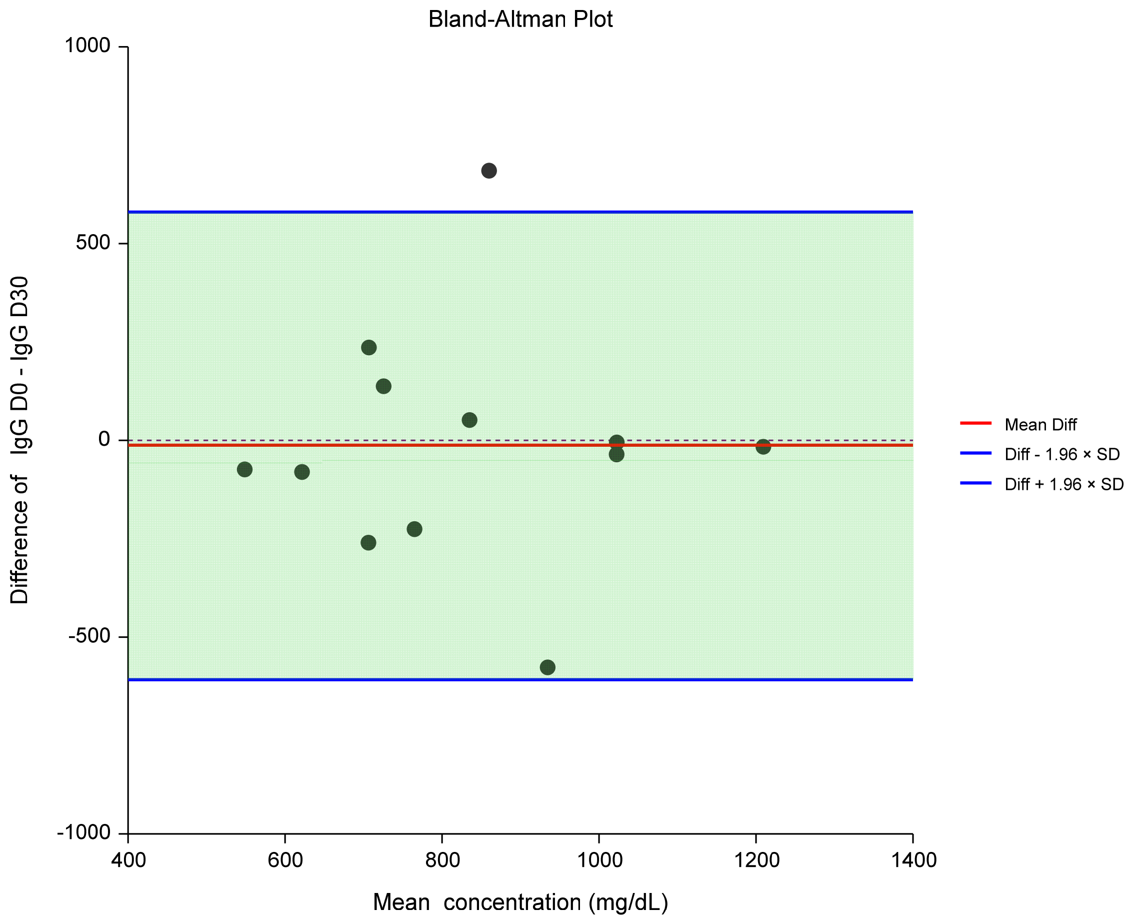

Figure S5. Bland-Altman plot of concentration of IgG for Day 0 and Day 30 in placebo group.

Table S5. Bias and Limits of Agreement of concentration of IgG for Day 0 and Day 30 in placebo group.

Parameter	Count	Value	Standard Deviation	95.0% LCL of Value	95.0% UCL of Value
Bias (Difference)	12	−13.58	302.92	−206.05	178.88
Lower Limit of Agreement	12	−607.31	153.85	−945.93	−268.69
Upper Limit of Agreement	12	580.14	153.85	241.52	918.77

Limits of Agreement = Difference ± 1.96 × (Standard Deviation of Difference).

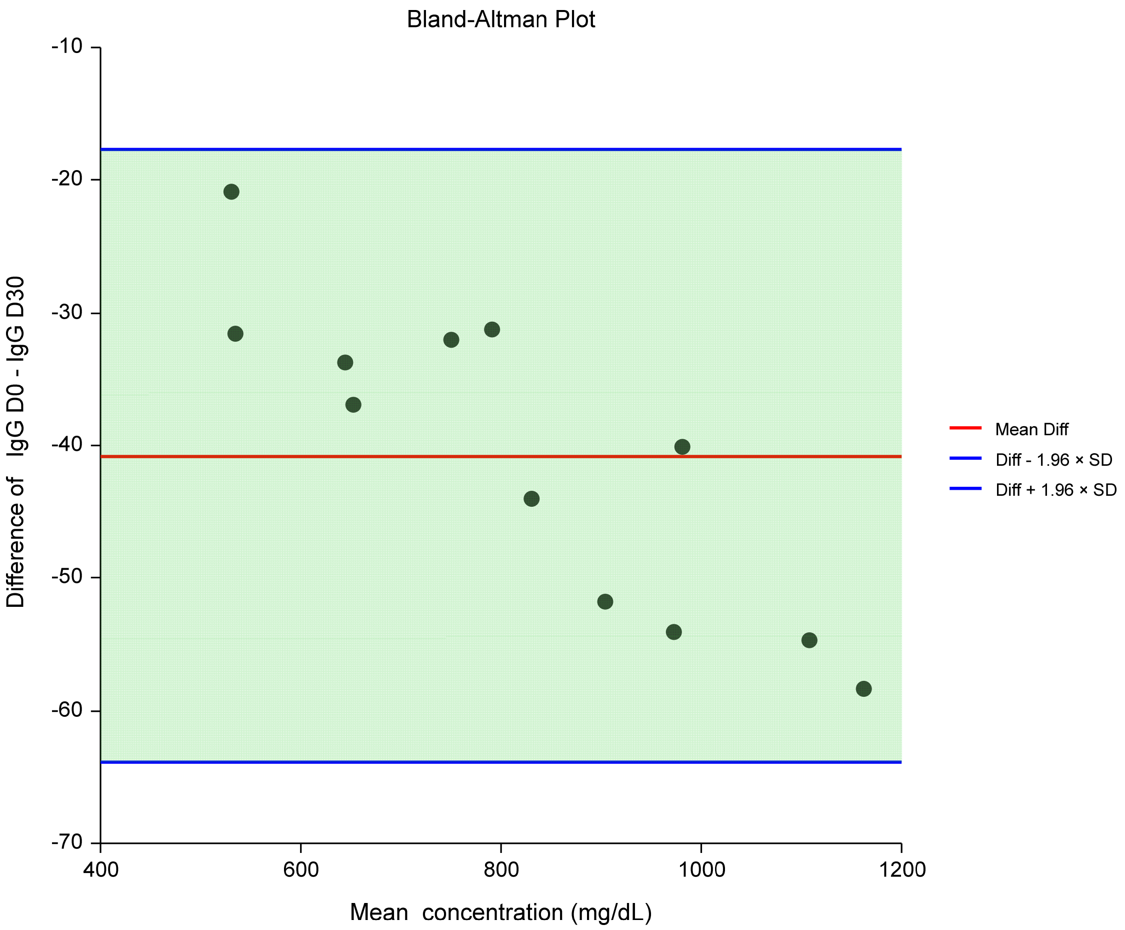

Figure S6. Bland-Altman plot of concentration of IgG for Day 0 and Day 30 in test group.

Table S6. Bias and Limits of Agreement of concentration of IgG for Day 0 and Day 30 in test group.

Parameter	Count	Value	Standard Deviation	95.0% LCL of Value	95.0% UCL of Value
Bias (Difference)	12	−40.79	11.77	−48.26	−33.31
Lower Limit of Agreement	12	−63.85	5.98	−77.00	−50.70
Upper Limit of Agreement	12	−17.72	5.98	−30.88	−4.57

Limits of Agreement = Difference ± 1.96 × (Standard Deviation of Difference).

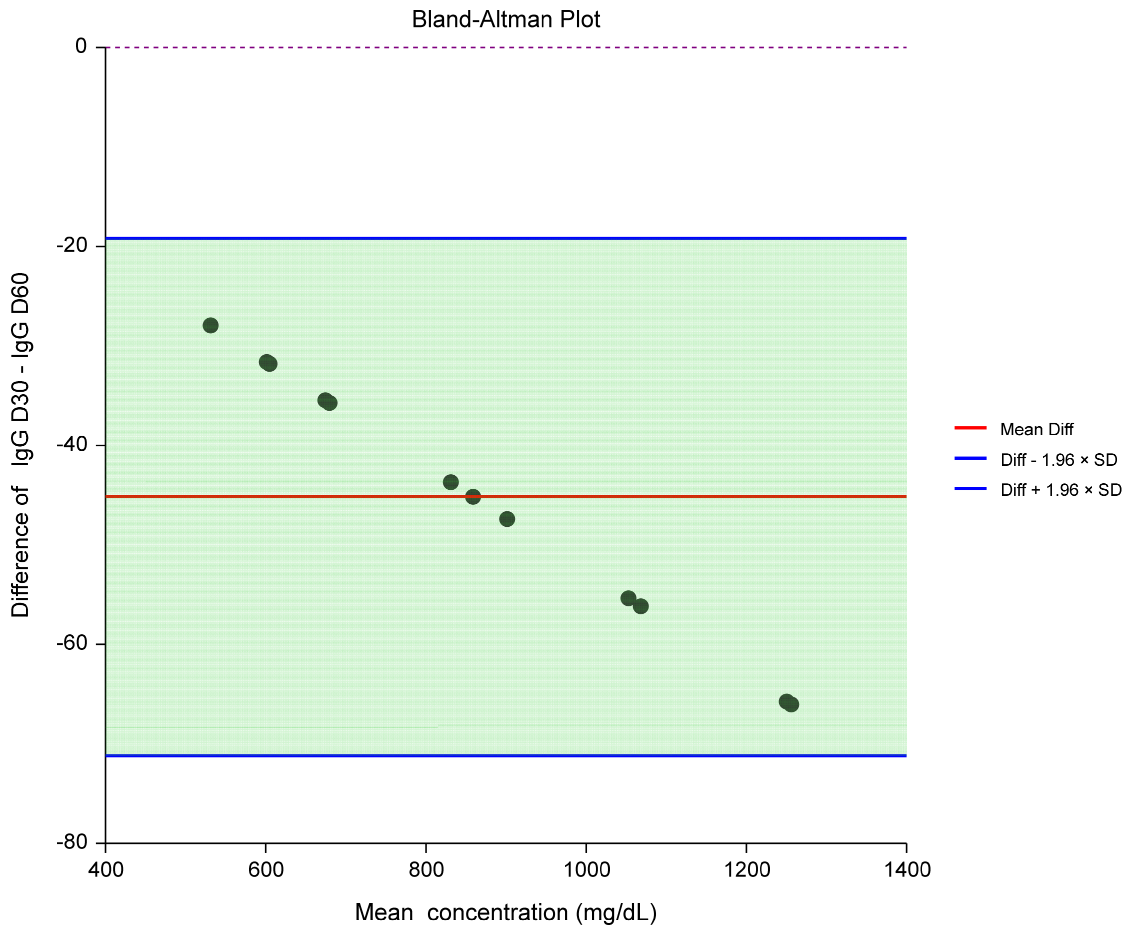

Figure S7. Bland-Altman plot of concentration of IgG for Day 30 and Day 60 in placebo crossed over to test group.

Table S7. Bias and Limits of Agreement of concentration of IgG for Day 30 and Day 60 in placebo crossed over to test group.

Parameter	Count	Value	Standard Deviation	95.0% LCL of Value	95.0% UCL of Value
Bias (Difference)	12	−45.18	13.26	−53.60	−36.75
Lower Limit of Agreement	12	−71.16	6.73	−85.99	−56.34
Upper Limit of Agreement	12	−19.19	6.73	−34.01	−4.36

Limits of Agreement = Difference ± 1.96 × (Standard Deviation of Difference).

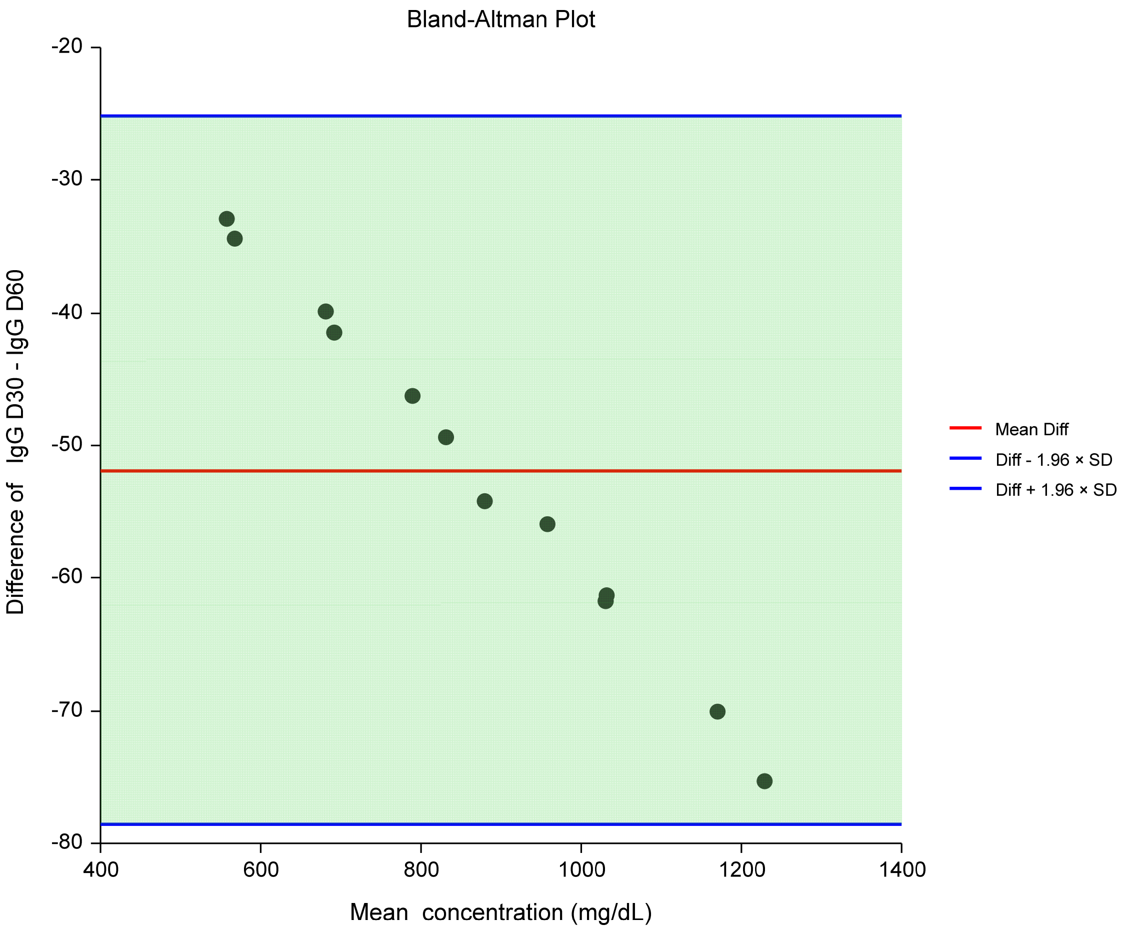

Figure S8. Bland-Altman plot of concentration of IgG for Day 30 and Day 60 in test group.

Table S8. Bias and Limits of Agreement of concentration of IgG for Day 30 and Day 60 in test group.

Parameter	Count	Value	Standard Deviation	95.0% LCL of Value	95.0% UCL of Value
Bias (Difference)	12	−51.91	13.62	−60.57	−43.25
Lower Limit of Agreement	12	−78.61	6.92	−93.84	−63.38
Upper Limit of Agreement	12	−25.21	6.92	−40.44	−9.98

Limits of Agreement = Difference ± 1.96 × (Standard Deviation of Difference).

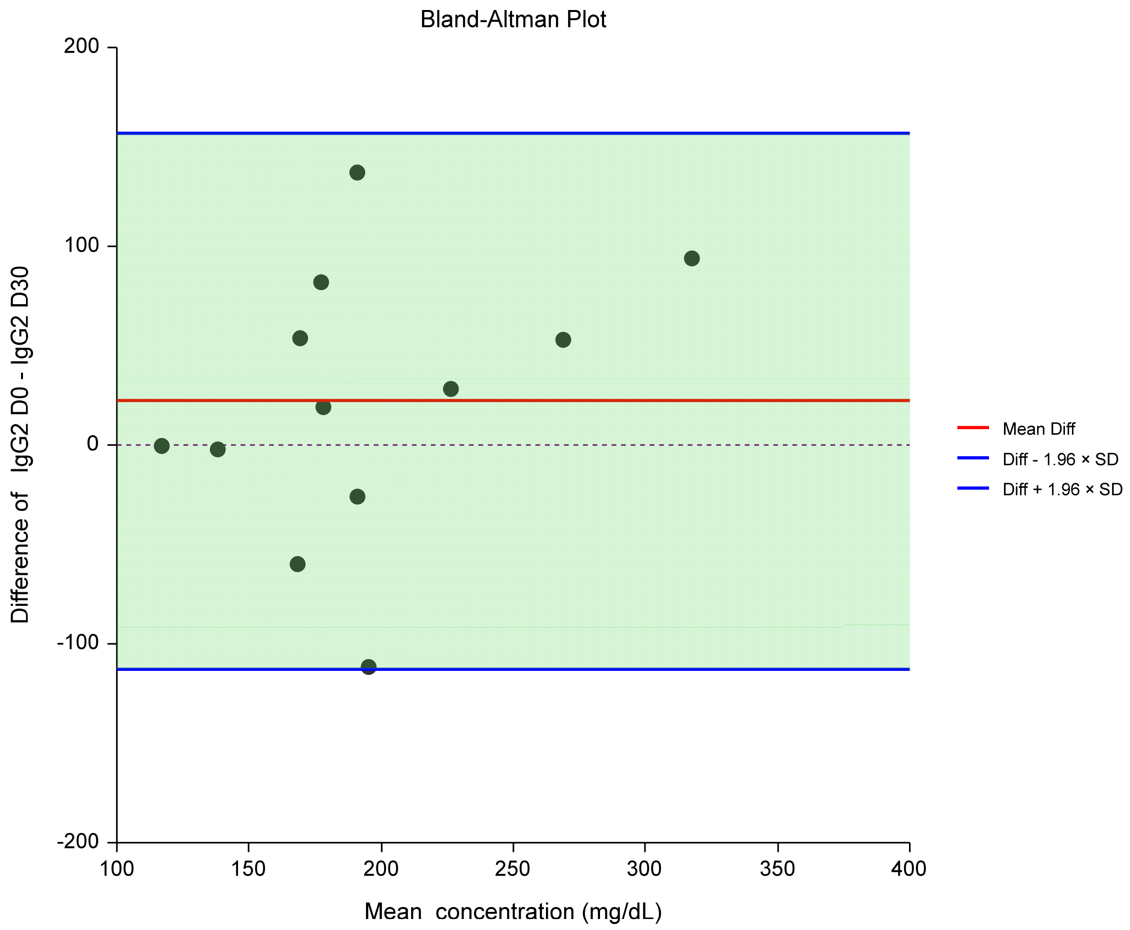

Figure S9. Bland-Altman plot of concentration of IgG2 for Day 0 and Day 30 in placebo group.

Table S9. Bias and Limits of Agreement of concentration of IgG2 for Day 0 and Day 30 in placebo group.

Parameter	Count	Value	Standard Deviation	95.0% LCL of Value	95.0% UCL of Value
Bias (Difference)	12	22.23	68.74	−21.44	65.91
Lower Limit of Agreement	12	−112.49	34.91	−189.32	−35.65
Upper Limit of Agreement	12	156.96	34.91	80.12	233.79

Limits of Agreement = Difference ± 1.96 × (Standard Deviation of Difference).

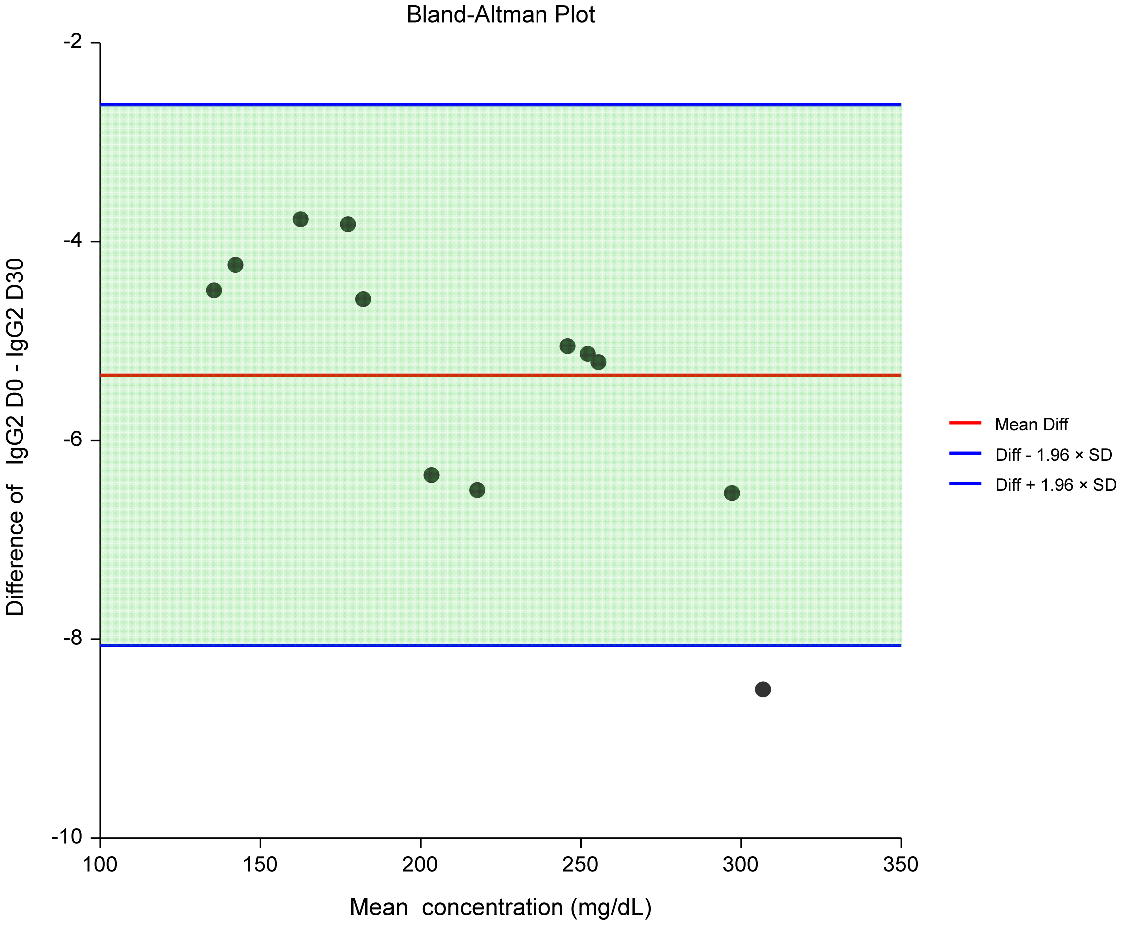

Figure S10. Bland-Altman plot of concentration of IgG2 for Day 0 and Day 30 in test group.

Table S10. Bias and Limits of Agreement of concentration of IgG2 for Day 0 and Day 30 in test group.

Parameter	Count	Value	Standard Deviation	95.0% LCL of Value	95.0% UCL of Value
Bias (Difference)	12	−5.35	1.39	−6.23	−4.47
Lower Limit of Agreement	12	−8.07	0.71	−9.62	−6.52
Upper Limit of Agreement	12	−2.63	0.71	−4.18	−1.07

Limits of Agreement = Difference ± 1.96 × (Standard Deviation of Difference).

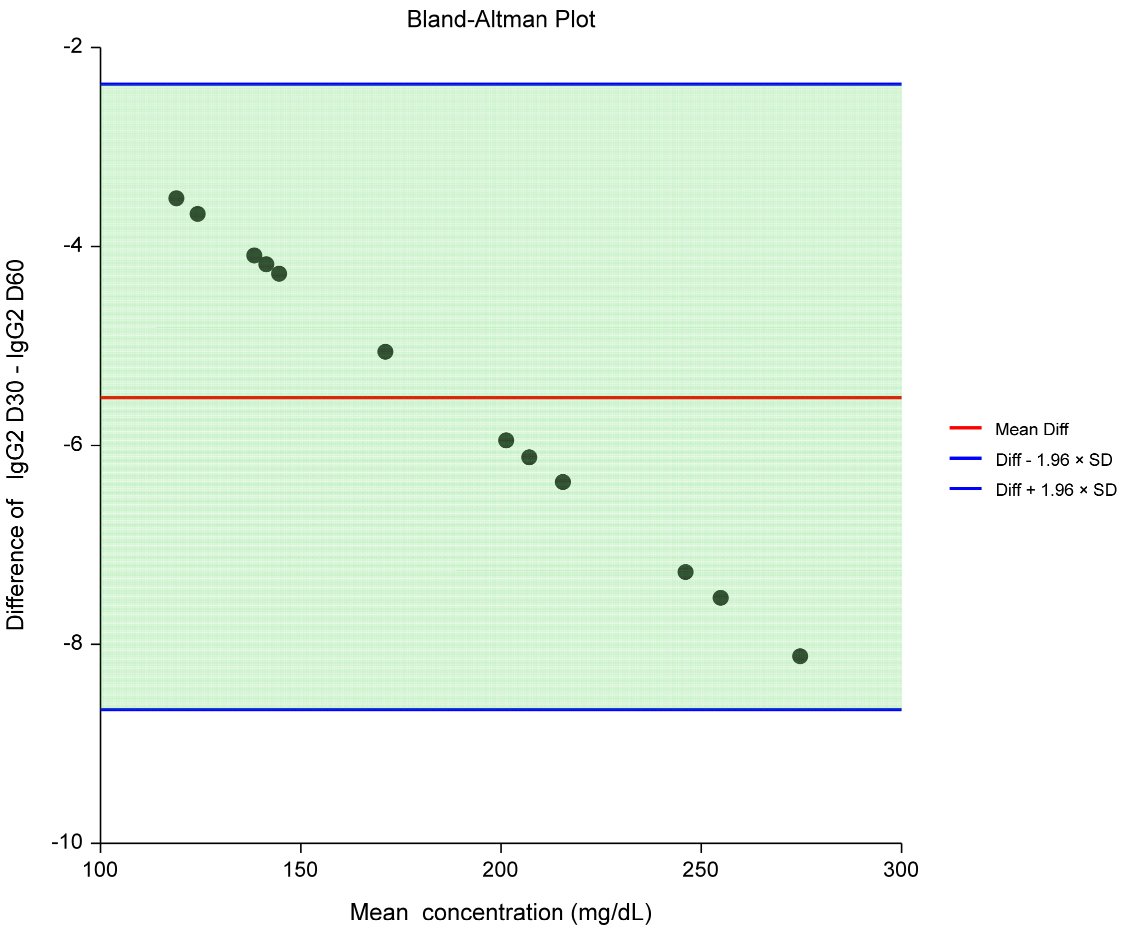

Figure S11. Bland-Altman plot of concentration of IgG2 for Day 30 and Day 60 in placebo crossed over to test group.

Table S11. Bias and Limits of Agreement of concentration of IgG2 for Day 30 and Day 60 in placebo crossed over to test group.

Parameter	Count	Value	Standard Deviation	95.0% LCL of Value	95.0% UCL of Value
Bias (Difference)	12	−5.51	1.60	−6.53	−4.50
Lower Limit of Agreement	12	−8.65	0.81	−10.44	−6.86
Upper Limit of Agreement	12	−2.37	0.81	−4.16	−0.59

Limits of Agreement = Difference ± 1.96 × (Standard Deviation of Difference).

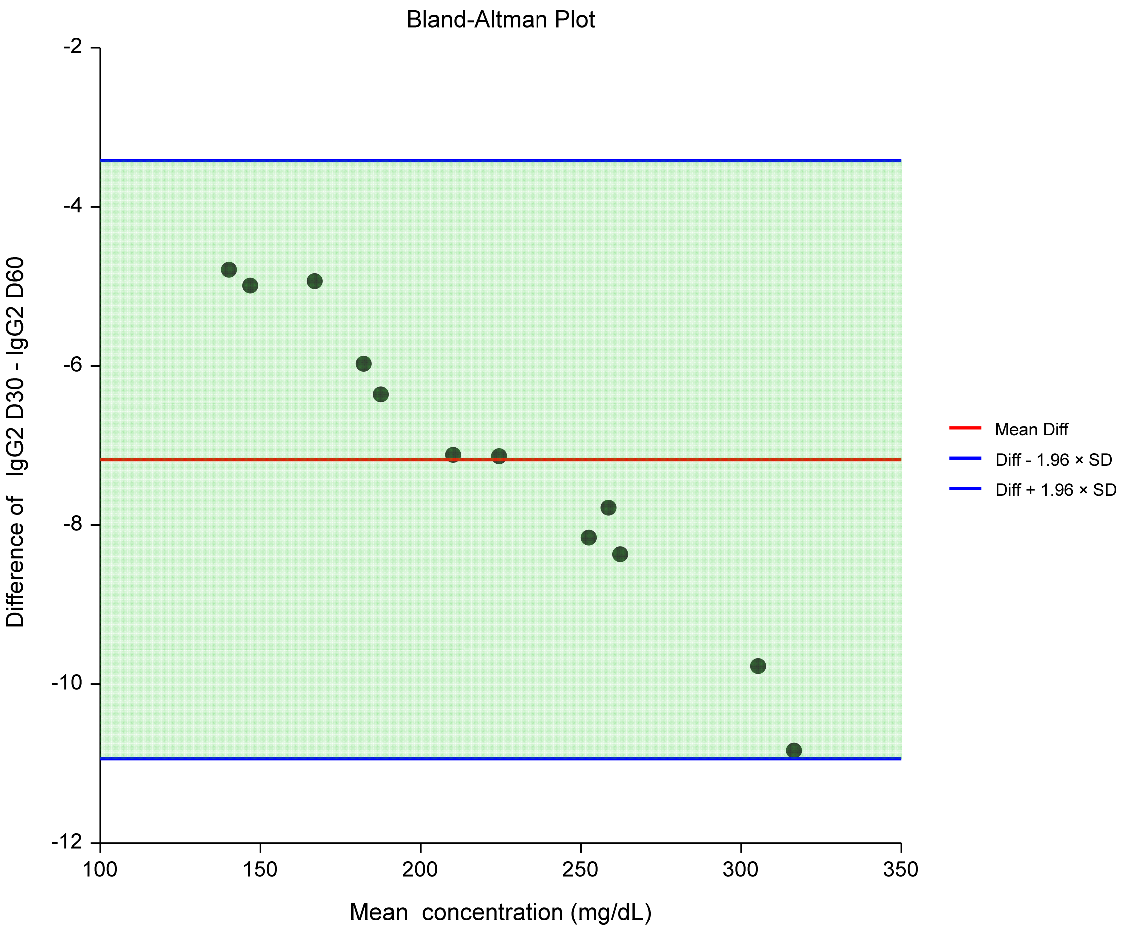

Figure S12. Bland-Altman plot of concentration of IgG2 for Day 30 and Day 60 in test group.

Table S12. Bias and Limits of Agreement of concentration of IgG2 for Day 30 and Day 60 in test group.

**Parameter**	**Count**	**Value**	**Standard Deviation**	**95.0% LCL of Value**	**95.0% UCL of Value**
Bias (Difference)	12	−7.18	1.92	−8.40	−5.97
Lower Limit of Agreement	12	−10.95	0.97	−13.09	−8.80
Upper Limit of Agreement	12	−3.42	0.97	−5.57	−1.28

Limits of Agreement = Difference ± 1.96 × (Standard Deviation of Difference).

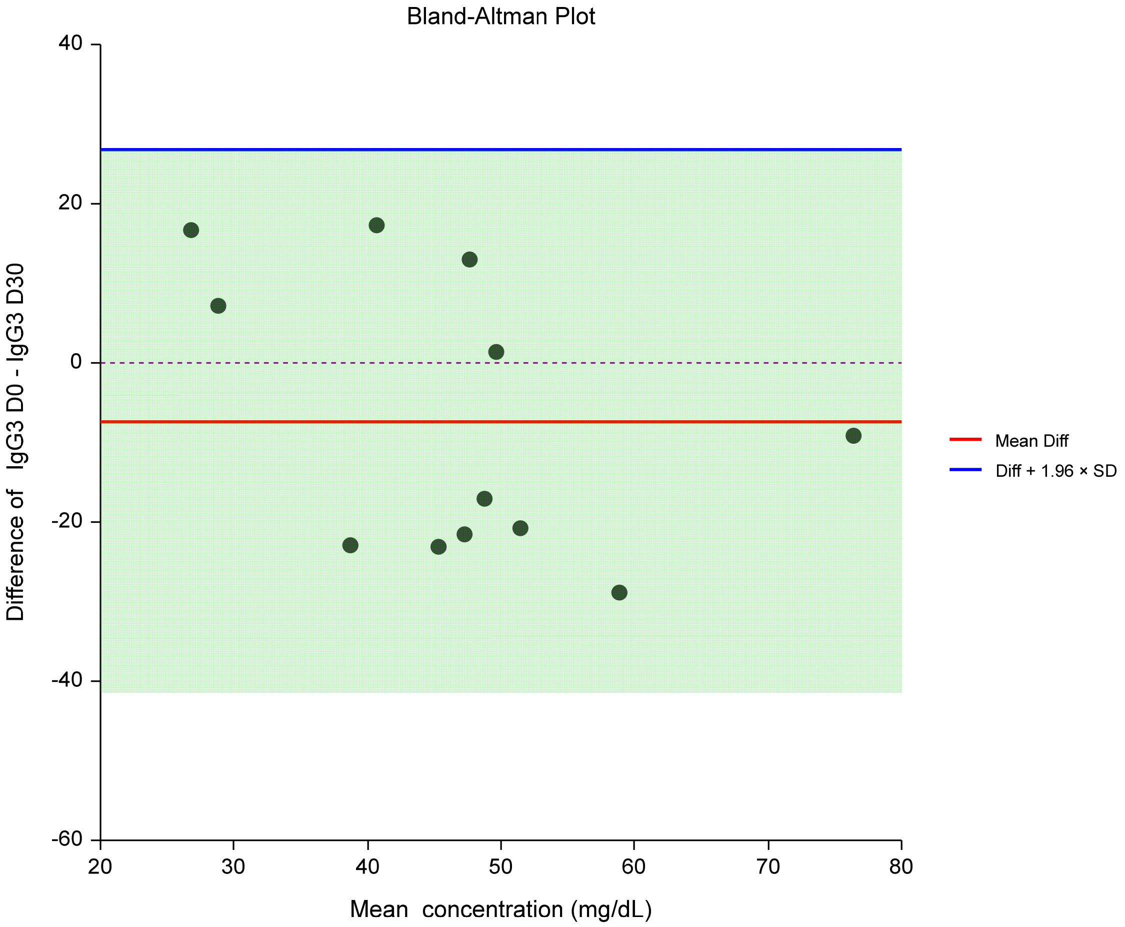

Figure S13. Bland-Altman plot of concentration of IgG3 for Day 0 and Day 30 in placebo group.

Table S13. Bias and Limits of Agreement of concentration of IgG3 for Day 0 and Day 30 in placebo group.

Parameter	Count	Value	Standard Deviation	95.0% LCL of Value	95.0% UCL of Value
Bias (Difference)	12	−7.33	17.37	−18.37	3.71
Lower Limit of Agreement	12	−41.37	8.82	−60.79	−21.96
Upper Limit of Agreement	12	26.71	8.82	7.30	46.13

Limits of Agreement = Difference ± 1.96 × (Standard Deviation of Difference).

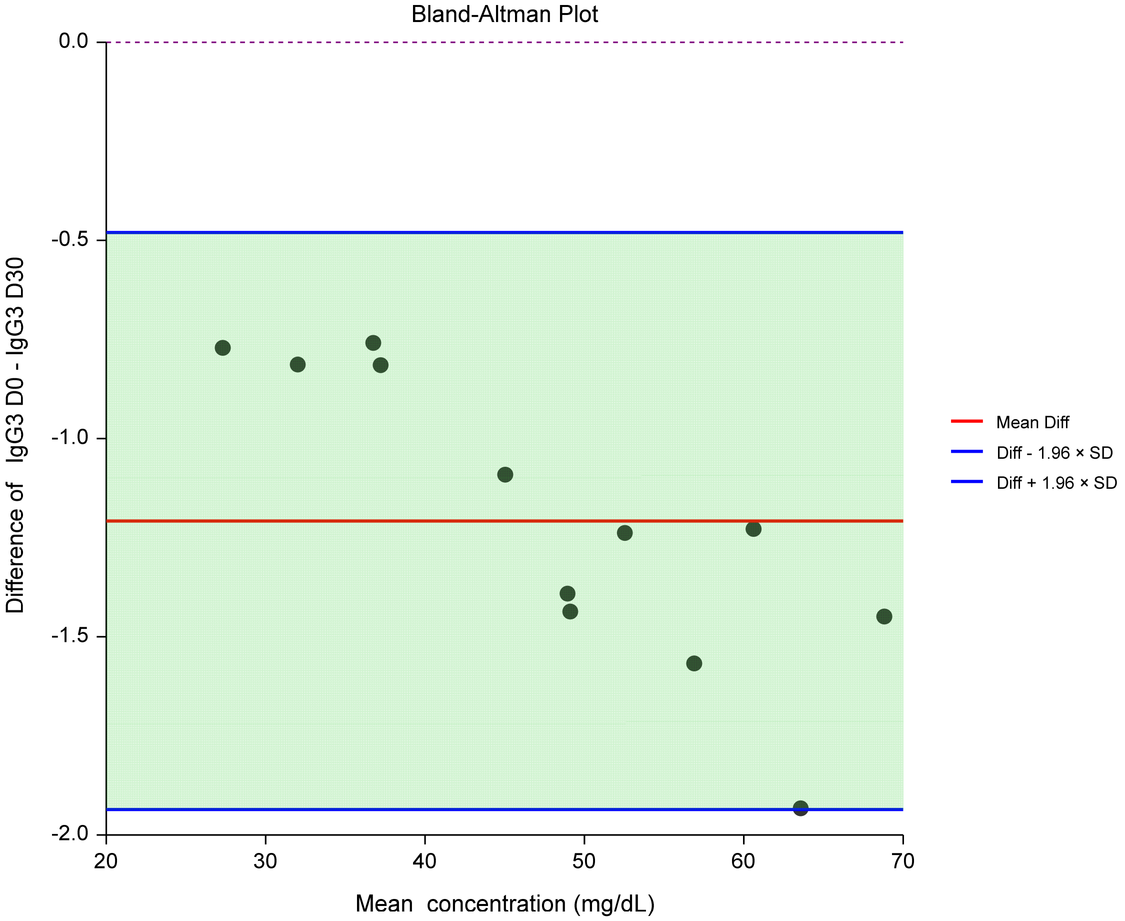

Figure S14. Bland-Altman plot of concentration of IgG3 for Day 0 and Day 30 in test group.

Table S14. Bias and Limits of Agreement of concentration of IgG3 for Day 0 and Day 30 in test group.

Parameter	Count	Value	Standard Deviation	95.0% LCL of Value	95.0% UCL of Value
Bias (Difference)	12	−1.21	0.37	−1.44	−0.97
Lower Limit of Agreement	12	−1.94	0.19	−2.35	−1.52
Upper Limit of Agreement	12	−0.48	0.19	−0.89	−0.06

Limits of Agreement = Difference ± 1.96 × (Standard Deviation of Difference).

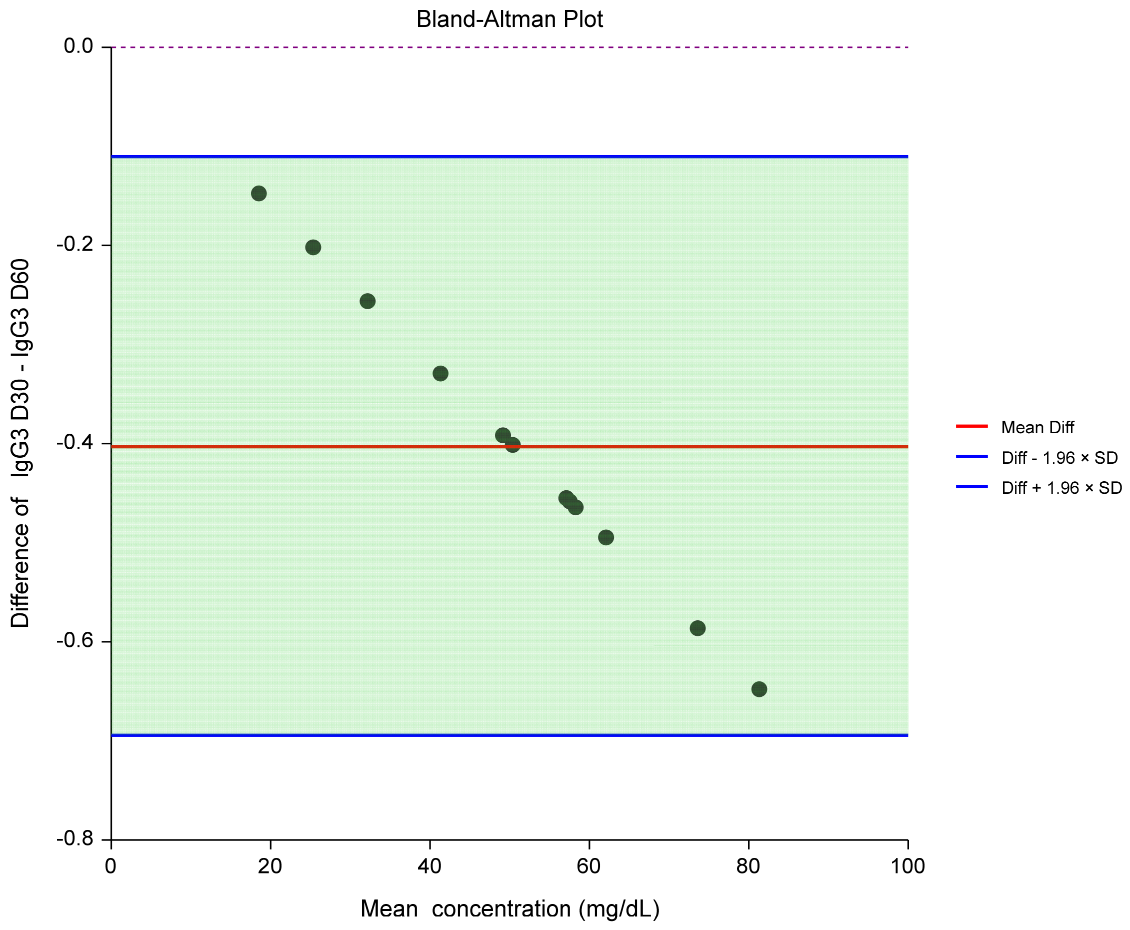

Figure S15. Bland-Altman plot of concentration of IgG3 for Day 30 and Day 60 in placebo crossed over to test group.

Table S15. Bias and Limits of Agreement of concentration of IgG3 for Day 30 and Day 60 in placebo crossed over to test group.

Parameter	Count	Value	Standard Deviation	95.0% LCL of Value	95.0% UCL of Value
Bias (Difference)	12	−0.40	0.15	−0.50	−0.31
Lower Limit of Agreement	12	−0.69	0.08	−0.86	−0.53
Upper Limit of Agreement	12	−0.11	0.08	−0.28	0.06

Limits of Agreement = Difference ± 1.96 × (Standard Deviation of Difference).

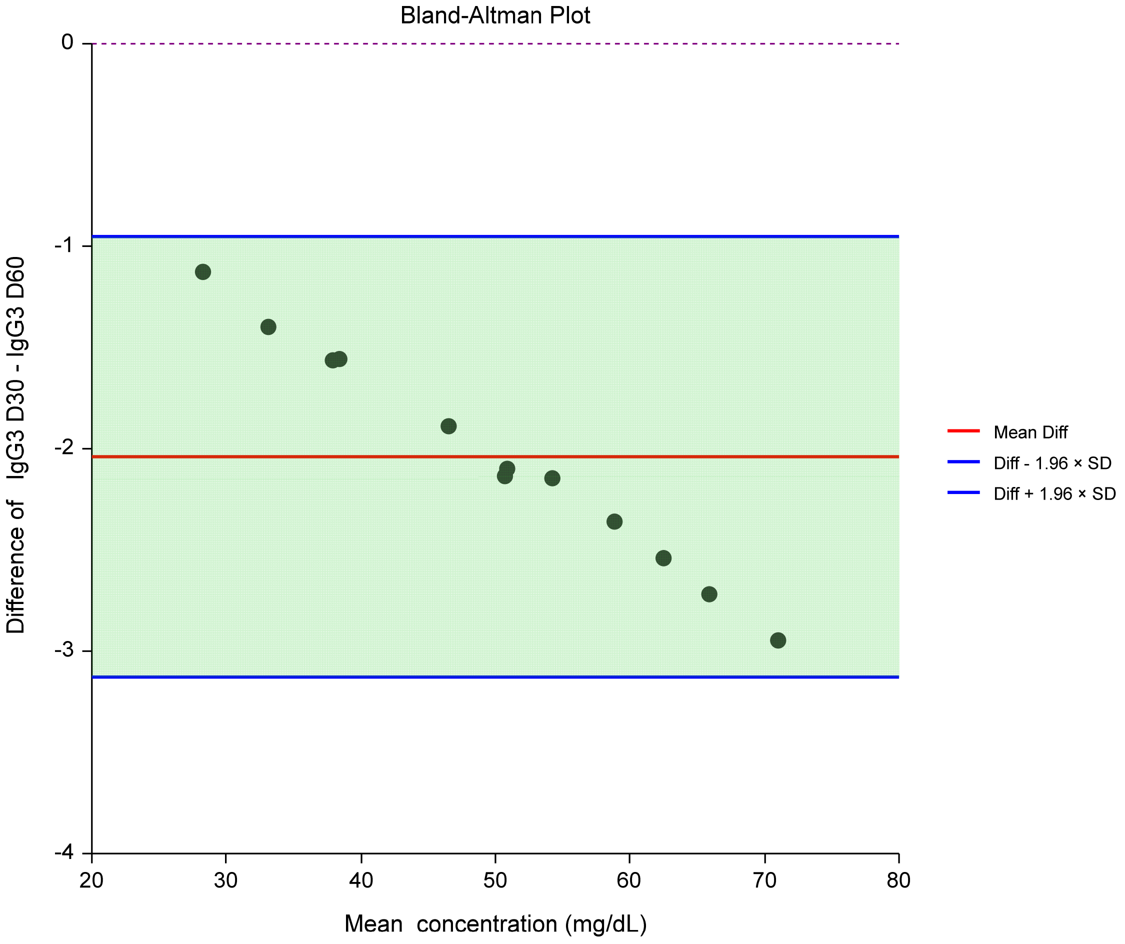

Figure S16. Bland-Altman plot of concentration of IgG3 for Day 30 and Day 60 in test group.

Table S16. Bias and Limits of Agreement of concentration of IgG3 for Day 30 and Day 60 in test group.

Parameter	Count	Value	Standard Deviation	95.0% LCL of Value	95.0% UCL of Value
Bias (Difference)	12	−2.04	0.56	−2.39	−1.69
Lower Limit of Agreement	12	−3.13	0.28	−3.75	−2.51
Upper Limit of Agreement	12	−0.95	0.28	−1.57	−0.33

Limits of Agreement = Difference ± 1.96 × (Standard Deviation of Difference).

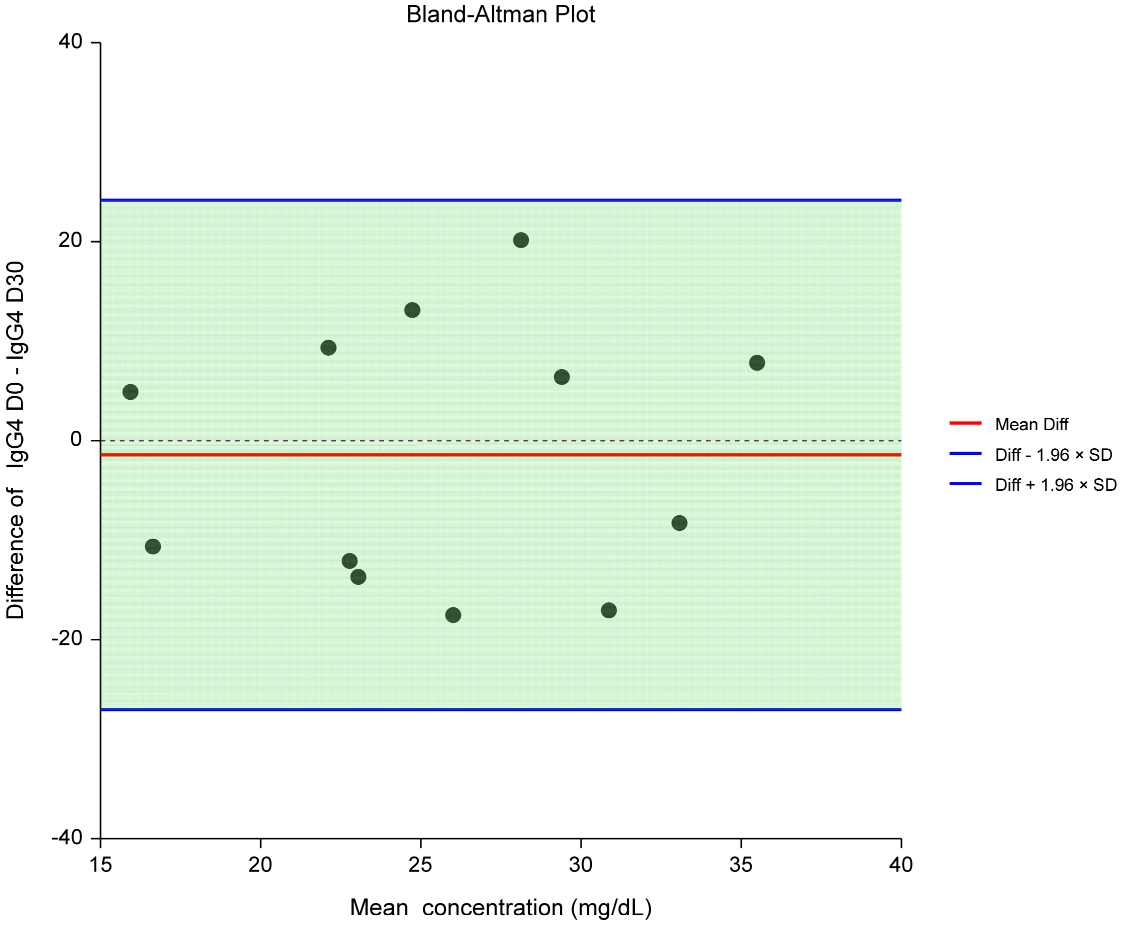

Figure S17. Bland-Altman plot of concentration of IgG4 for Day 0 and Day 30 in placebo group.

Table S17. Bias and Limits of Agreement of concentration of IgG4 for Day 0 and Day 30 in placebo group.

Parameter	Count	Value	Standard Deviation	95.0% LCL of Value	95.0% UCL of Value
Bias (Difference)	12	−1.47	13.07	−9.78	6.83
Lower Limit of Agreement	12	−27.09	6.64	−41.71	−12.48
Upper Limit of Agreement	12	24.15	6.64	9.54	38.76

Limits of Agreement = Difference ± 1.96 × (Standard Deviation of Difference).

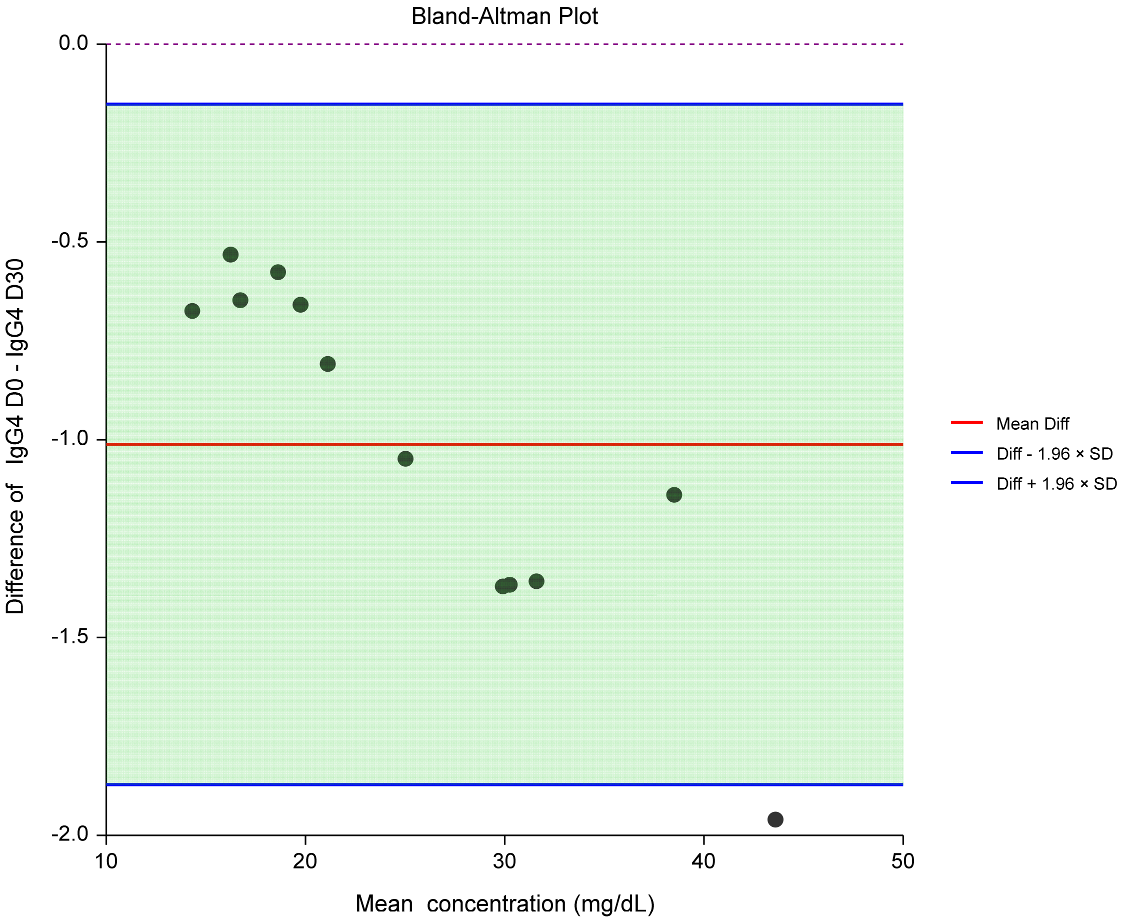

Figure S18. Bland-Altman plot of concentration of IgG4 for Day 0 and Day 30 in test group.

Table S18. Bias and Limits of Agreement of concentration of IgG4 for Day 0 and Day 30 in test group.

Parameter	Count	Value	Standard Deviation	95.0% LCL of Value	95.0% UCL of Value
Bias (Difference)	12	−1.01	0.44	−1.29	−0.73
Lower Limit of Agreement	12	−1.87	0.22	−2.36	−1.38
Upper Limit of Agreement	12	−0.15	0.22	−0.64	0.34

Limits of Agreement = Difference ± 1.96 × (Standard Deviation of Difference).

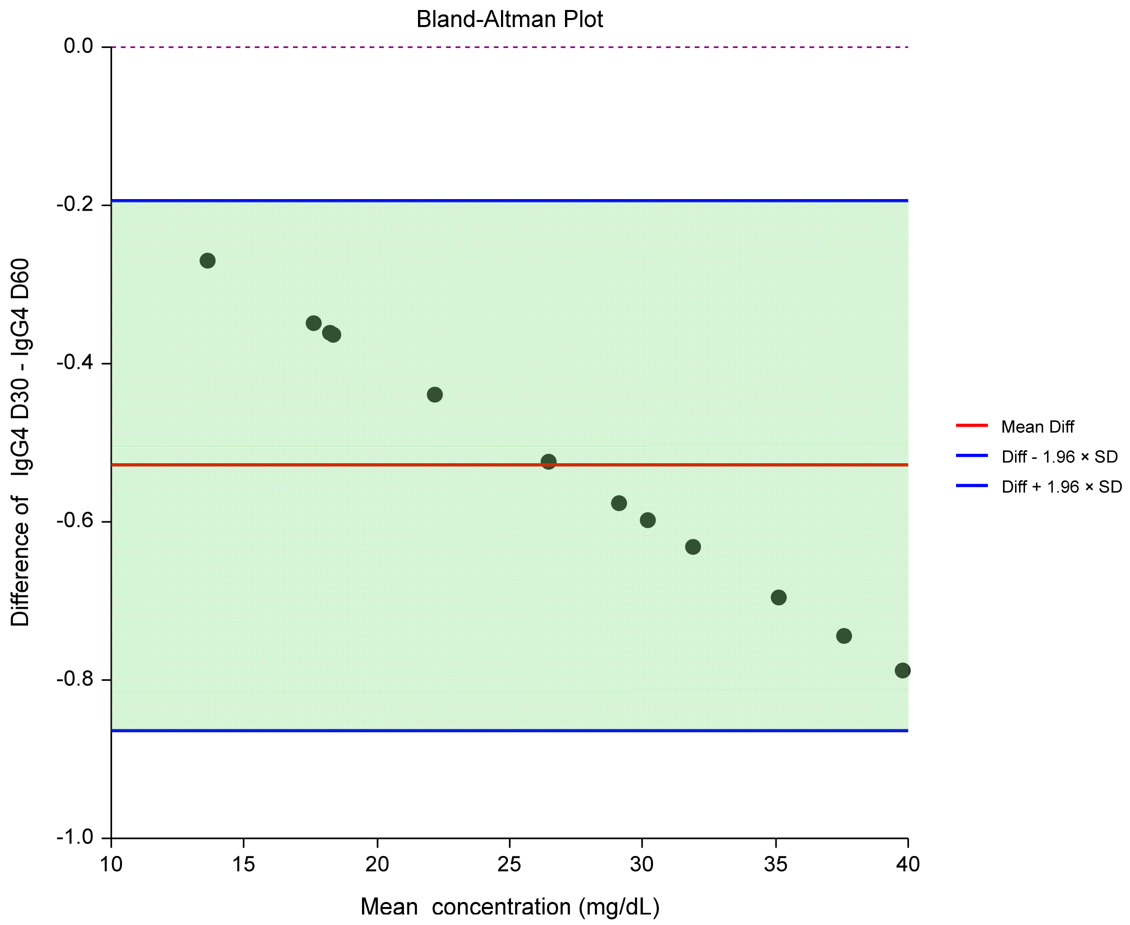

Figure S19. Bland-Altman plot of concentration of IgG4 for Day 30 and Day 60 in placebo crossed over to test group.

Table S19. Bias and Limits of Agreement of concentration of IgG4 for Day 30 and Day 60 in placebo crossed over to test group.

Parameter	Count	Value	Standard Deviation	95.0% LCL of Value	95.0% UCL of Value
Bias (Difference)	12	−0.53	0.17	−0.64	−0.42
Lower Limit of Agreement	12	−0.86	0.09	−1.05	−0.67
Upper Limit of Agreement	12	−0.19	0.09	−0.38	0.00

Limits of Agreement = Difference ± 1.96 × (Standard Deviation of Difference).

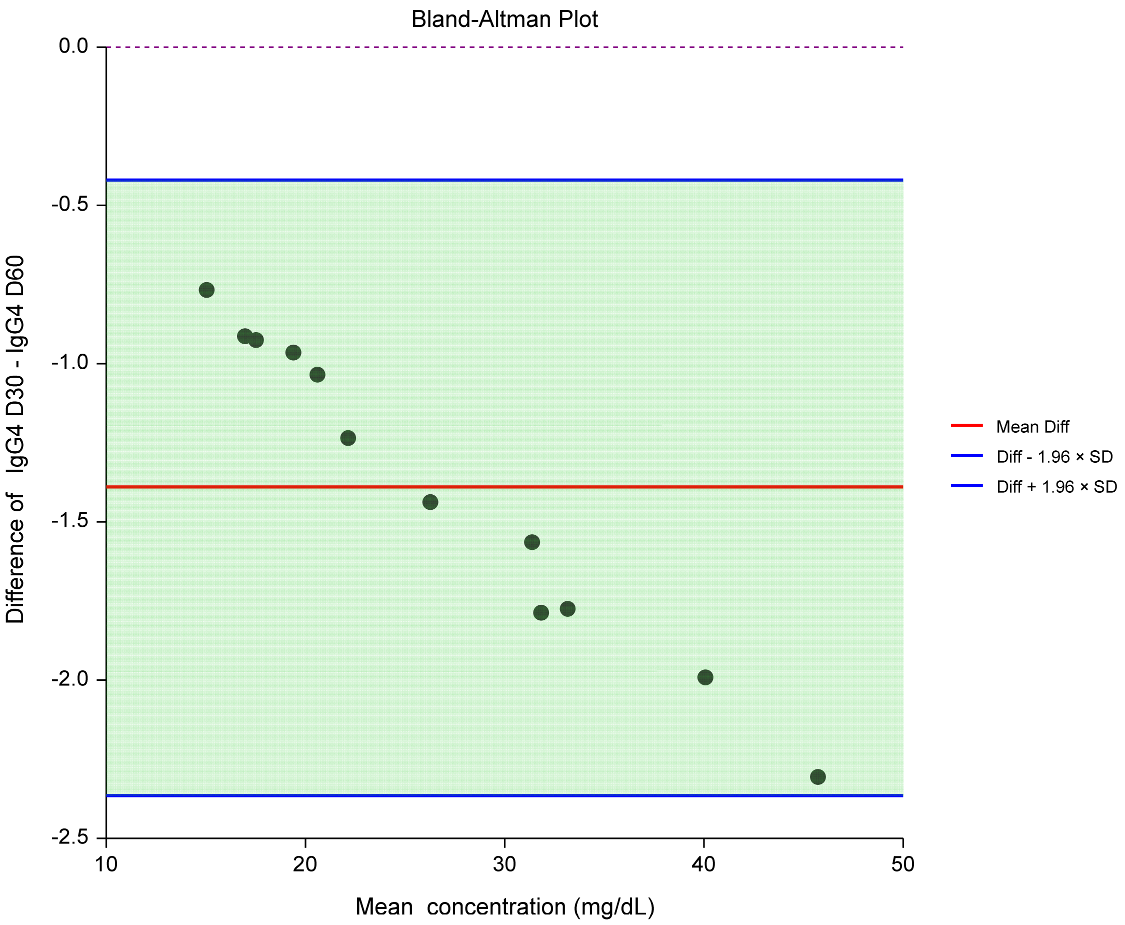

Figure S20. Bland-Altman plot of concentration of IgG4 for Day 30 and Day 60 in test group.

Table S20. Bias and Limits of Agreement of concentration of IgG4 for Day 30 and Day 60 in test group.

Parameter	Count	Value	Standard Deviation	95.0% LCL of Value	95.0% UCL of Value
Bias (Difference)	12	−1.39	0.50	−1.71	−1.08
Lower Limit of Agreement	12	−2.36	0.25	−2.92	−1.81
Upper Limit of Agreement	12	−0.42	0.25	−0.97	0.13

Limits of Agreement = Difference ± 1.96 × (Standard Deviation of Difference).

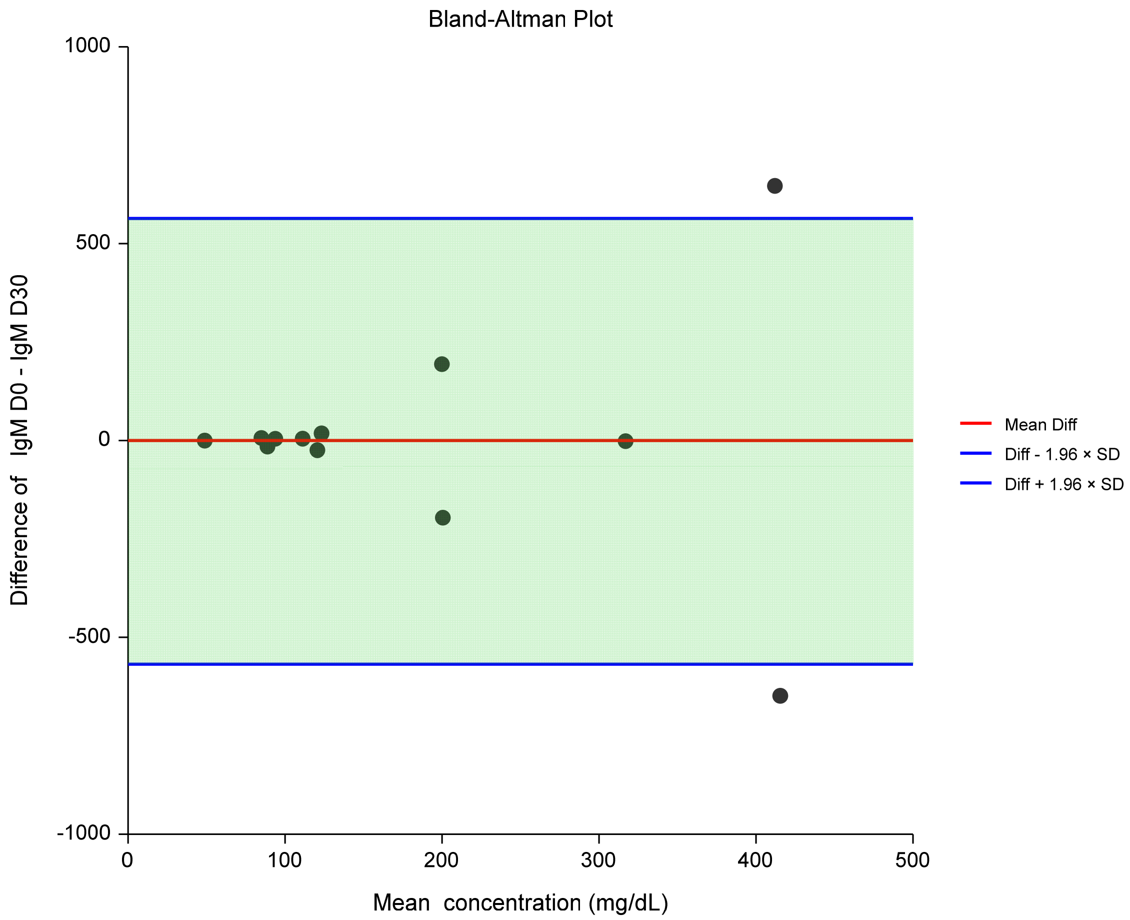

Figure S21. Bland-Altman plot of concentration of IgM for Day 0 and Day 30 in placebo group.

Table S21. Bias and Limits of Agreement of concentration of IgM for Day 0 and Day 30 in placebo group.

Parameter	Count	Value	Standard Deviation	95.0% LCL of Value	95.0% UCL of Value
Bias (Difference)	12	−1.03	288.58	−184.39	182.33
Lower Limit of Agreement	12	−566.65	146.57	−889.25	−244.06
Upper Limit of Agreement	12	564.59	146.57	242.00	887.19

Limits of Agreement = Difference ± 1.96 × (Standard Deviation of Difference).

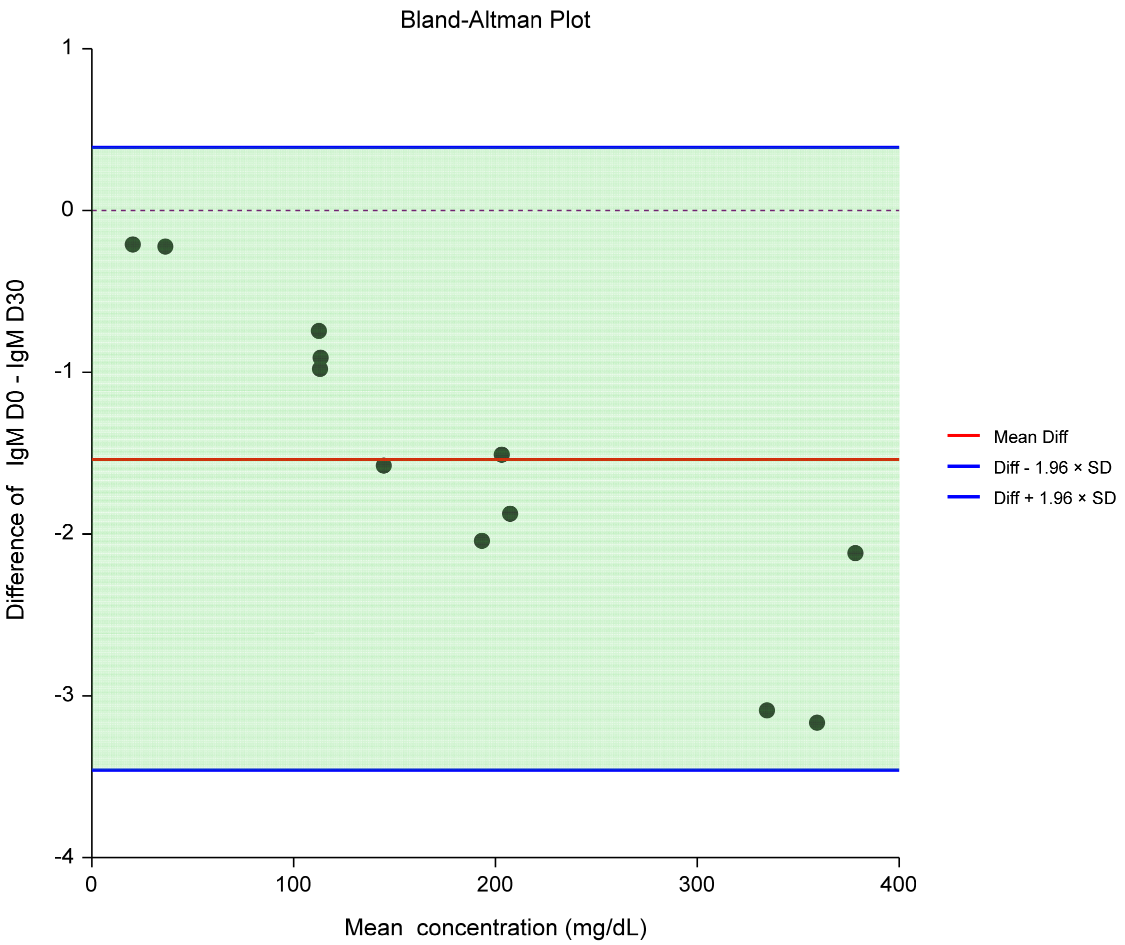

Figure S22. Bland-Altman plot of concentration of IgM for Day 0 and Day 30 in test group.

Table S22. Bias and Limits of Agreement of concentration of IgM for Day 0 and Day 30 in test group.

Parameter	Count	Value	Standard Deviation	95.0% LCL of Value	95.0% UCL of Value
Bias (Difference)	12	−1.54	0.98	−2.16	−0.91
Lower Limit of Agreement	12	−3.46	0.50	−4.56	−2.36
Upper Limit of Agreement	12	0.39	0.50	−0.71	1.48

Limits of Agreement = Difference ± 1.96 × (Standard Deviation of Difference).

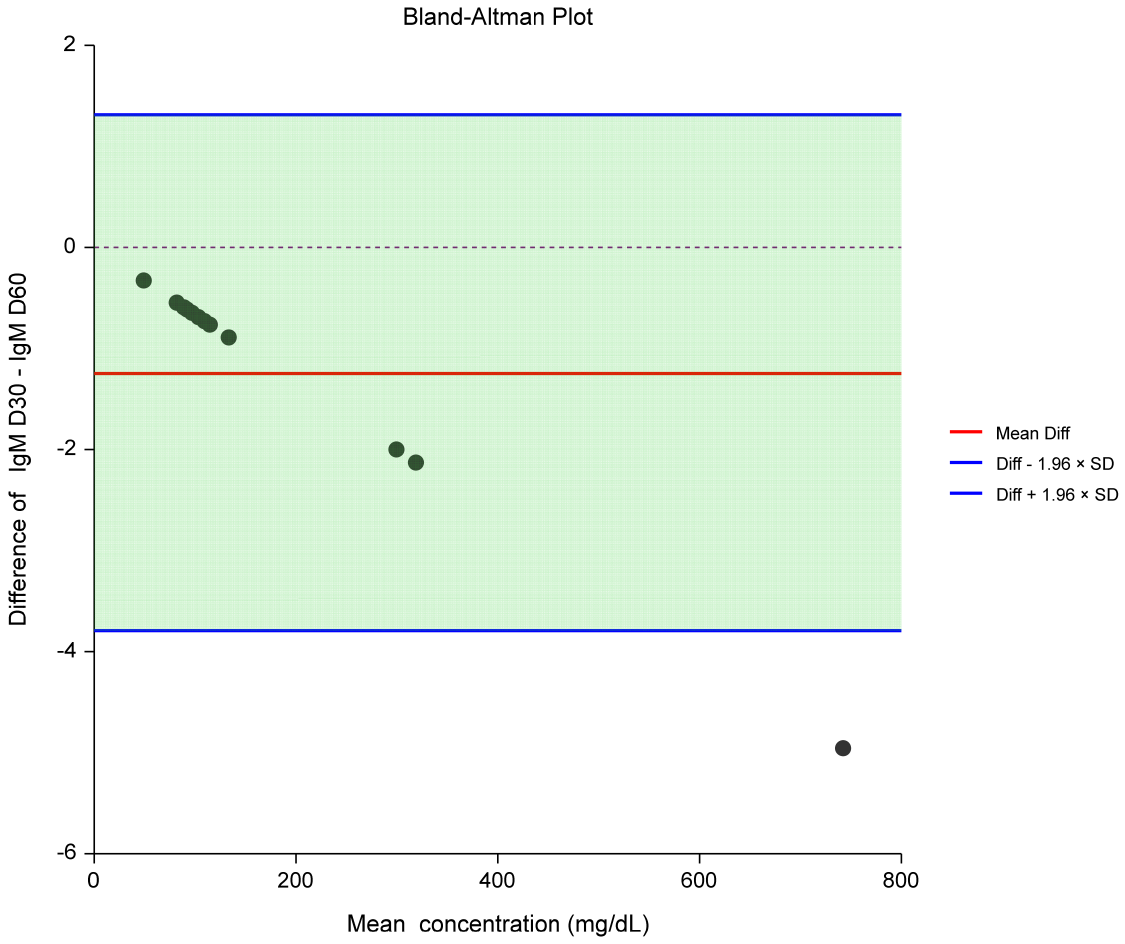

Figure S23. Bland-Altman plot of concentration of IgM for Day 30 and Day 60 in placebo crossed over to test group.

Table S23. Bias and Limits of Agreement of concentration of IgM for Day 30 and Day 60 in placebo crossed over to test group.

Parameter	Count	Value	Standard Deviation	95.0% LCL of Value	95.0% UCL of Value
Bias (Difference)	12	−1.24	1.30	−2.07	−0.42
Lower Limit of Agreement	12	−3.79	0.66	−5.24	−2.33
Upper Limit of Agreement	12	1.31	0.66	−0.15	2.76

Limits of Agreement = Difference ± 1.96 × (Standard Deviation of Difference).

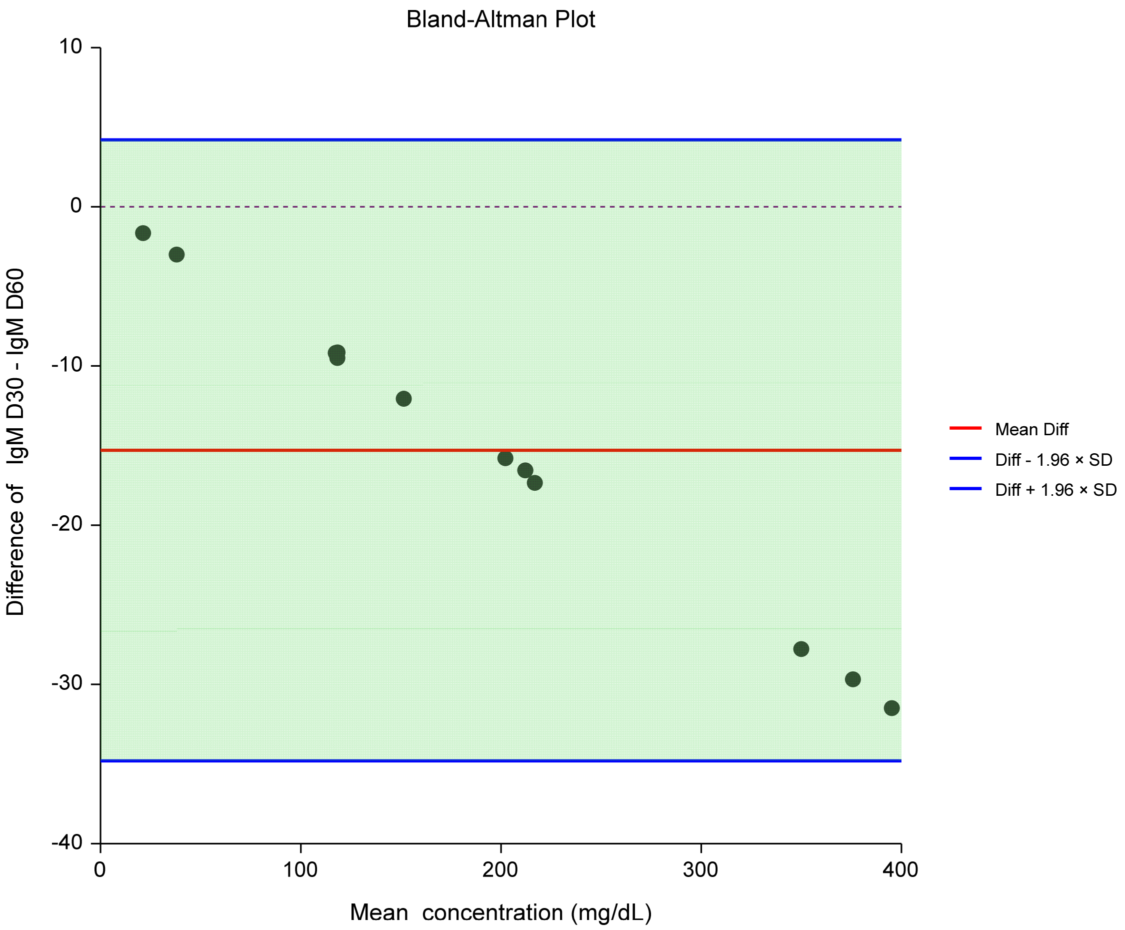

Figure S24. Bland-Altman plot of concentration of IgM for Day 30 and Day 60 in test group.

Table S24. Bias and Limits of Agreement of concentration of IgM for Day 30 and Day 60 in test group.

Parameter	Count	Value	Standard Deviation	95.0% LCL of Value	95.0% UCL of Value
Bias (Difference)	12	−15.27	9.94	−21.58	−8.95
Lower Limit of Agreement	12	−34.75	5.05	−45.87	−23.64
Upper Limit of Agreement	12	4.22	5.05	−6.89	15.34

Limits of Agreement = Difference ± 1.96 × (Standard Deviation of Difference).
