# Supplementary material for: Immunomodulatory Effect of Withania somnifera (Ashwagandha) Extract—A Randomized, Double-Blind, Placebo Controlled Trial with an Open Label Extension on Healthy Participants
Source: J Clin Med. 2021 Aug 18;10(16):3644. doi: 10.3390/jcm10163644 (PMC8397213; doi:10.3390/jcm10163644)
Supplement: Supplementary file 1 [file jcm-10-03644-s001.zip › supplementary file 3 - final.pdf]

Supplementary file 3. Bland-Altman Analysis of Cytokines

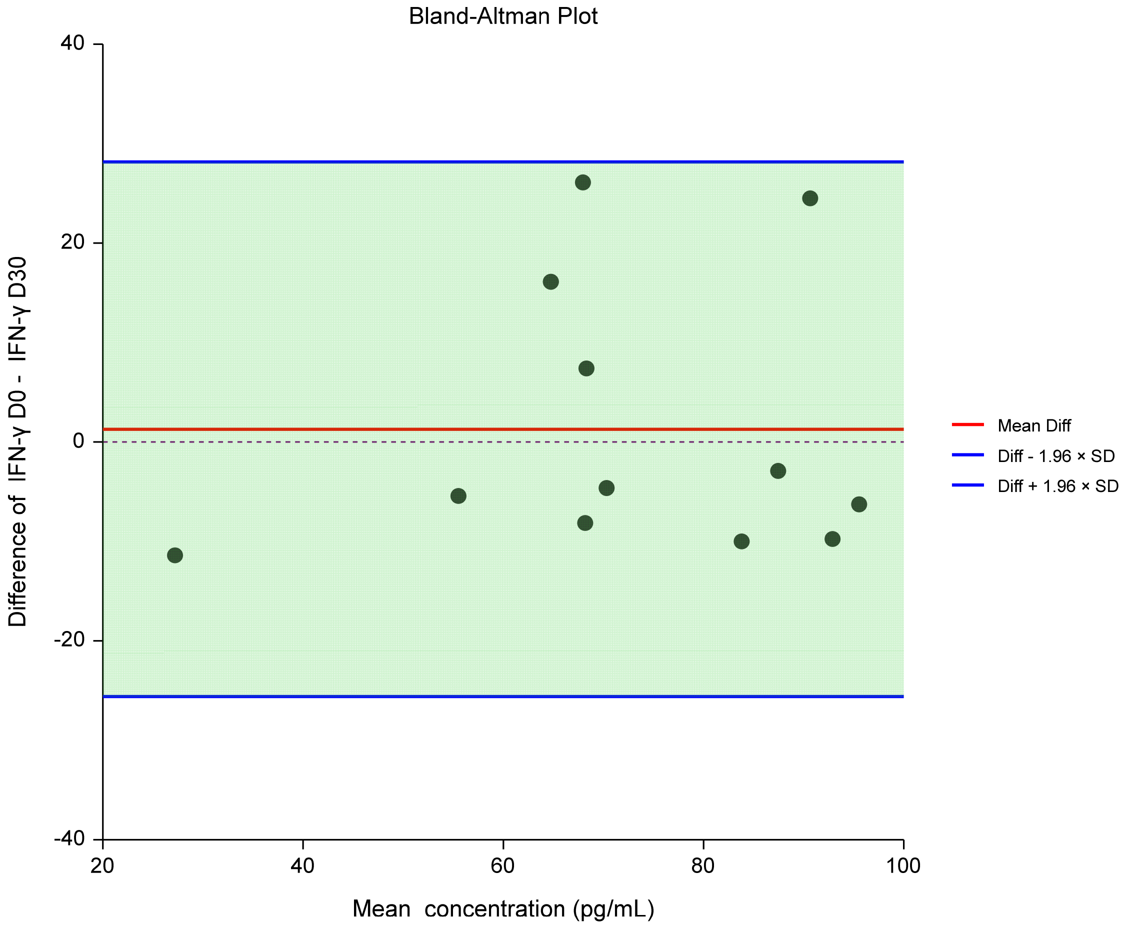

Figure S1. Bland-Altman plot of Concentration of IFN-γ for Day 0 and Day 30 in placebo group.

Table S1. Bias and Limits of Agreement of Concentration of IFN-γ for Day 0 and Day 30 in placebo group.

Parameter	Count	Value	Standard Deviation	95.0% LCL of Value	95.0% UCL of Value
Bias (Difference)	12	1.30	13.70	−7.41	10.00
Lower Limit of Agreement	12	−25.56	6.96	−40.88	−10.24
Upper Limit of Agreement	12	28.16	6.96	12.84	43.48

Limits of Agreement = Difference ± 1.96 × (Standard Deviation of Difference).

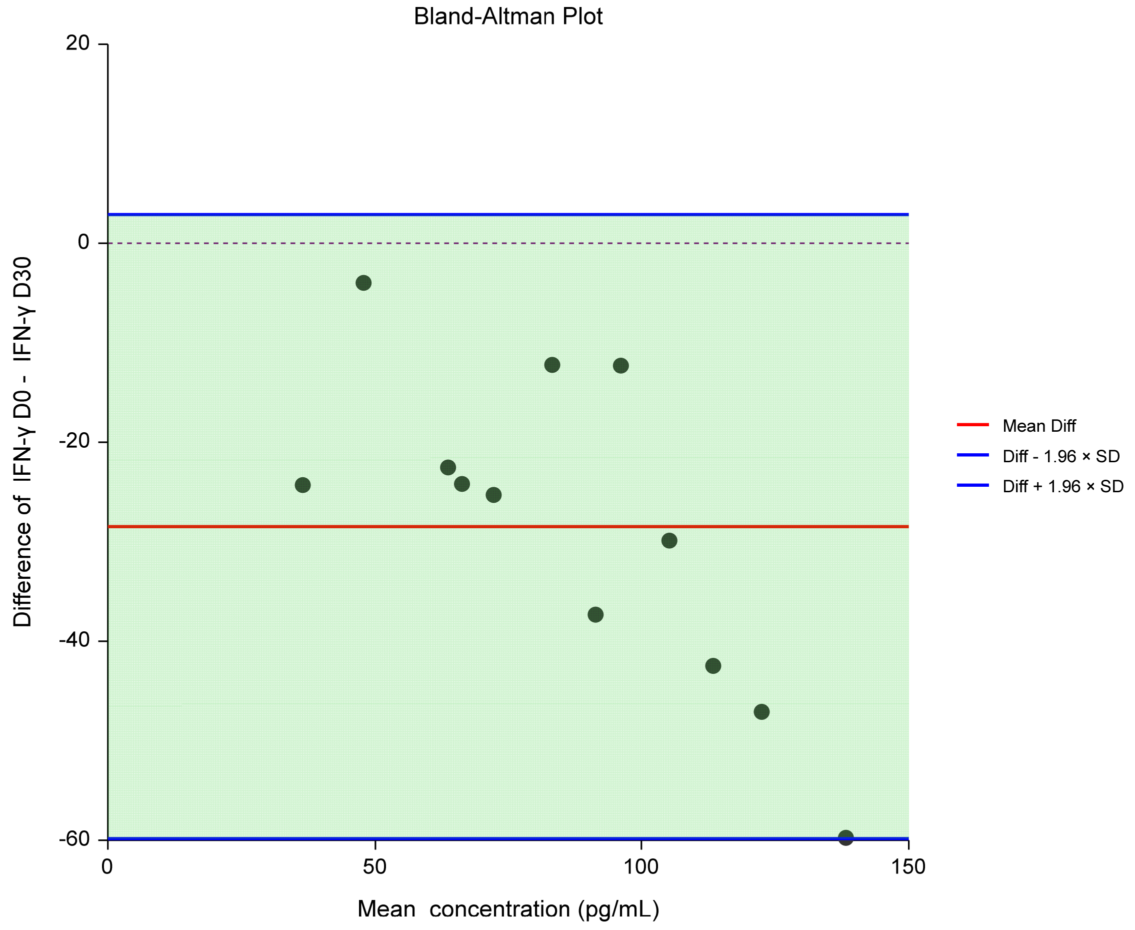

Figure S2. Bland-Altman plot of Concentration of IFN-γ for Day 0 and Day 30 in test group.

Table S2. Bias and Limits of Agreement of Concentration of IFN-γ for Day 0 and Day 30 in test group.

Parameter	Count	Value	Standard Deviation	95.0% LCL of Value	95.0% UCL of Value
Bias (Difference)	12	−28.45	15.98	−38.60	−18.30
Lower Limit of Agreement	12	−59.77	8.11	−77.63	−41.91
Upper Limit of Agreement	12	2.86	8.11	−15.00	20.72

Limits of Agreement = Difference ± 1.96 × (Standard Deviation of Difference).

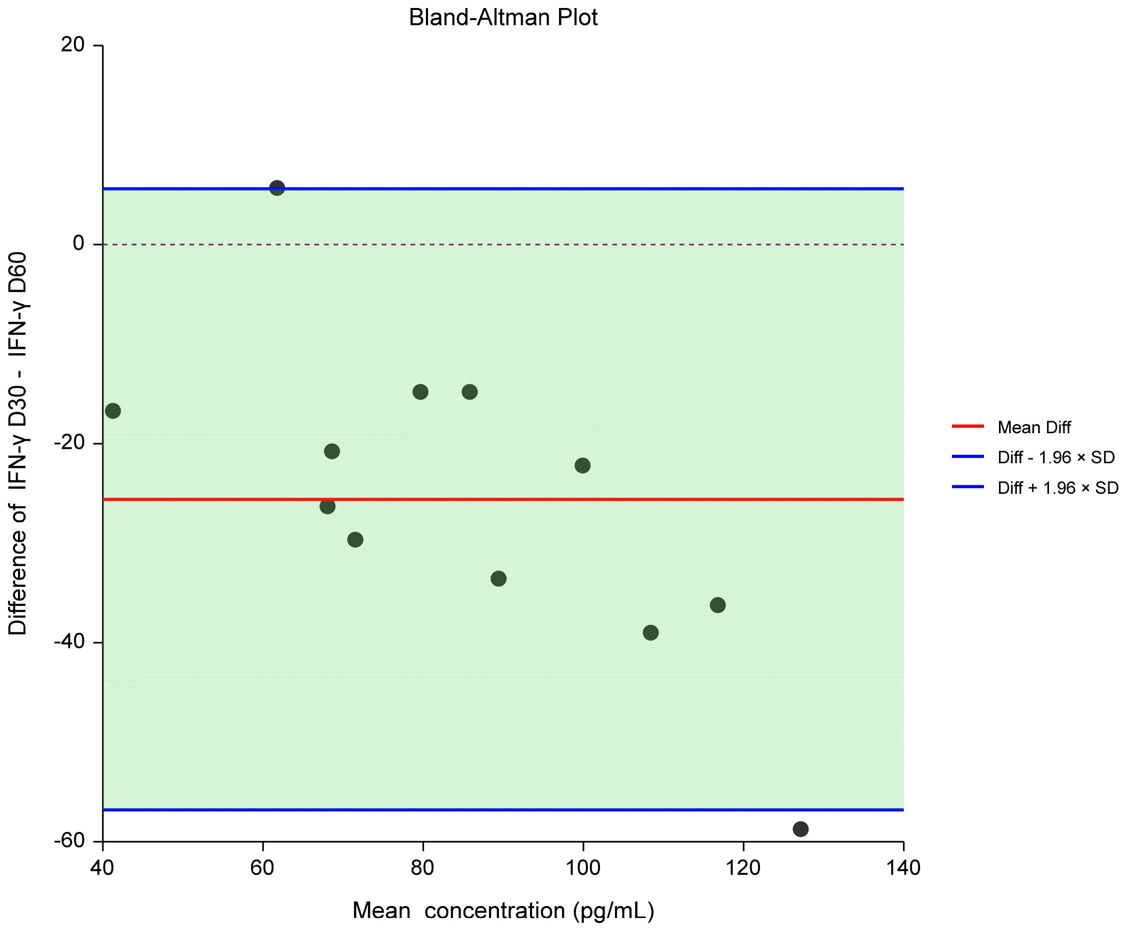

Figure S3. Bland-Altman plot of Concentration of IFN-γ for Day 30 and Day 60 in placebo crossed over to test group.

Table S3. Bias and Limits of Agreement of Concentration of IFN-γ for Day 30 and Day 60 in placebo crossed over to test group.

Parameter	Count	Value	Standard Deviation	95.0% LCL of Value	95.0% UCL of Value
Bias (Difference)	12	−25.59	15.95	−35.72	−15.46
Lower Limit of Agreement	12	−56.85	8.10	−74.68	−39.02
Upper Limit of Agreement	12	5.67	8.10	−12.16	23.50

Limits of Agreement = Difference ± 1.96 × (Standard Deviation of Difference).

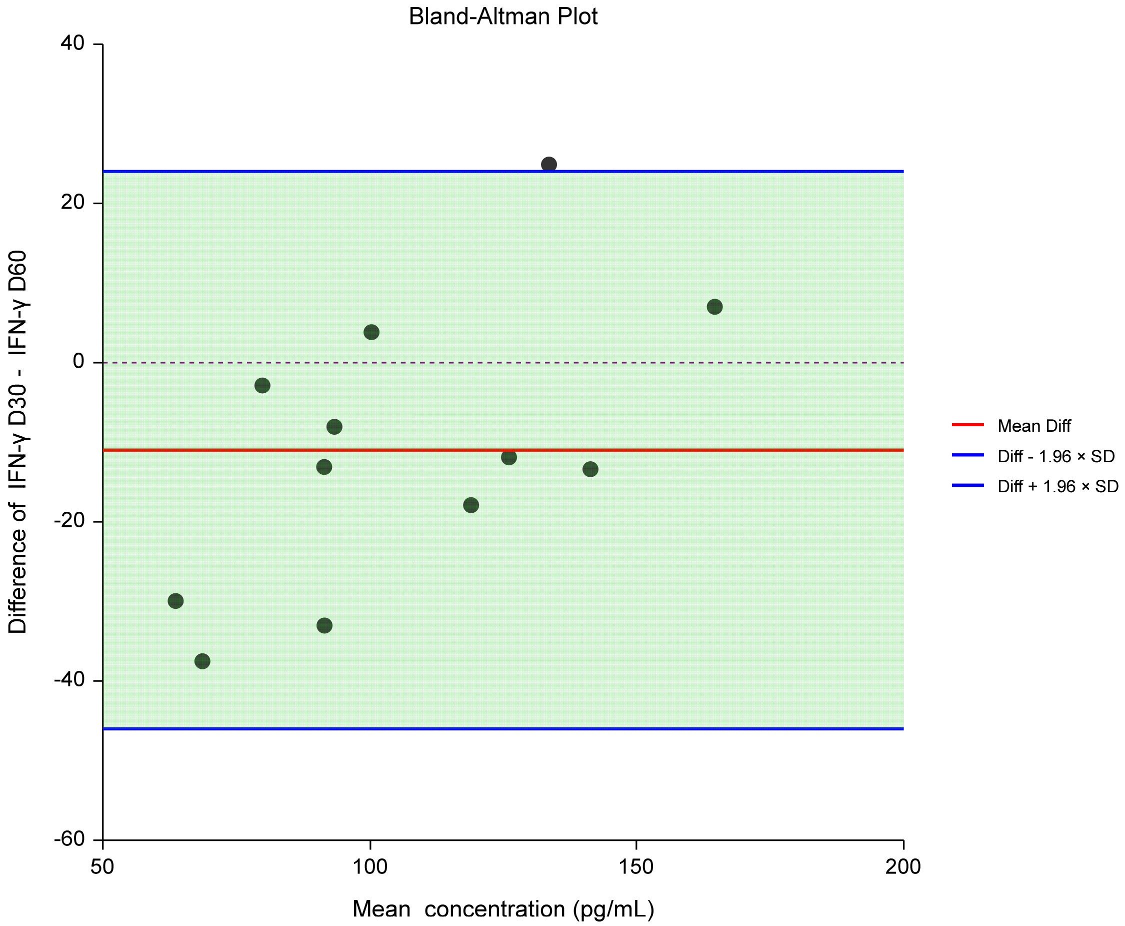

Figure S4. Bland-Altman plot of Concentration of IFN-γ for Day 30 and Day 60 in test group.

Table S4. Bias and Limits of Agreement of Concentration of IFN-γ for Day 30 and Day 60 in test group.

Parameter	Count	Value	Standard Deviation	95.0% LCL of Value	95.0% UCL of Value
Bias (Difference)	12	−11.01	17.87	−22.36	0.35
Lower Limit of Agreement	12	−46.03	9.08	−66.00	−26.05
Upper Limit of Agreement	12	24.02	9.08	4.04	43.99

Limits of Agreement = Difference ± 1.96 × (Standard Deviation of Difference).

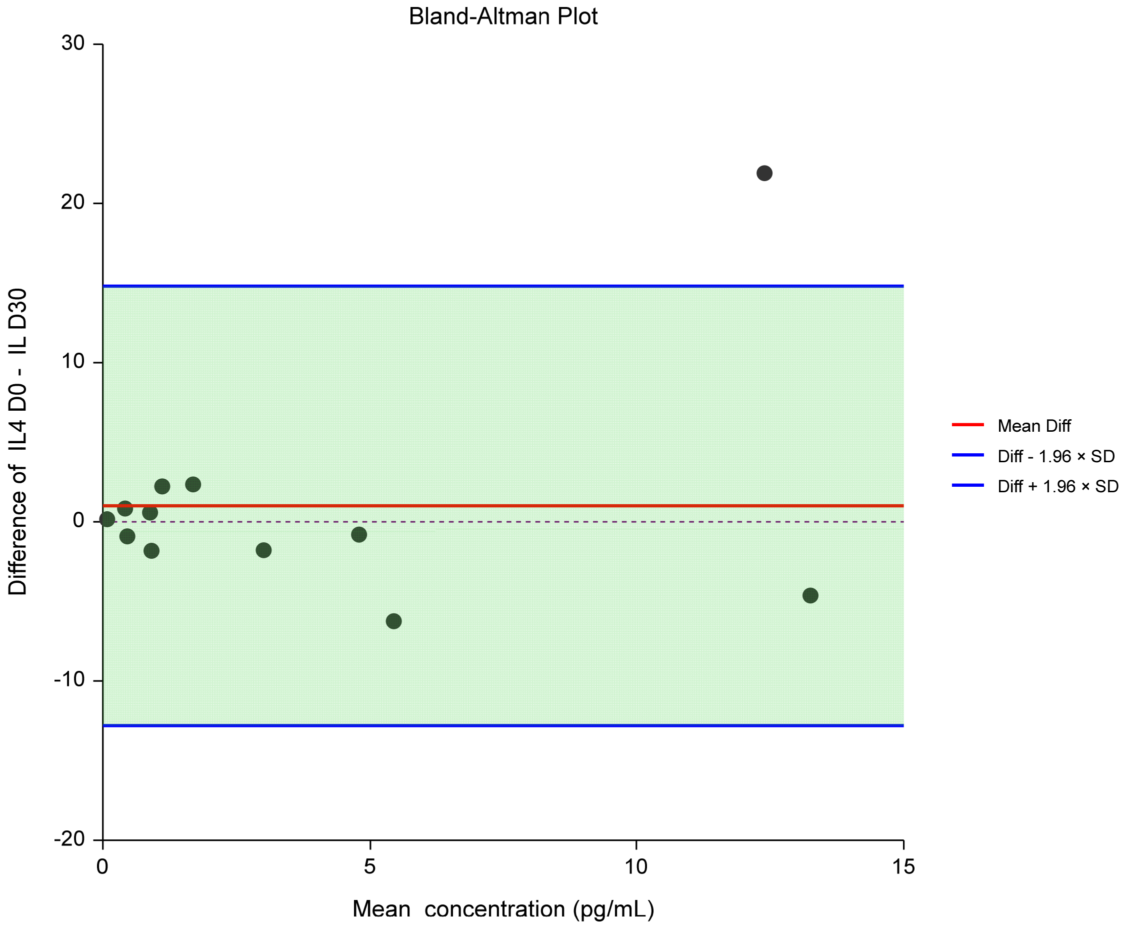

Figure S5. Bland-Altman plot of Concentration of IL-4 for Day 0 and Day 30 in placebo group.

Table S5. Bias and Limits of Agreement of Concentration of IL-4 for Day 0 and Day 30 in placebo group.

Parameter	Count	Value	Standard Deviation	95.0% LCL of Value	95.0% UCL of Value
Bias (Difference)	12	0.99	7.06	−3.50	5.47
Lower Limit of Agreement	12	−12.84	3.58	−20.73	−4.96
Upper Limit of Agreement	12	14.82	3.58	6.93	22.71

Limits of Agreement = Difference ± 1.96 × (Standard Deviation of Difference).

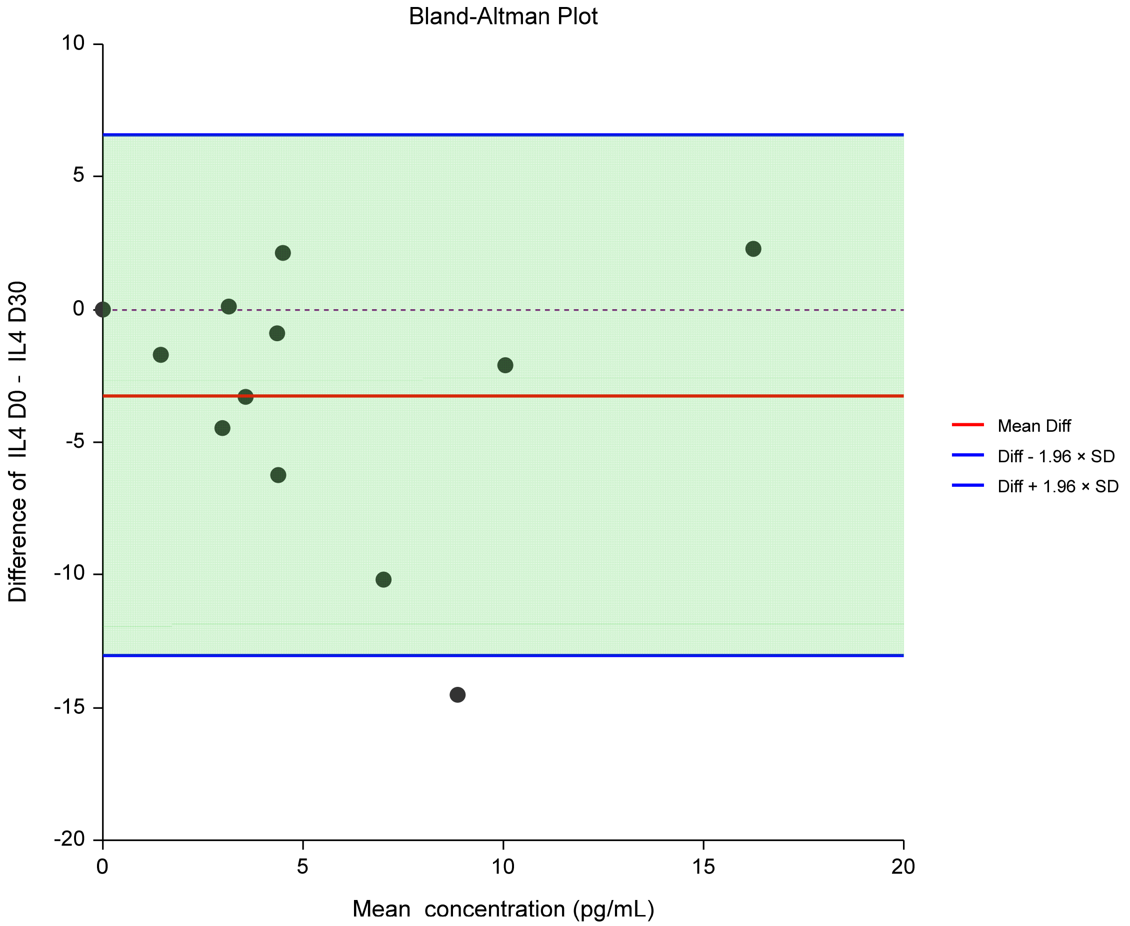

Figure S6. Bland-Altman plot of Concentration of IL-4 for Day 0 and Day 30 in test group.

Table S6. Bias and Limits of Agreement of Concentration of IL-4 for Day 0 and Day 30 in test group.

Parameter	Count	Value	Standard Deviation	95.0% LCL of Value	95.0% UCL of Value
Bias (Difference)	12	−3.24	5.01	−6.42	−0.06
Lower Limit of Agreement	12	−13.06	2.55	−18.66	−7.46
Upper Limit of Agreement	12	6.58	2.55	0.98	12.18

Limits of Agreement = Difference ± 1.96 × (Standard Deviation of Difference).

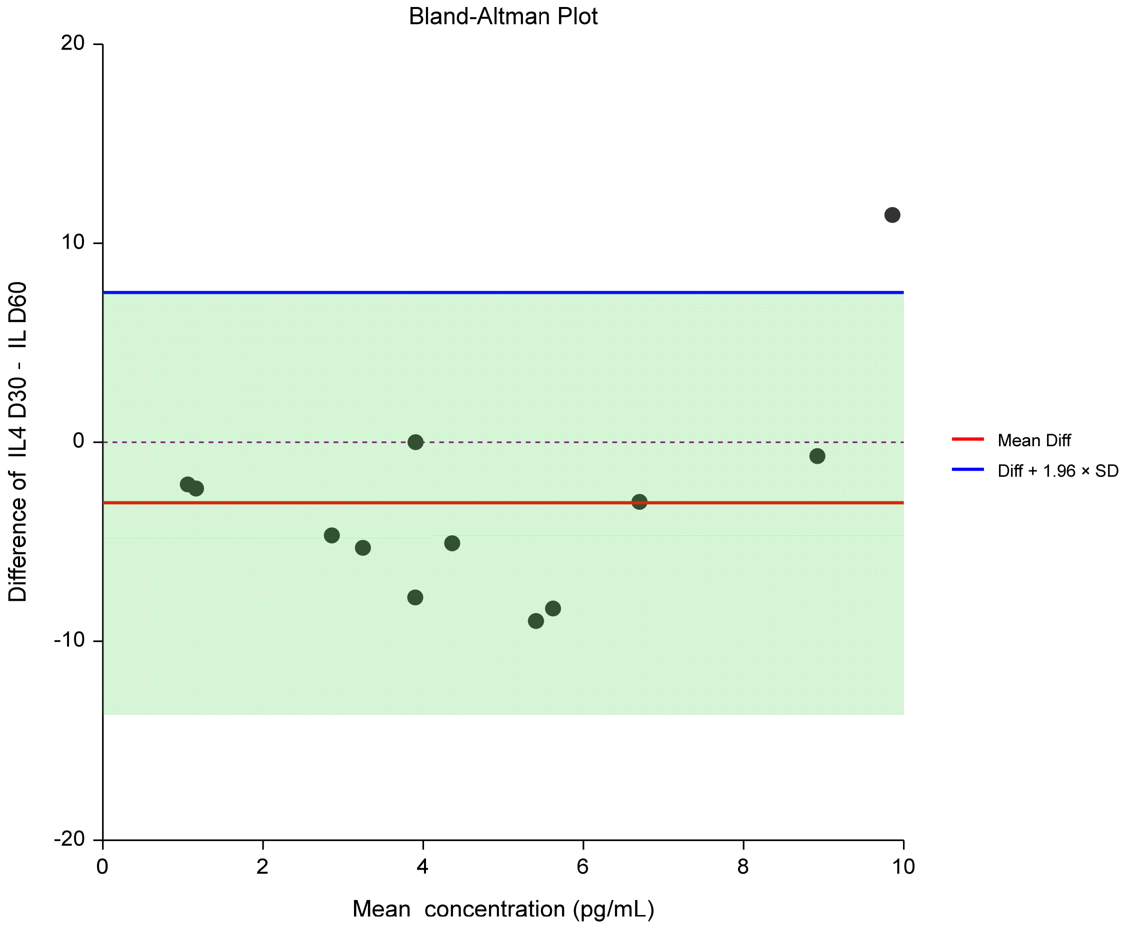

Figure S7. Bland-Altman plot of Concentration of IL-4 for Day 30 and Day 60 in placebo crossed over to test group.

Table S7. Bias and Limits of Agreement of Concentration of IL-4 for Day 30 and Day 60 in placebo crossed over to test group.

Parameter	Count	Value	Standard Deviation	95.0% LCL of Value	95.0% UCL of Value
Bias (Difference)	12	−3.08	5.42	−6.52	0.37
Lower Limit of Agreement	12	−13.71	2.75	−19.77	−7.64
Upper Limit of Agreement	12	7.55	2.75	1.49	13.61

Limits of Agreement = Difference ± 1.96 × (Standard Deviation of Difference).

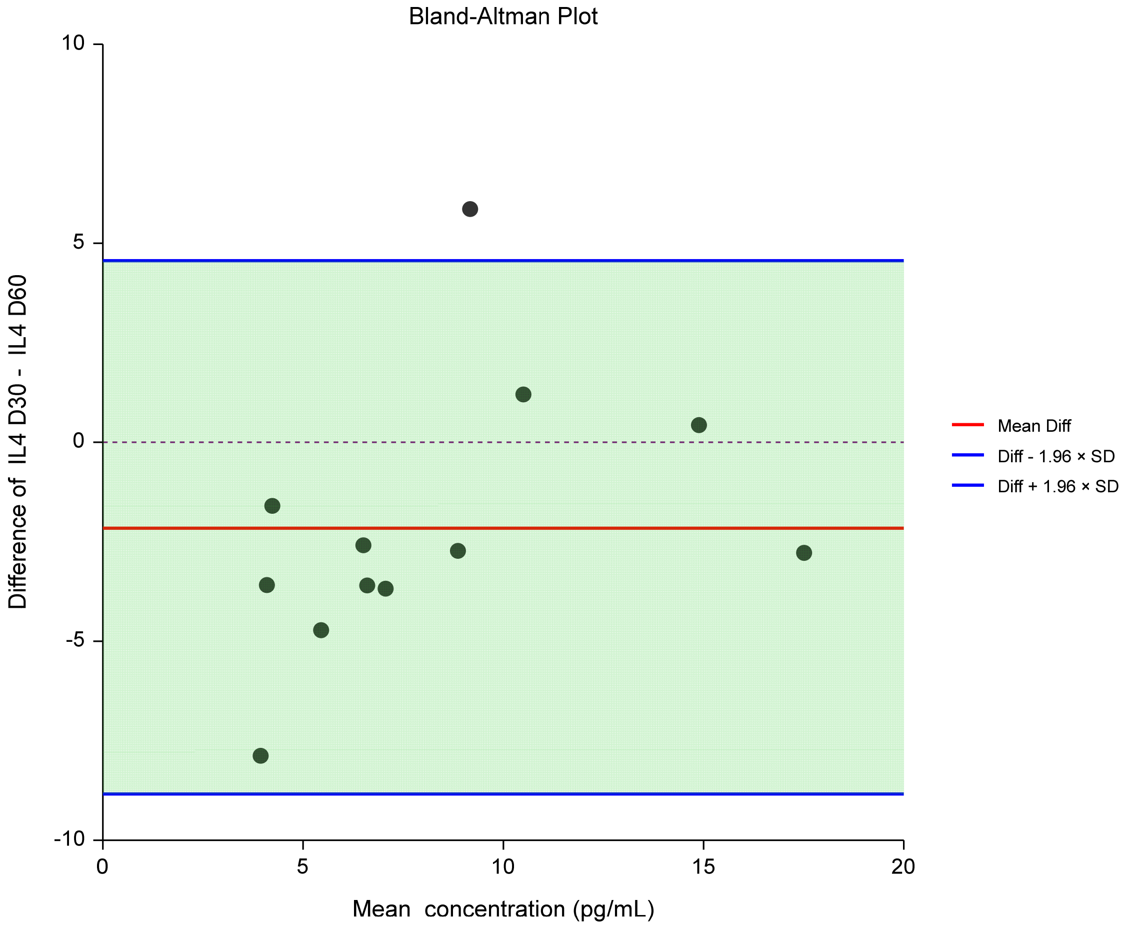

Figure S8. Bland-Altman plot of Concentration of IL-4 for Day 30 and Day 60 in test group.

Table S8. Bias and Limits of Agreement of Concentration of IL-4 for Day 30 and Day 60 in test group.

Parameter	Count	Value	Standard Deviation	95.0% LCL of Value	95.0% UCL of Value
Bias (Difference)	12	−2.14	3.43	−4.32	0.04
Lower Limit of Agreement	12	−8.86	1.74	−12.69	−5.03
Upper Limit of Agreement	12	4.58	1.74	0.75	8.41

Limits of Agreement = Difference ± 1.96 × (Standard Deviation of Difference).
