# Supplementary material for: Immunomodulatory Effect of Withania somnifera (Ashwagandha) Extract—A Randomized, Double-Blind, Placebo Controlled Trial with an Open Label Extension on Healthy Participants
Source: J Clin Med. 2021 Aug 18;10(16):3644. doi: 10.3390/jcm10163644 (PMC8397213; doi:10.3390/jcm10163644)
Supplement: Supplementary file 1 [file jcm-10-03644-s001.zip › supplementary file 4 - final.pdf]

Supplementary file 4. Bland-Altman Analysis of T cells, B cells and NK cells

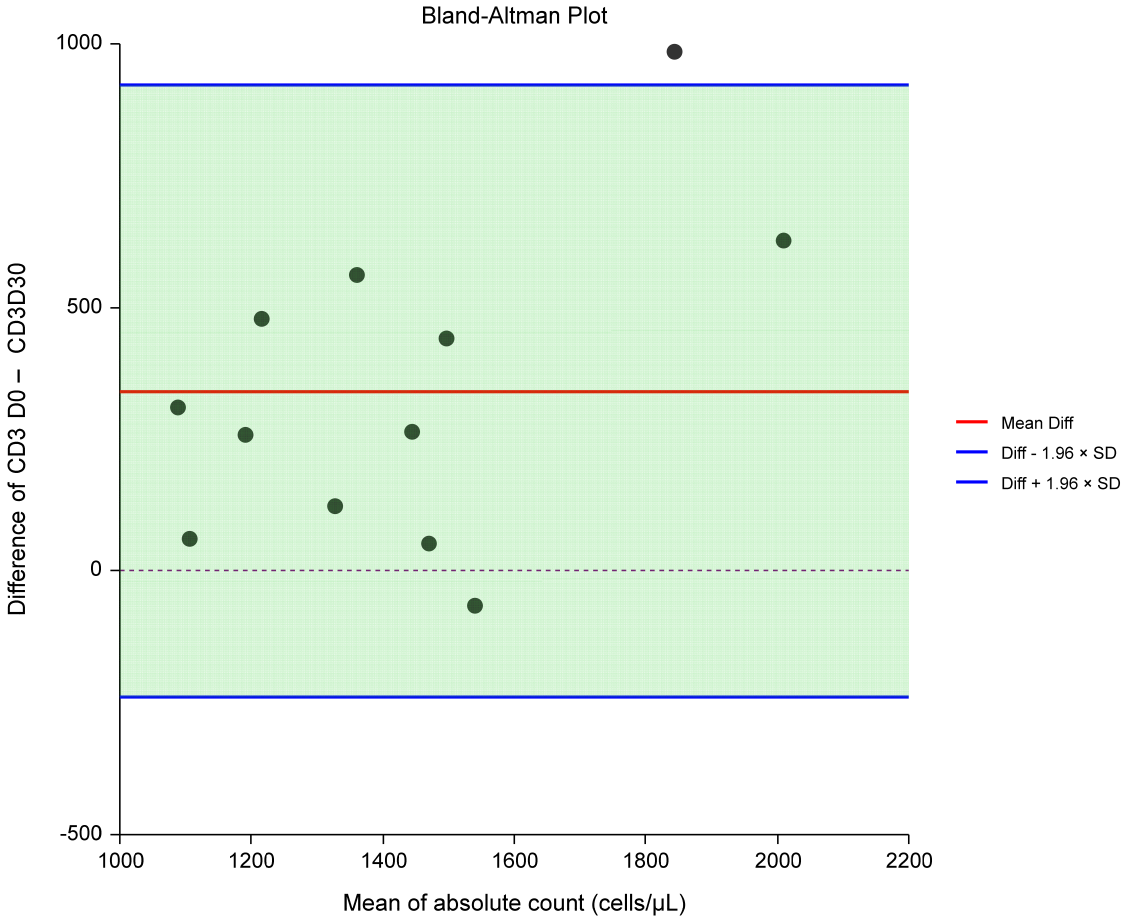

Figure S1. Bland-Altman plot of Absolute count of CD3 for Day 0 and Day 30 in placebo group.

Table S1. Bias and Limits of Agreement of Absolute count of CD3 for Day 0 and Day 30 in placebo group.

Parameter	Count	Value	Standard Deviation	95.0% LCL of Value	95.0% UCL of Value
Bias (Difference)	12	341.22	295.78	153.29	529.15
Lower Limit of Agreement	12	−238.50	150.22	−569.14	92.13
Upper Limit of Agreement	12	920.95	150.22	590.31	1251.59

Limits of Agreement = Difference ± 1.96 × (Standard Deviation of Difference)

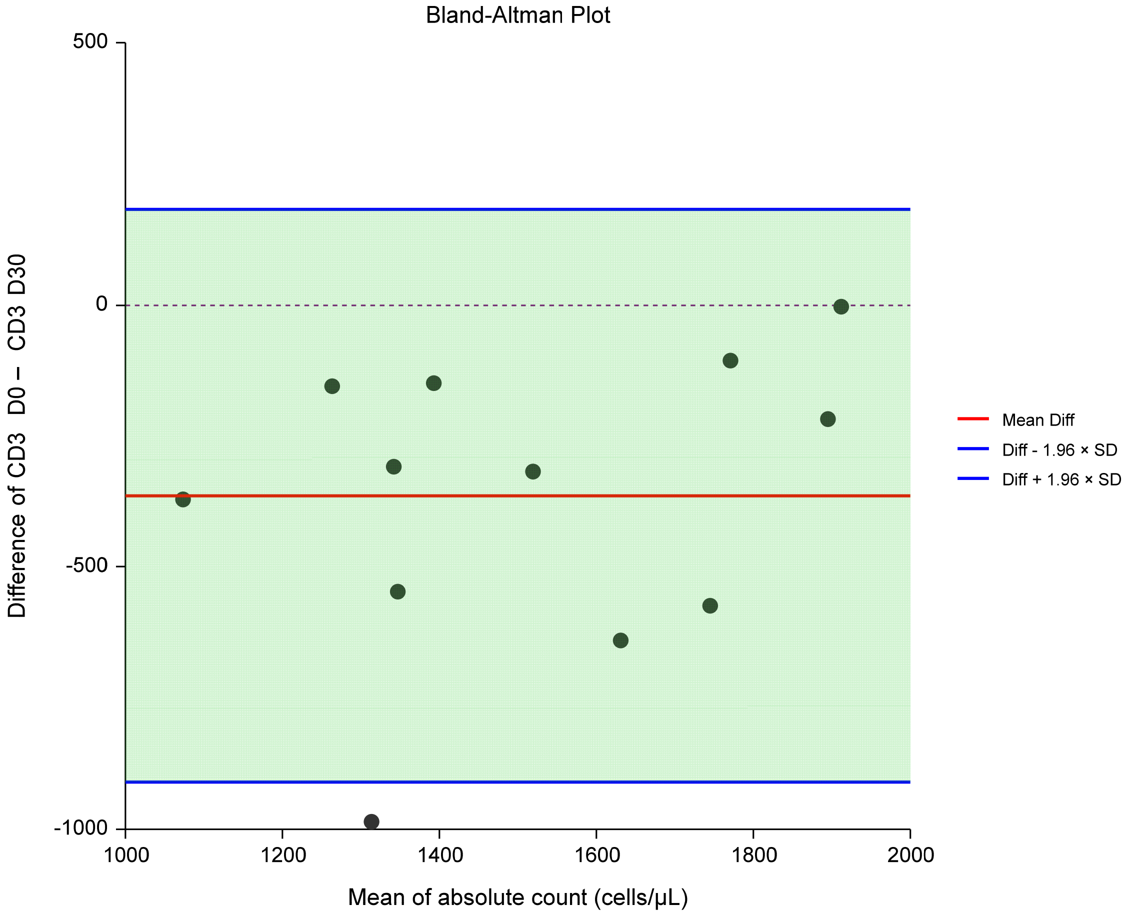

Figure S2. Bland-Altman plot of Absolute count of CD3 for Day 0 and Day 30 in test group.

Table S2. Bias and Limits of Agreement of Absolute count of CD3 for Day 0 and Day 30 in test group.

Parameter	Count	Value	Standard Deviation	95.0% LCL of Value	95.0% UCL of Value
Bias (Difference)	12	−364.69	278.50	−541.64	−187.74
Lower Limit of Agreement	12	−910.54	141.45	−1221.86	−599.23
Upper Limit of Agreement	12	181.16	141.45	−130.16	492.48

Limits of Agreement = Difference ± 1.96 × (Standard Deviation of Difference)

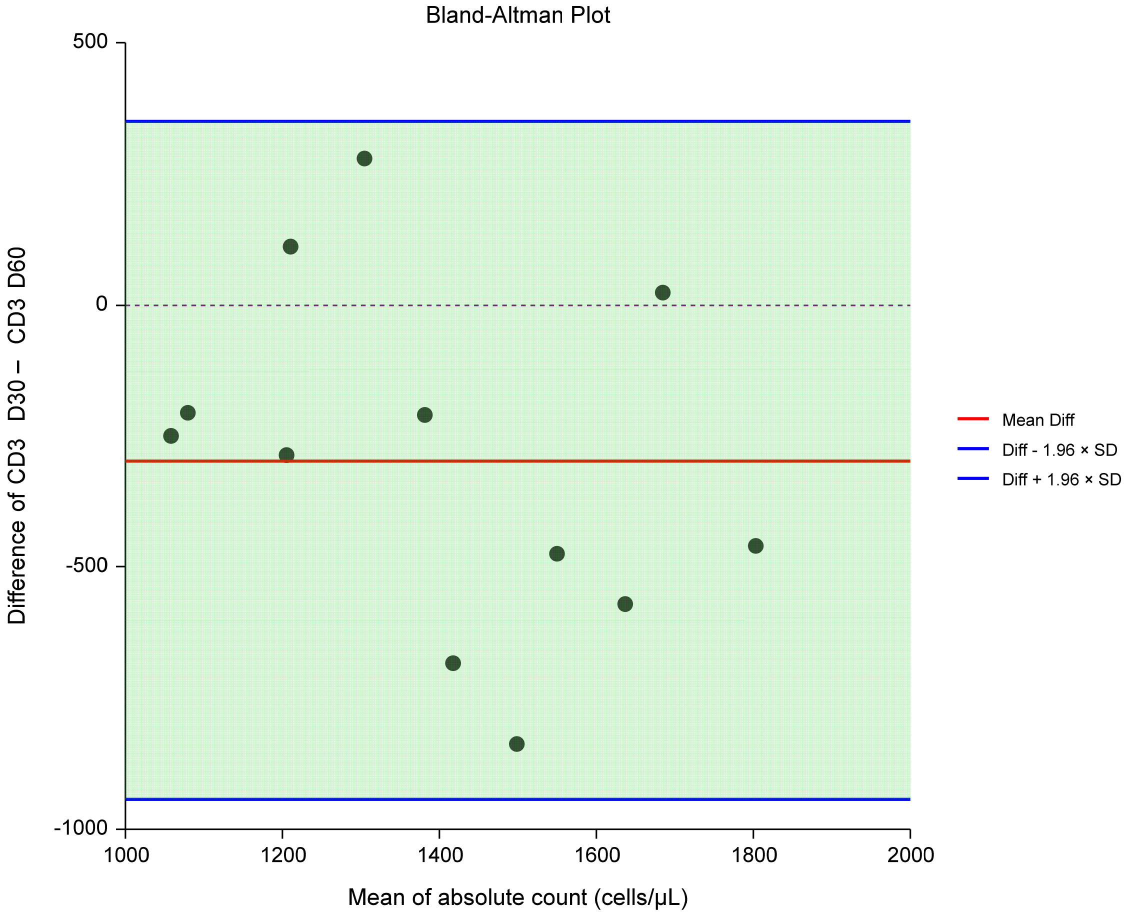

Figure S3. Bland-Altman plot of Absolute count of CD3 for Day 30 and Day 60 in placebo crossed over to test group.

Table S3. Bias and Limits of Agreement of Absolute count of CD3 for Day 30 and Day 60 in placebo crossed over to test group.

Parameter	Count	Value	Standard Deviation	95.0% LCL of Value	95.0% UCL of Value
Bias (Difference)	12	−296.99	329.43	−506.29	−87.68
Lower Limit of Agreement	12	−942.66	167.31	−1310.92	−574.41
Upper Limit of Agreement	12	348.69	167.31	−19.56	716.95

Limits of Agreement = Difference ± 1.96 × (Standard Deviation of Difference)

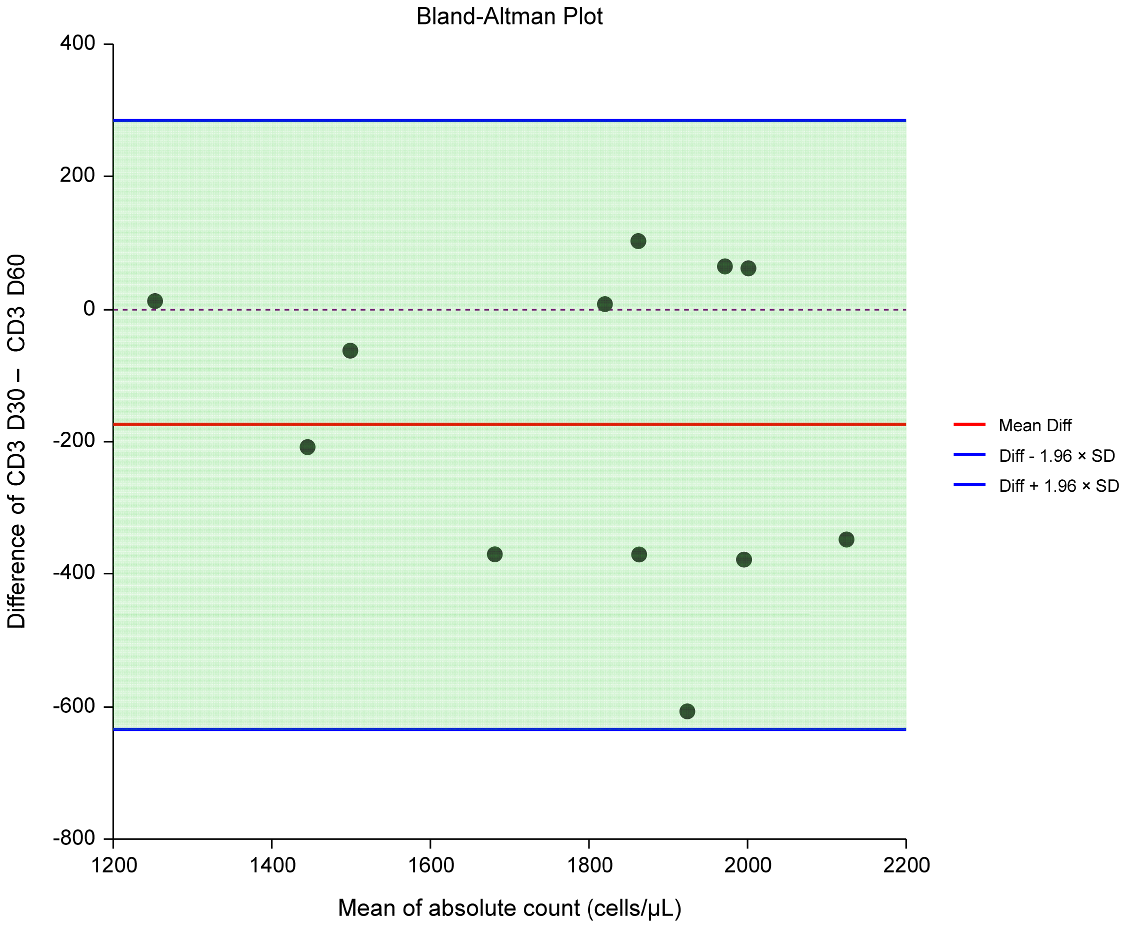

Figure S4. Bland-Altman plot of Absolute count of CD3 for Day 30 and Day 60 in test group.

Table S4. Bias and Limits of Agreement of Absolute count of CD3 for Day 30 and Day 60 in test group.

Parameter	Count	Value	Standard Deviation	95.0% LCL of Value	95.0% UCL of Value
Bias (Difference)	12	−174.61	234.89	−323.86	−25.37
Lower Limit of Agreement	12	−635.01	119.30	−897.58	−372.43
Upper Limit of Agreement	12	285.78	119.30	23.20	548.35

Limits of Agreement = Difference ± 1.96 × (Standard Deviation of Difference)

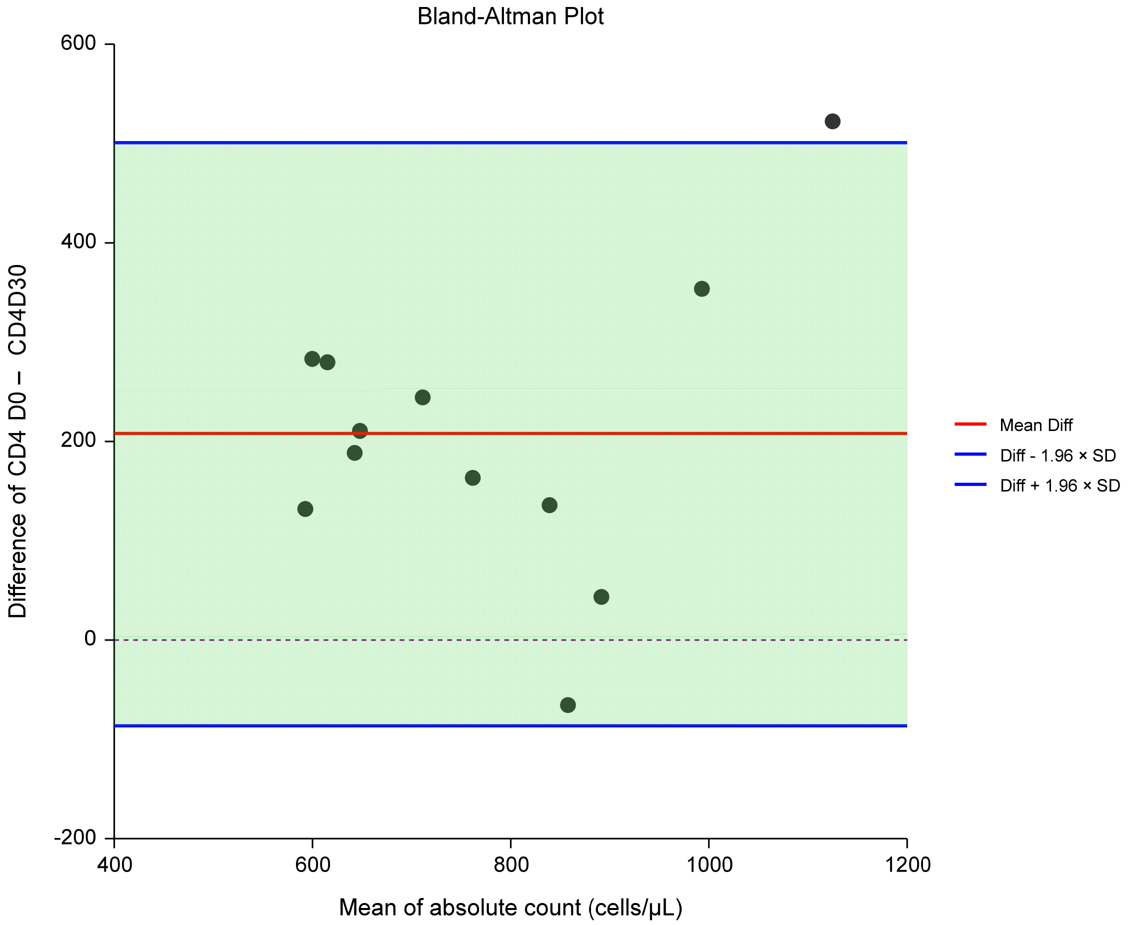

Figure S5. Bland-Altman plot of Absolute count of CD4 for Day 0 and Day 30 in placebo group.

Table S5. Bias and Limits of Agreement of Absolute count of CD4 for Day 0 and Day 30 in placebo group.

Parameter	Count	Value	Standard Deviation	95.0% LCL of Value	95.0% UCL of Value
Bias (Difference)	12	207.58	149.96	112.30	302.86
Lower Limit of Agreement	12	−86.35	76.16	−253.99	81.29
Upper Limit of Agreement	12	501.51	76.16	333.87	669.15

Limits of Agreement = Difference ± 1.96 × (Standard Deviation of Difference)

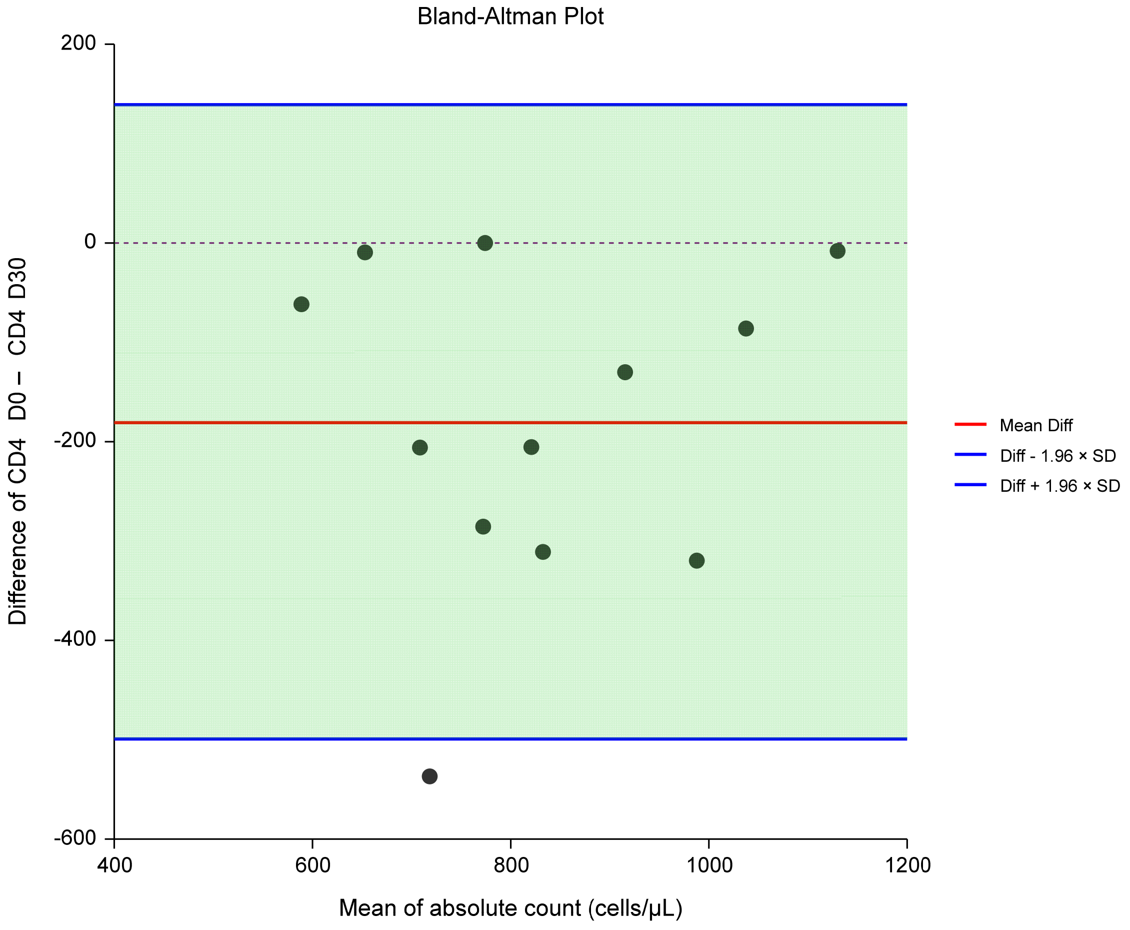

Figure S6. Bland-Altman plot of Absolute count of CD4 for Day 0 and Day 30 in test group.

Table S6. Bias and Limits of Agreement of Absolute count of CD4 for Day 0 and Day 30 in test group.

Parameter	Count	Value	Standard Deviation	95.0% LCL of Value	95.0% UCL of Value
Bias (Difference)	12	−180.02	163.15	−283.68	−76.36
Lower Limit of Agreement	12	−499.80	82.86	−682.19	−317.42
Upper Limit of Agreement	12	139.76	82.86	−42.62	322.15

Limits of Agreement = Difference ± 1.96 × (Standard Deviation of Difference)

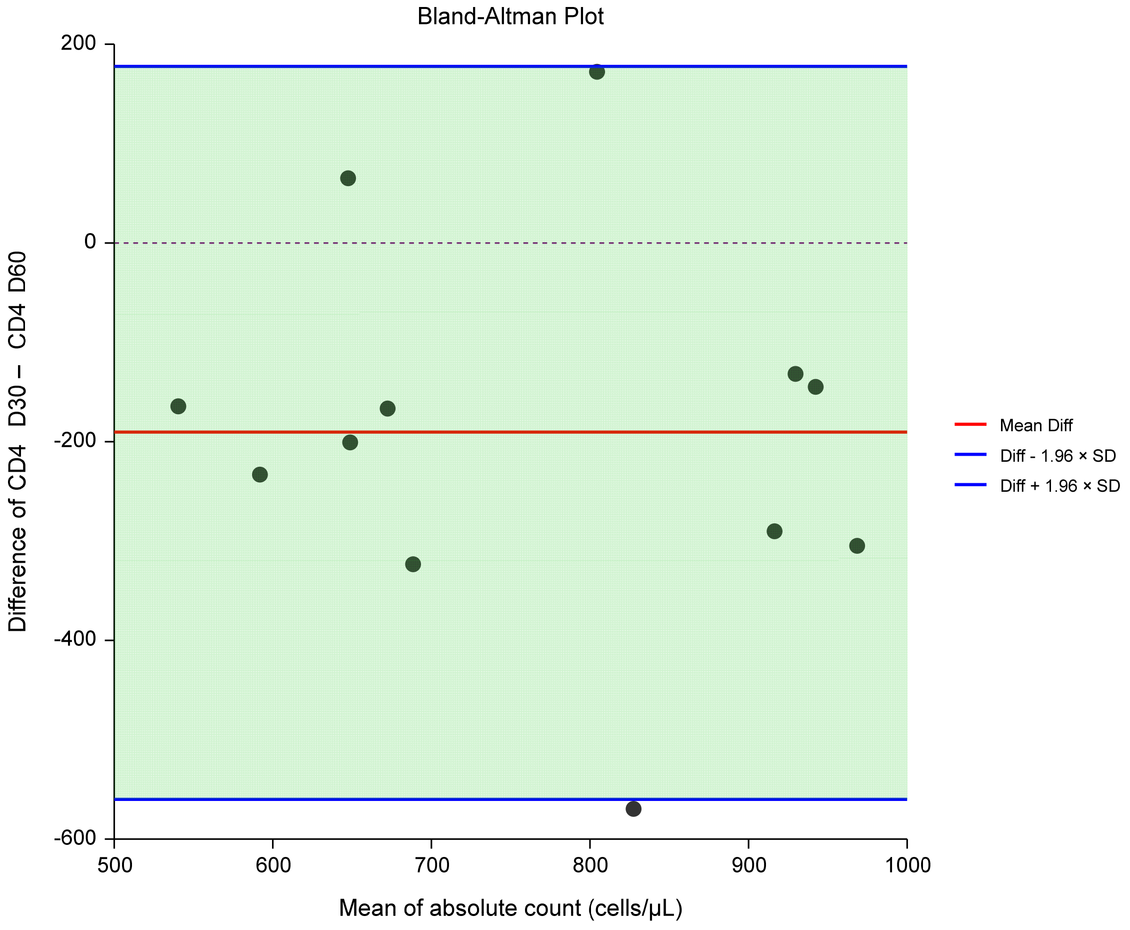

Figure S7. Bland-Altman plot of Absolute count of CD4 for Day 30 and Day 60 in placebo crossed over to test group.

Table S7. Bias and Limits of Agreement of Absolute count of CD4 for Day 30 and Day 60 in placebo crossed over to test group.

Parameter	Count	Value	Standard Deviation	95.0% LCL of Value	95.0% UCL of Value
Bias (Difference)	12	−191.04	188.31	−310.68	−71.40
Lower Limit of Agreement	12	−560.12	95.64	−770.62	−349.62
Upper Limit of Agreement	12	178.04	95.64	−32.46	388.54

Limits of Agreement = Difference ± 1.96 × (Standard Deviation of Difference)

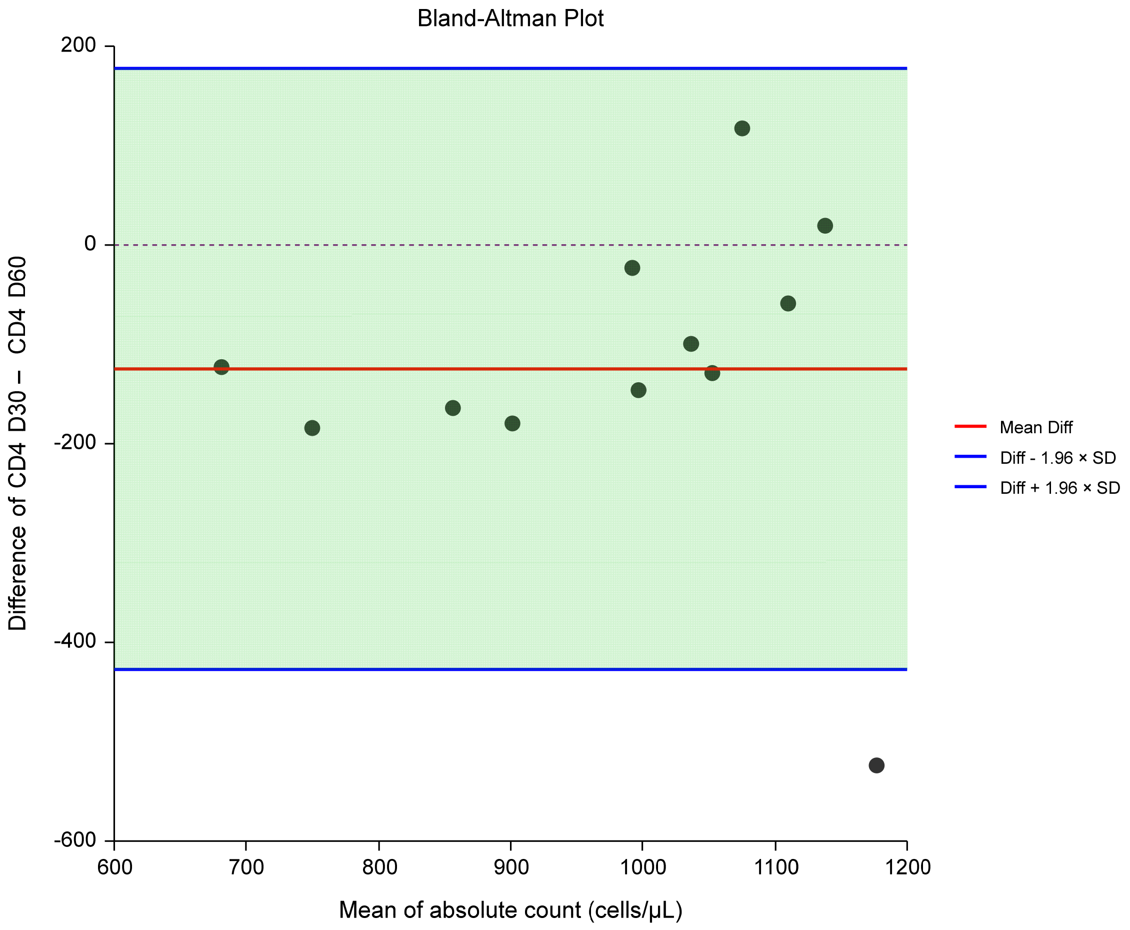

Figure S8. Bland-Altman plot of Absolute count of CD4 for Day 30 and Day 60 in test group.

Table S8. Bias and Limits of Agreement of Absolute count of CD4 for Day 30 and Day 60 in test group.

Parameter	Count	Value	Standard Deviation	95.0% LCL of Value	95.0% UCL of Value
Bias (Difference)	12	−124.57	154.50	−222.73	−26.40
Lower Limit of Agreement	12	−427.38	78.47	−600.09	−254.68
Upper Limit of Agreement	12	178.25	78.47	5.54	350.95

Limits of Agreement = Difference ± 1.96 × (Standard Deviation of Difference)

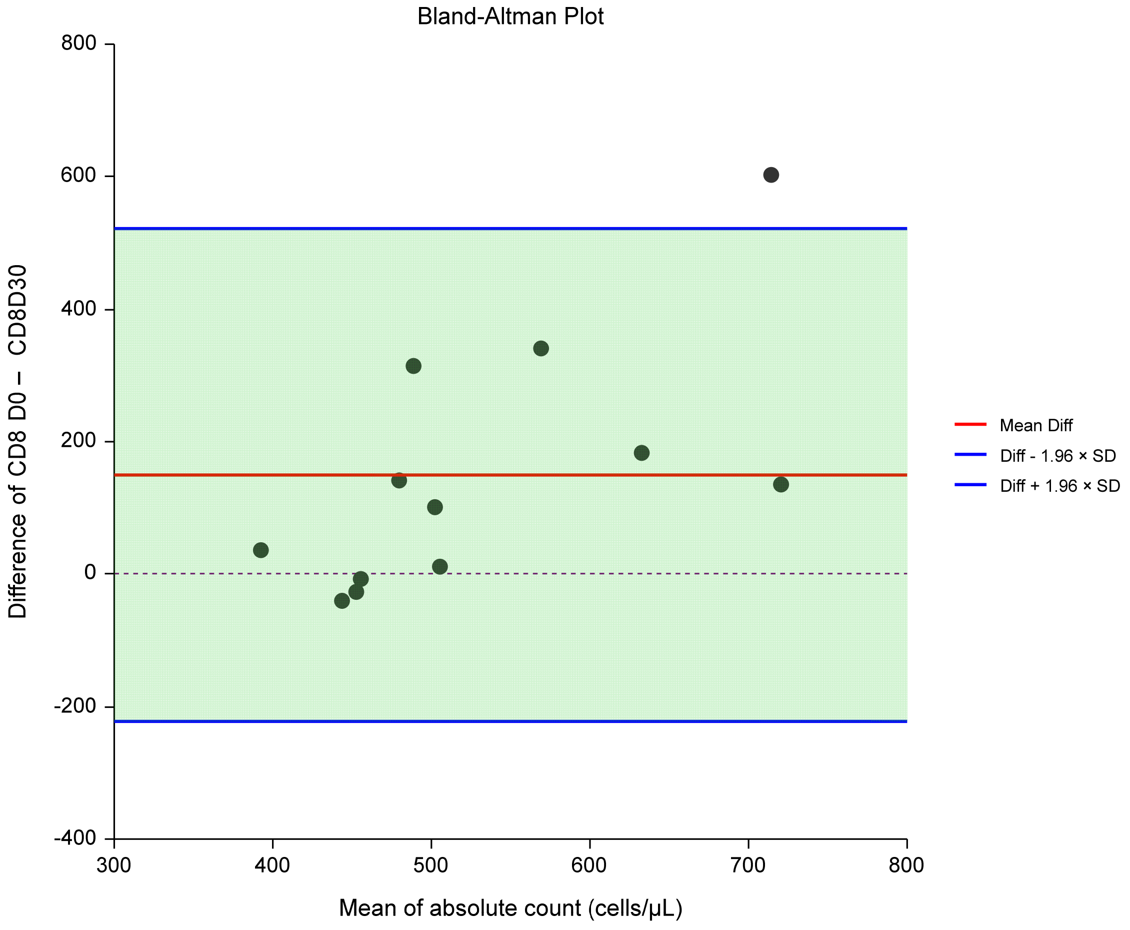

Figure S9. Bland-Altman plot of Absolute count of CD8 for Day 0 and Day 30 in placebo group.

Table S9. Bias and Limits of Agreement of Absolute count of CD8 for Day 0 and Day 30 in placebo group.

Parameter	Count	Value	Standard Deviation	95.0% LCL of Value	95.0% UCL of Value
Bias (Difference)	12	149.32	189.88	28.67	269.96
Lower Limit of Agreement	12	−222.86	96.44	−435.12	−10.59
Upper Limit of Agreement	12	521.49	96.44	309.22	733.75

Limits of Agreement = Difference ± 1.96 × (Standard Deviation of Difference)

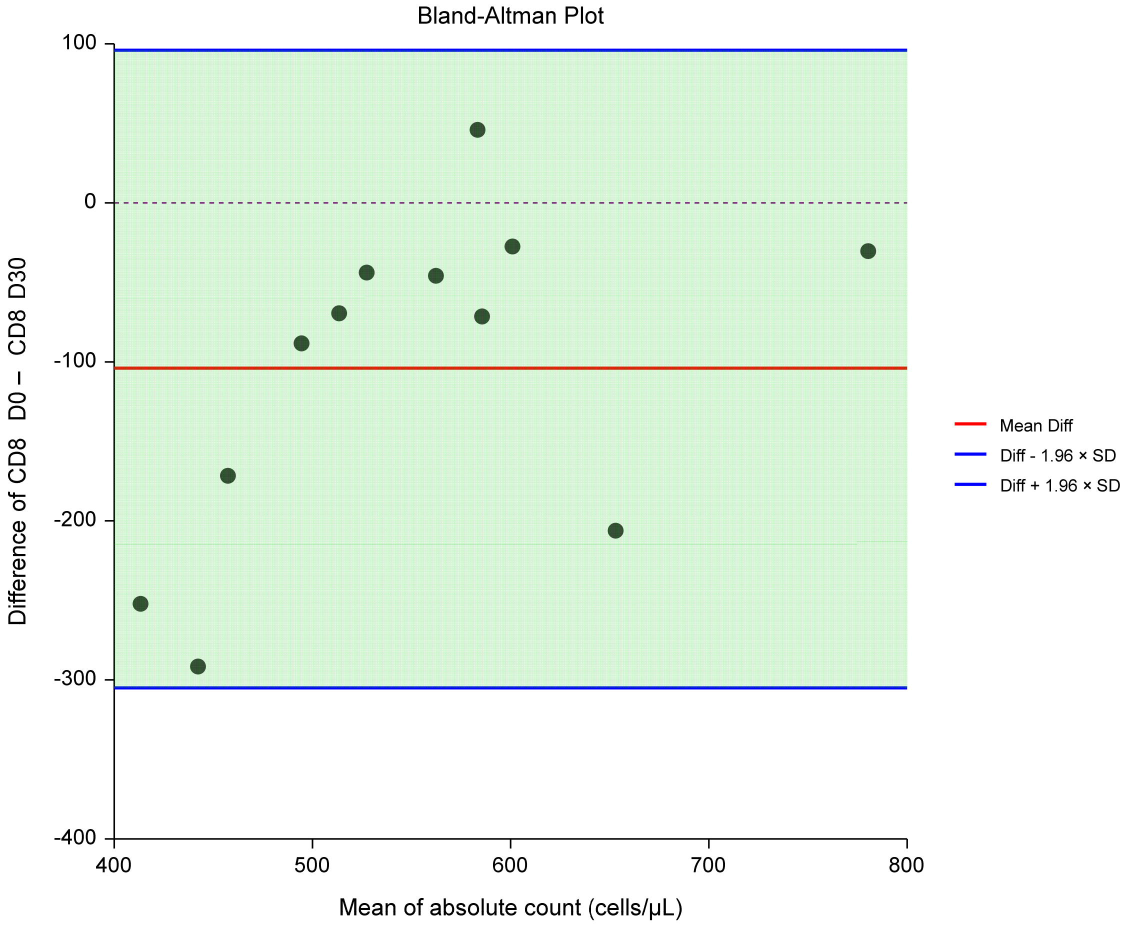

Figure S10. Bland-Altman plot of Absolute count of CD8 for Day 0 and Day 30 in test group.

Table S10. Bias and Limits of Agreement of Absolute count of CD8 for Day 0 and Day 30 in test group.

Parameter	Count	Value	Standard Deviation	95.0% LCL of Value	95.0% UCL of Value
Bias (Difference)	12	−104.36	102.43	−169.44	−39.28
Lower Limit of Agreement	12	−305.13	52.03	−419.64	−190.62
Upper Limit of Agreement	12	96.41	52.03	−18.09	210.92

Limits of Agreement = Difference ± 1.96 × (Standard Deviation of Difference)

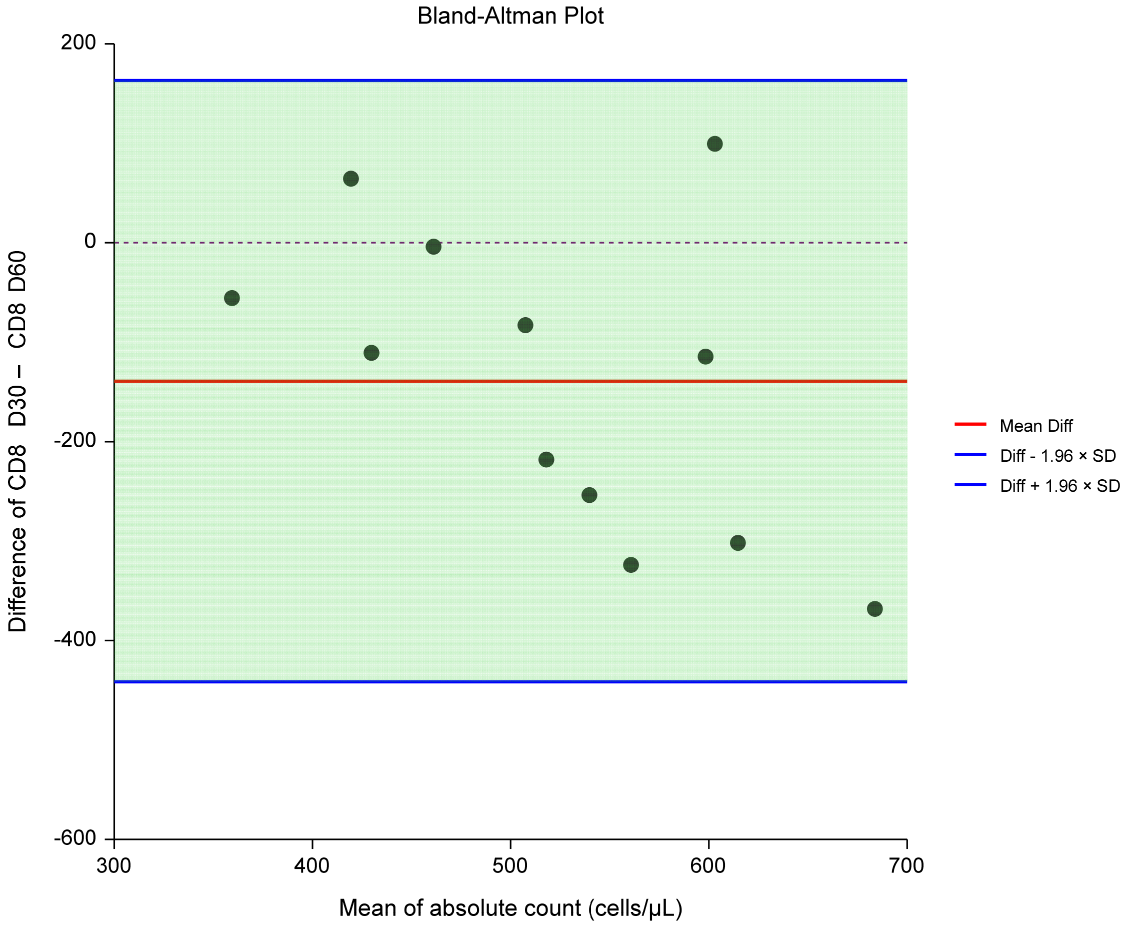

Figure S11. Bland-Altman plot of Absolute count of CD8 for Day 30 and Day 60 in placebo crossed over to test group.

Table S11. Bias and Limits of Agreement of Absolute count of CD8 for Day 30 and Day 60 in placebo crossed over to test group.

Parameter	Count	Value	Standard Deviation	95.0% LCL of Value	95.0% UCL of Value
Bias (Difference)	12	−139.12	153.95	−236.94	−41.31
Lower Limit of Agreement	12	−440.86	78.19	−612.95	−268.77
Upper Limit of Agreement	12	162.61	78.19	−9.48	334.71

Limits of Agreement = Difference ± 1.96 × (Standard Deviation of Difference)

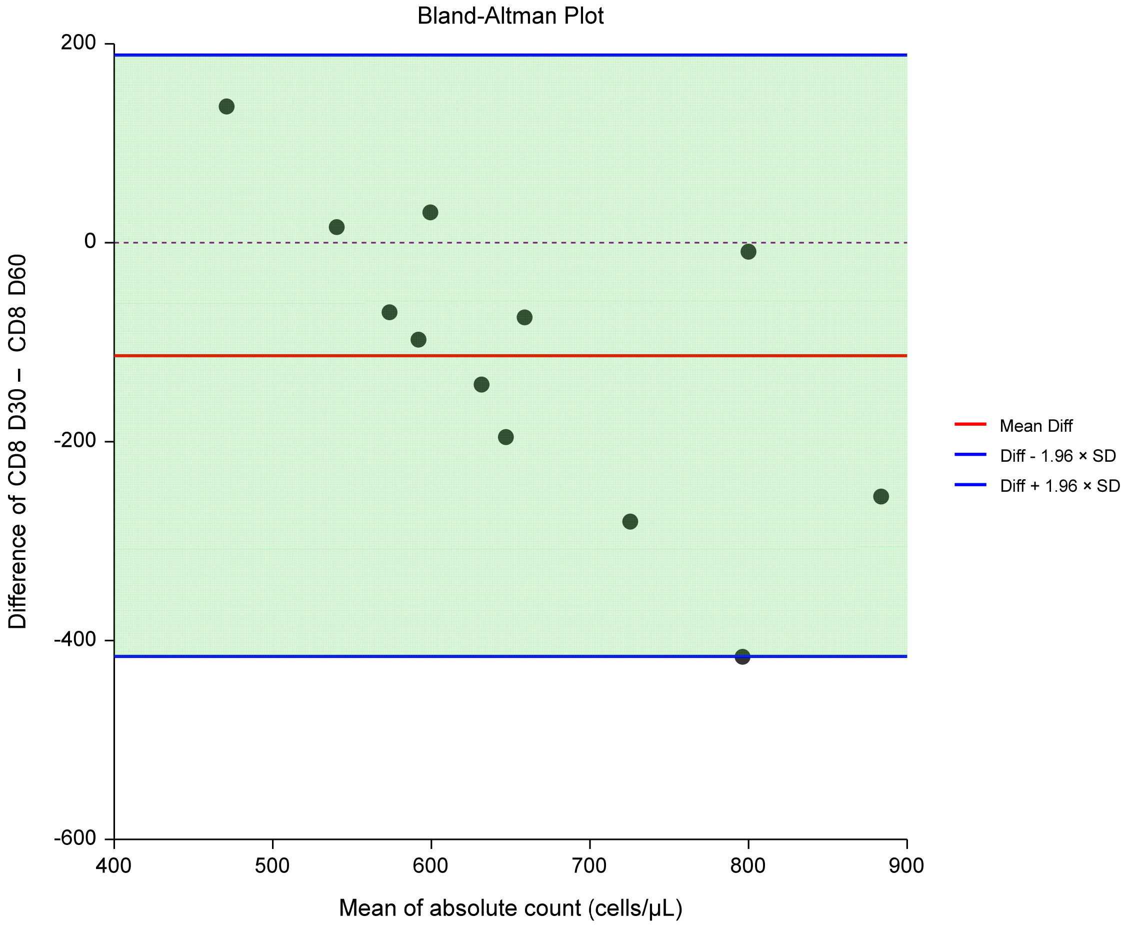

Figure S12. Bland-Altman plot of Absolute count of CD8 for Day 30 and Day 60 in test group.

Table S12. Bias and Limits of Agreement of Absolute count of CD8 for Day 30 and Day 60 in test group.

Parameter	Count	Value	Standard Deviation	95.0% LCL of Value	95.0% UCL of Value
Bias (Difference)	12	−113.16	154.35	−211.23	−15.09
Lower Limit of Agreement	12	−415.68	78.39	−588.22	−243.14
Upper Limit of Agreement	12	189.36	78.39	16.82	361.90

Limits of Agreement = Difference ± 1.96 × (Standard Deviation of Difference)

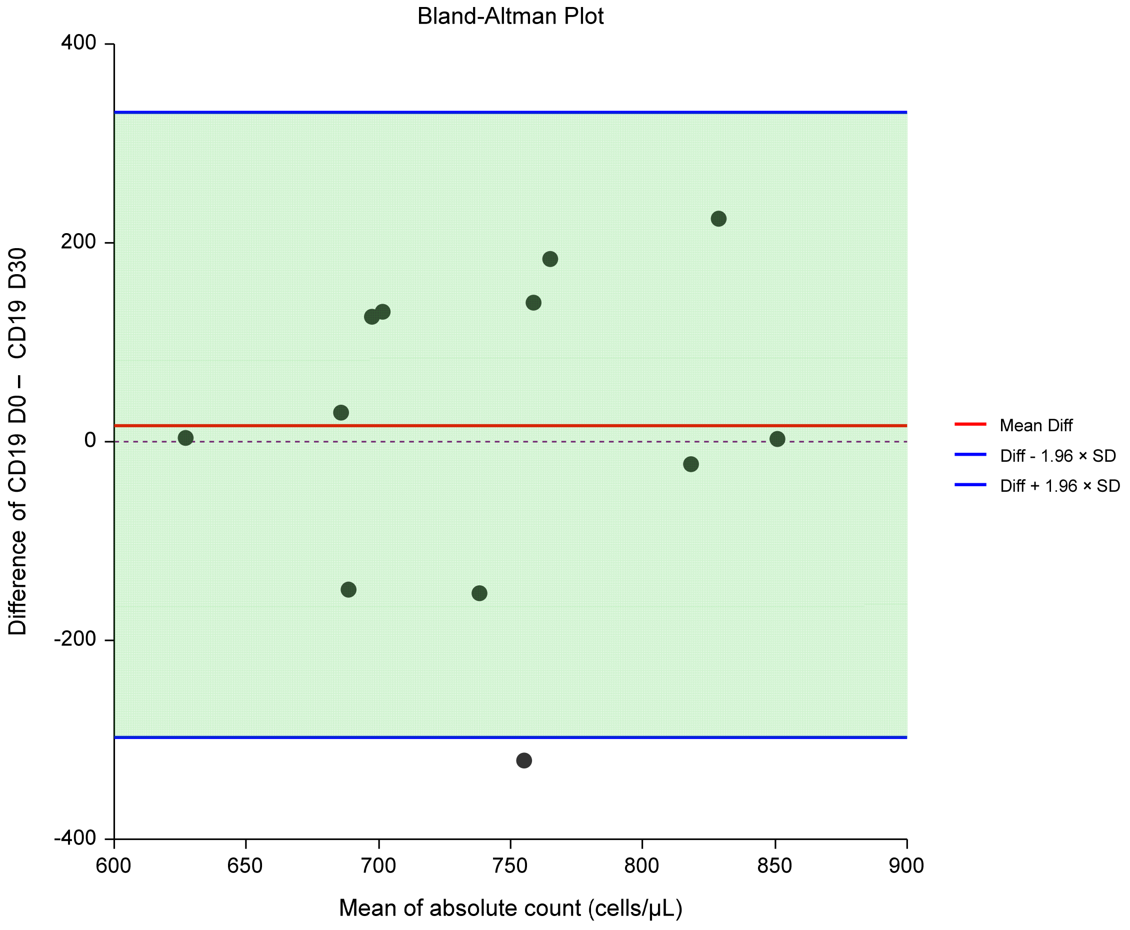

Figure S13. Bland-Altman plot of Absolute count of CD19 for Day 0 and Day 30 in placebo group.

Table S13. Bias and Limits of Agreement of Absolute count of CD19 for Day 0 and Day 30 in placebo group.

Parameter	Count	Value	Standard Deviation	95.0% LCL of Value	95.0% UCL of Value
Bias (Difference)	12	16.23	160.42	−85.70	118.16
Lower Limit of Agreement	12	−298.20	81.48	−477.53	−118.87
Upper Limit of Agreement	12	330.66	81.48	151.33	509.99

Limits of Agreement = Difference ± 1.96 × (Standard Deviation of Difference)

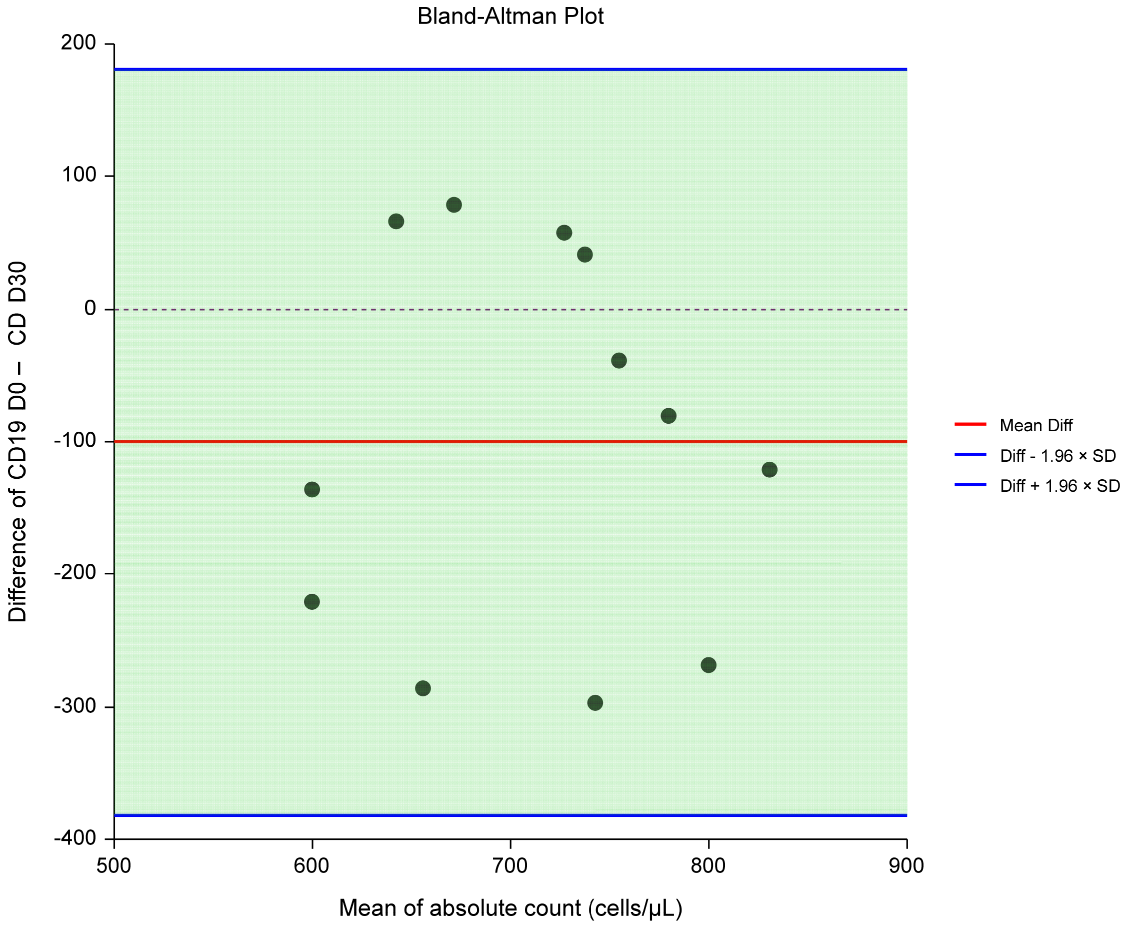

Figure S14. Bland-Altman plot of Absolute count of CD19 for Day 0 and Day 30 in test group.

Table S14. Bias and Limits of Agreement of Absolute count of CD19 for Day 0 and Day 30 in test group.

Parameter	Count	Value	Standard Deviation	95.0% LCL of Value	95.0% UCL of Value
Bias (Difference)	12	−100.45	143.57	−191.67	−9.23
Lower Limit of Agreement	12	−381.84	72.92	−542.33	−221.36
Upper Limit of Agreement	12	180.94	72.92	20.45	341.43

Limits of Agreement = Difference ± 1.96 × (Standard Deviation of Difference)

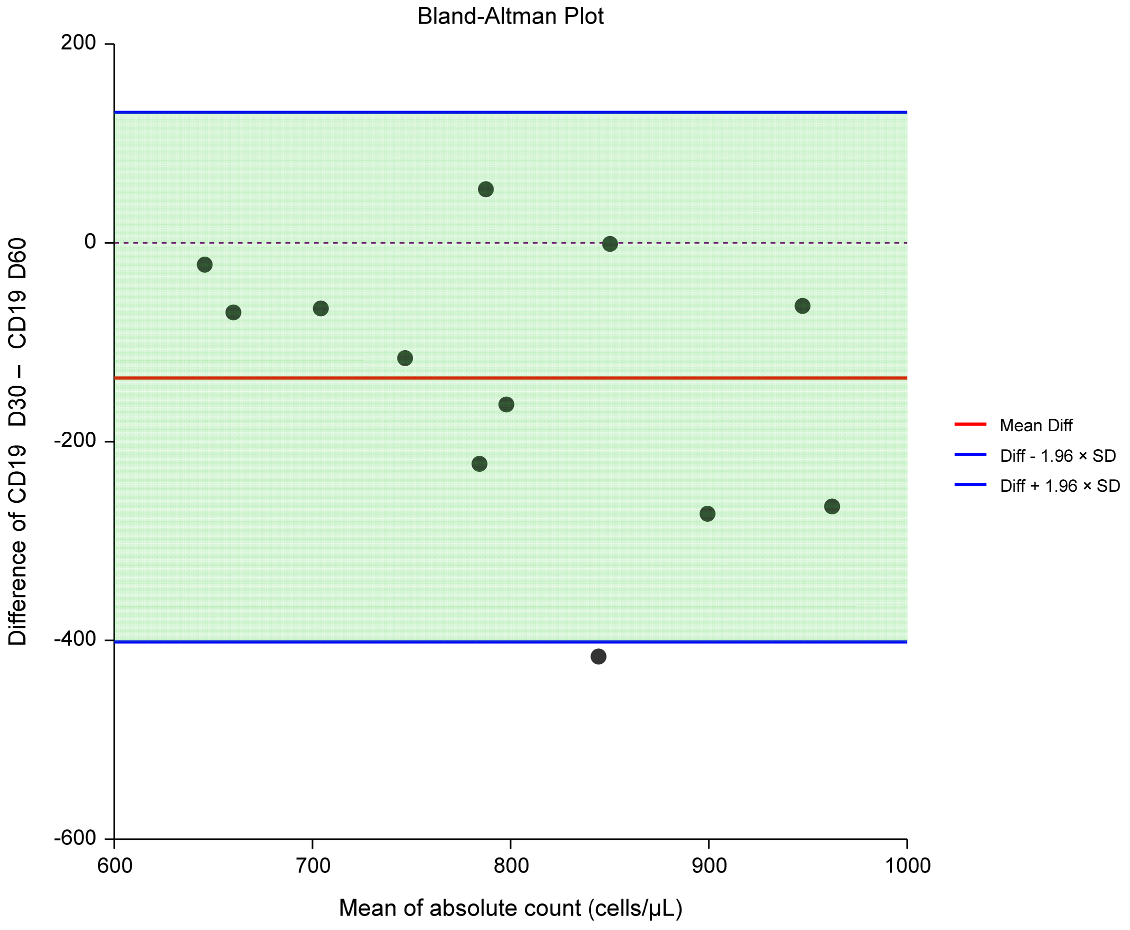

Figure S15. Bland-Altman plot of Absolute count of CD19 for Day 30 and Day 60 in placebo crossed over to test group.

Table S15. Bias and Limits of Agreement of Absolute count of CD19 for Day 30 and Day 60 in placebo crossed over to test group.

Parameter	Count	Value	Standard Deviation	95.0% LCL of Value	95.0% UCL of Value
Bias (Difference)	12	−135.27	136.26	−221.84	−48.70
Lower Limit of Agreement	12	−402.33	69.20	−554.65	−250.02
Upper Limit of Agreement	12	131.79	69.20	−20.52	284.11

Limits of Agreement = Difference ± 1.96 × (Standard Deviation of Difference)

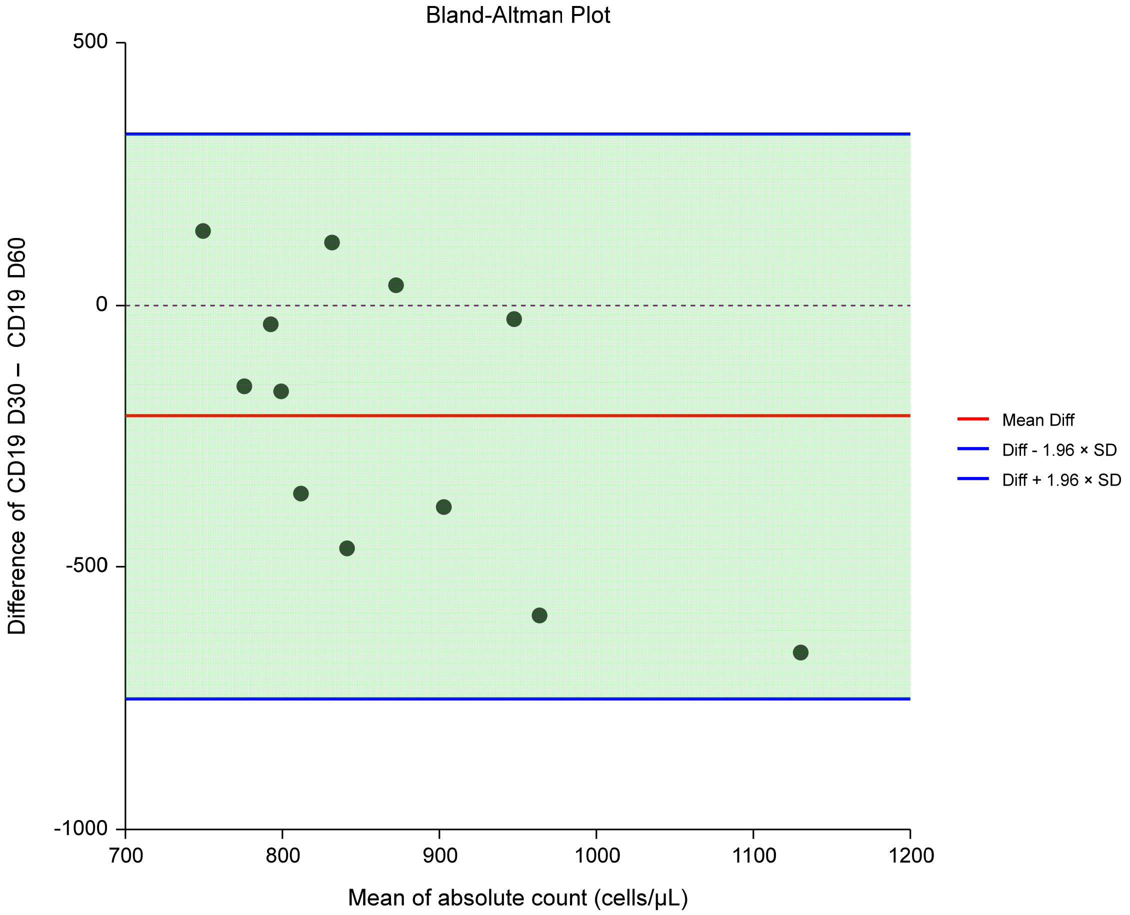

Figure S16. Bland-Altman plot of Absolute count of CD19 for Day 30 and Day 60 in test group.

Table S16. Bias and Limits of Agreement of Absolute count of CD19 for Day 30 and Day 60 in test group.

Parameter	Count	Value	Standard Deviation	95.0% LCL of Value	95.0% UCL of Value
Bias (Difference)	12	−212.41	274.66	−386.92	−37.91
Lower Limit of Agreement	12	−750.74	139.49	−1057.77	−443.71
Upper Limit of Agreement	12	325.91	139.49	18.89	632.94

Limits of Agreement = Difference ± 1.96 × (Standard Deviation of Difference)

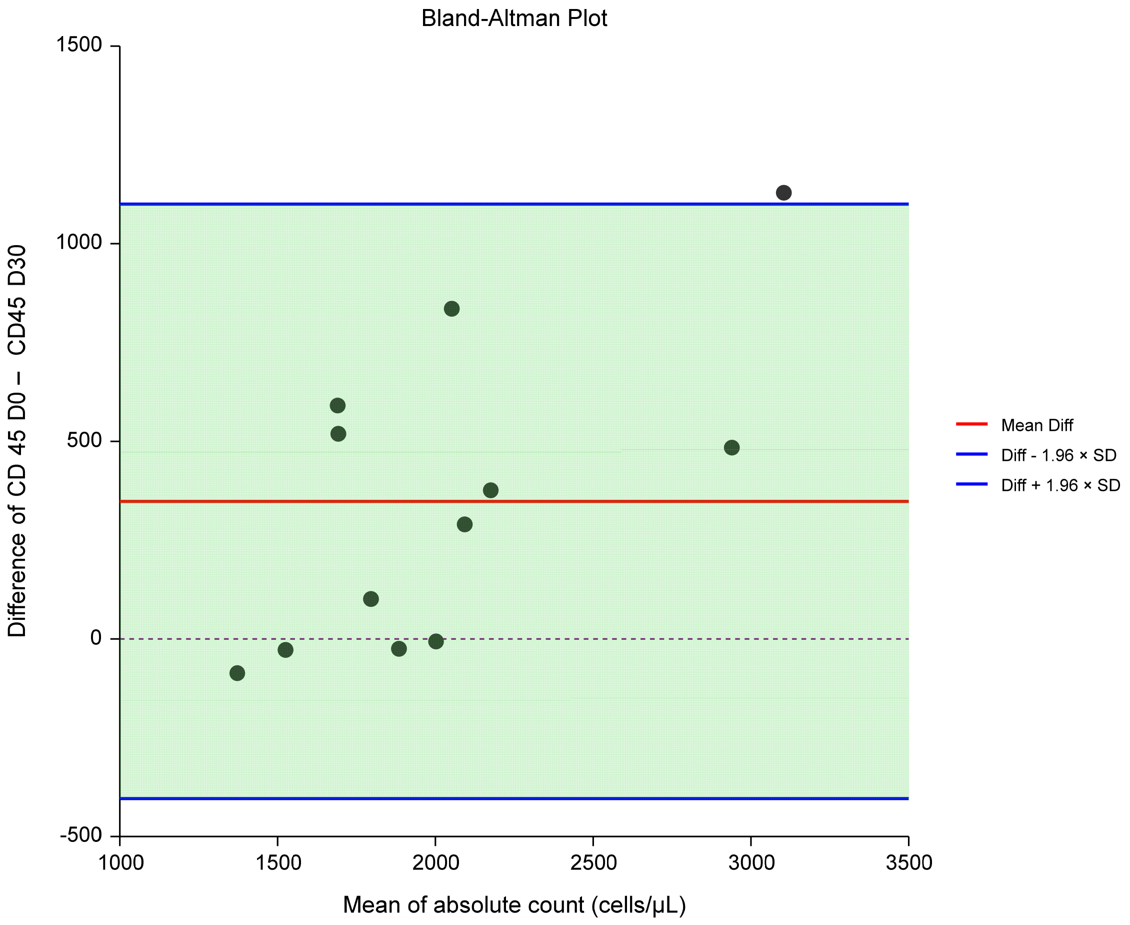

Figure S17. Bland-Altman plot of Absolute count of CD45 for Day 0 and Day 30 in placebo group.

Table S17. Bias and Limits of Agreement of Absolute count of CD45 for Day 0 and Day 30 in placebo group.

**Parameter**	**Count**	**Value**	**Standard Deviation**	**95.0% LCL of Value**	**95.0% UCL of Value**
Bias (Difference)	12	348.26	383.04	104.89	591.64
Lower Limit of Agreement	12	−402.50	194.54	−830.69	25.69
Upper Limit of Agreement	12	1099.03	194.54	670.84	1527.22

Limits of Agreement = Difference ± 1.96 × (Standard Deviation of Difference)

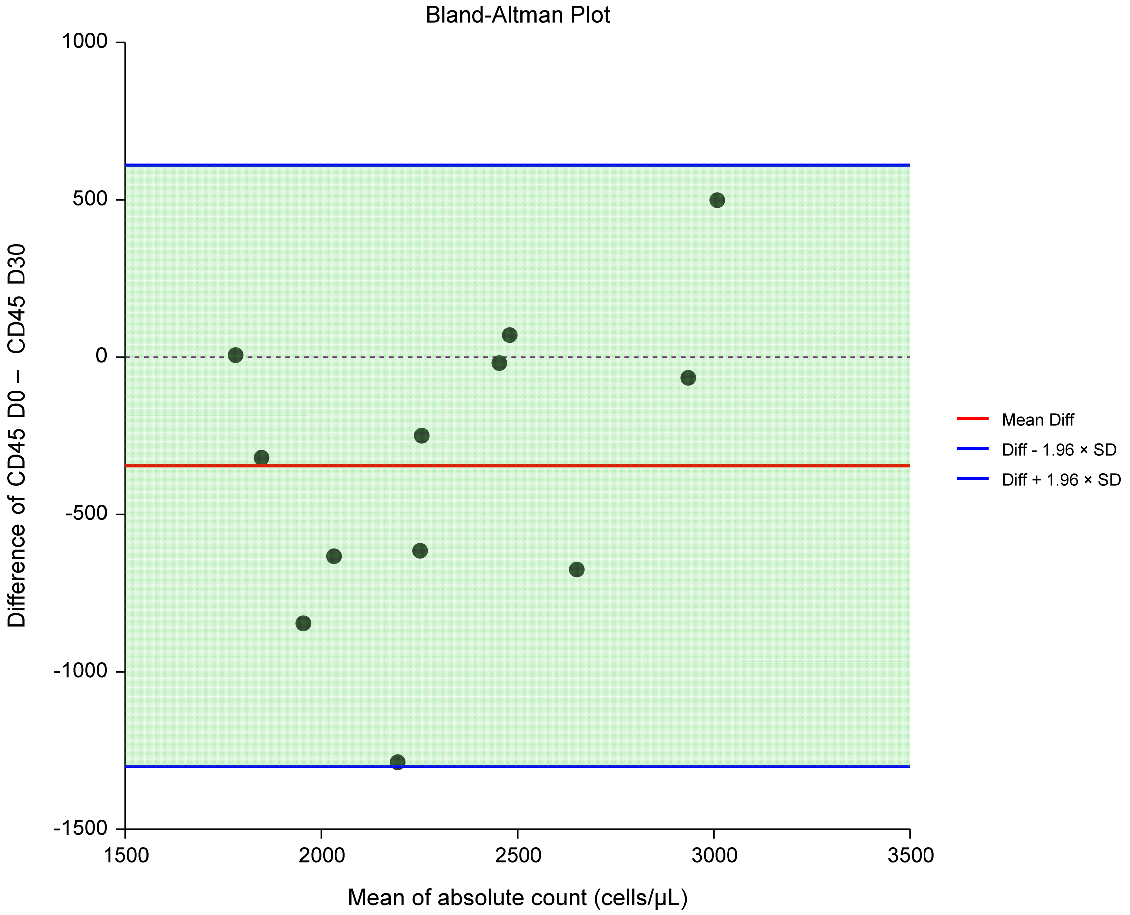

Figure S18. Bland-Altman plot of Absolute count of CD45 for Day 0 and Day 30 in test group.

Table S18. Bias and Limits of Agreement of Absolute count of CD45 for Day 0 and Day 30 in test group.

Parameter	Count	Value	Standard Deviation	95.0% LCL of Value	95.0% UCL of Value
Bias (Difference)	12	−344.39	486.68	−653.61	−35.16
Lower Limit of Agreement	12	−1298.28	247.18	−1842.31	−754.24
Upper Limit of Agreement	12	609.50	247.18	65.47	1153.54

Limits of Agreement = Difference ± 1.96 × (Standard Deviation of Difference)

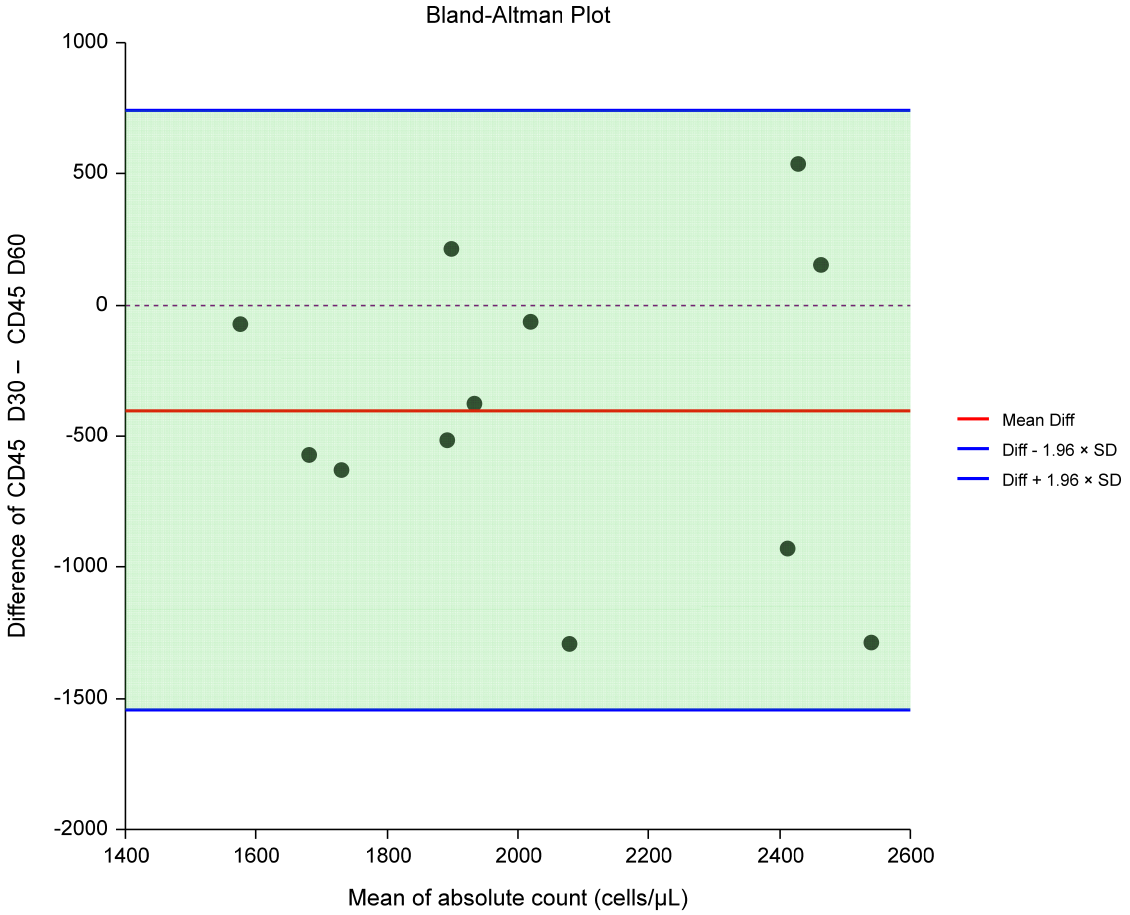

Figure S19. Bland–Altman plot of Absolute count of CD45 for Day 30 and Day 60 in placebo crossed over to test group.

Table S19. Bias and Limits of Agreement of Absolute count of CD45 for Day 30 and Day 60 in placebo crossed over to test group.

Parameter	Count	Value	Standard Deviation	95.0% LCL of Value	95.0% UCL of Value
Bias (Difference)	12	−402.92	583.11	−773.40	−32.43
Lower Limit of Agreement	12	−1545.80	296.15	−2197.63	−893.98
Upper Limit of Agreement	12	739.97	296.15	88.14	1391.80

Limits of Agreement = Difference ± 1.96 × (Standard Deviation of Difference)

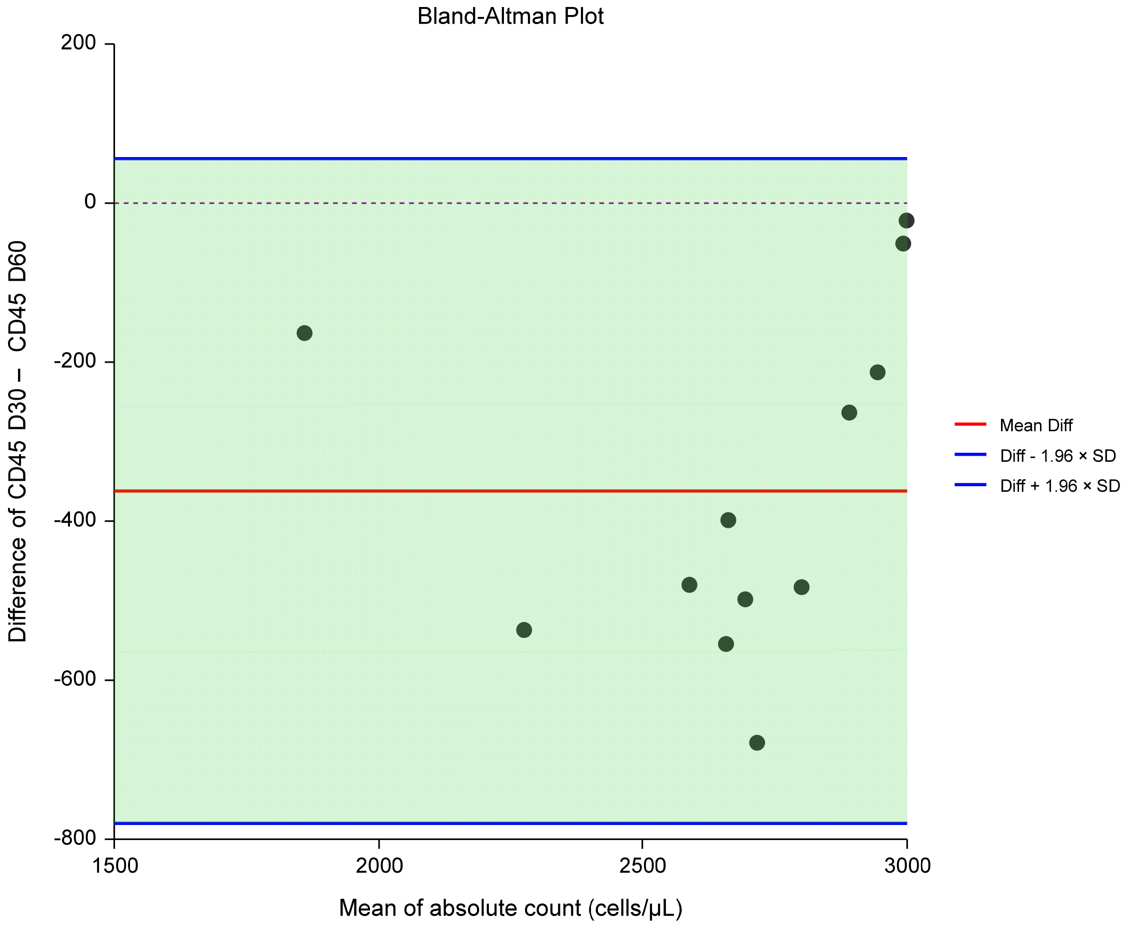

Figure S20. Bland-Altman plot of Absolute count of CD45 for Day 30 and Day 60 in test group.

Table S20. Bias and Limits of Agreement of Absolute count of CD45 for Day 30 and Day 60 in test group.

Parameter	Count	Value	Standard Deviation	95.0% LCL of Value	95.0% UCL of Value
Bias (Difference)	12	−361.88	213.23	−497.37	−226.40
Lower Limit of Agreement	12	−779.82	108.30	−1018.19	−541.46
Upper Limit of Agreement	12	56.05	108.30	−182.31	294.42

Limits of Agreement = Difference ± 1.96 × (Standard Deviation of Difference)

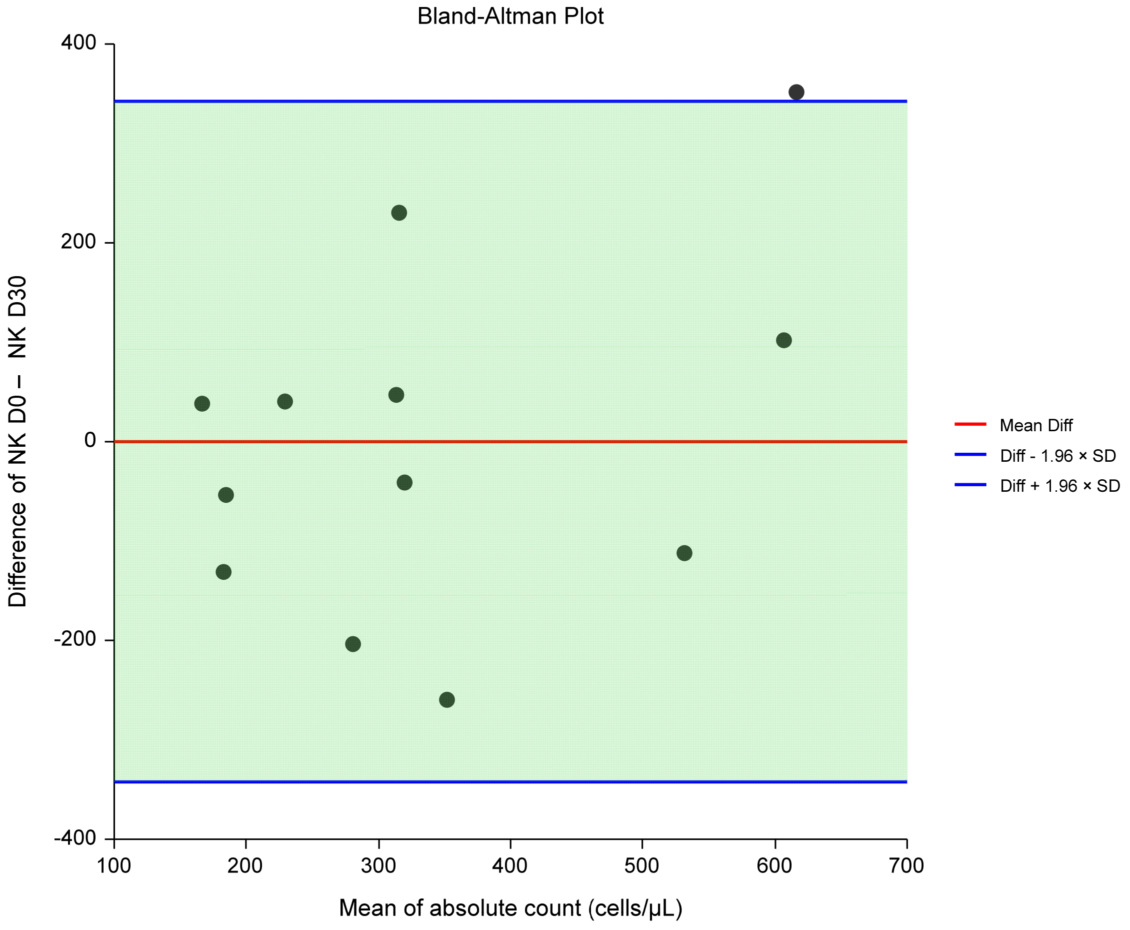

Figure S21. Bland-Altman plot of Absolute count of NK for Day 0 and Day 30 in placebo group.

Table S21. Bias and Limits of Agreement of Absolute count of NK for Day 0 and Day 30 in placebo group.

Parameter	Count	Value	Standard Deviation	95.0% LCL of Value	95.0% UCL of Value
Bias (Difference)	12	0.69	174.69	−110.31	111.68
Lower Limit of Agreement	12	−341.71	88.72	−536.99	−146.43
Upper Limit of Agreement	12	343.09	88.72	147.81	538.37

Limits of Agreement = Difference ± 1.96 × (Standard Deviation of Difference)

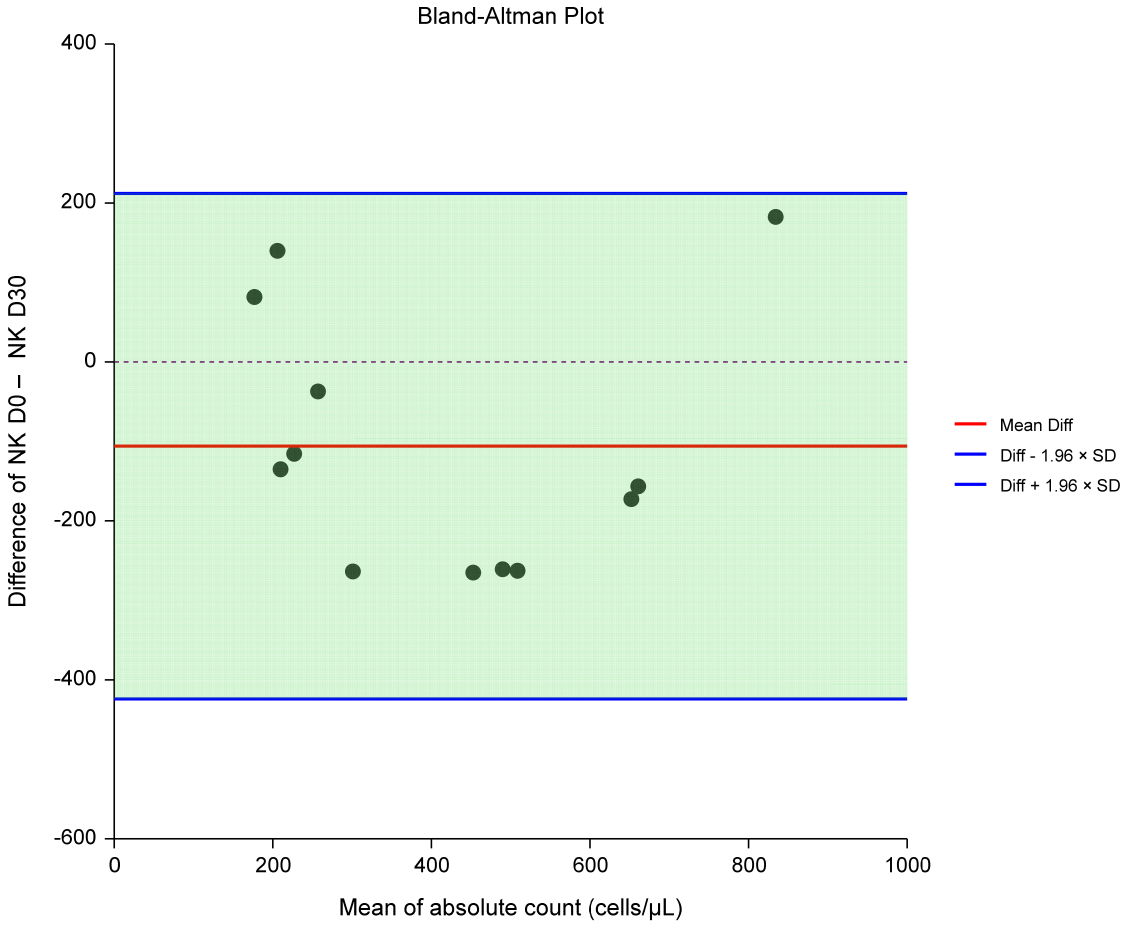

Figure S22. Bland-Altman plot of Absolute count of NK for Day 0 and Day 30 in test group.

Table S22. Bias and Limits of Agreement of Absolute count of NK for Day 0 and Day 30 in test group.

Parameter	Count	Value	Standard Deviation	95.0% LCL of Value	95.0% UCL of Value
Bias (Difference)	12	−105.45	162.45	−208.66	−2.23
Lower Limit of Agreement	12	−423.85	82.51	−605.45	−242.25
Upper Limit of Agreement	12	212.96	82.51	31.36	394.56

Limits of Agreement = Difference ± 1.96 × (Standard Deviation of Difference)

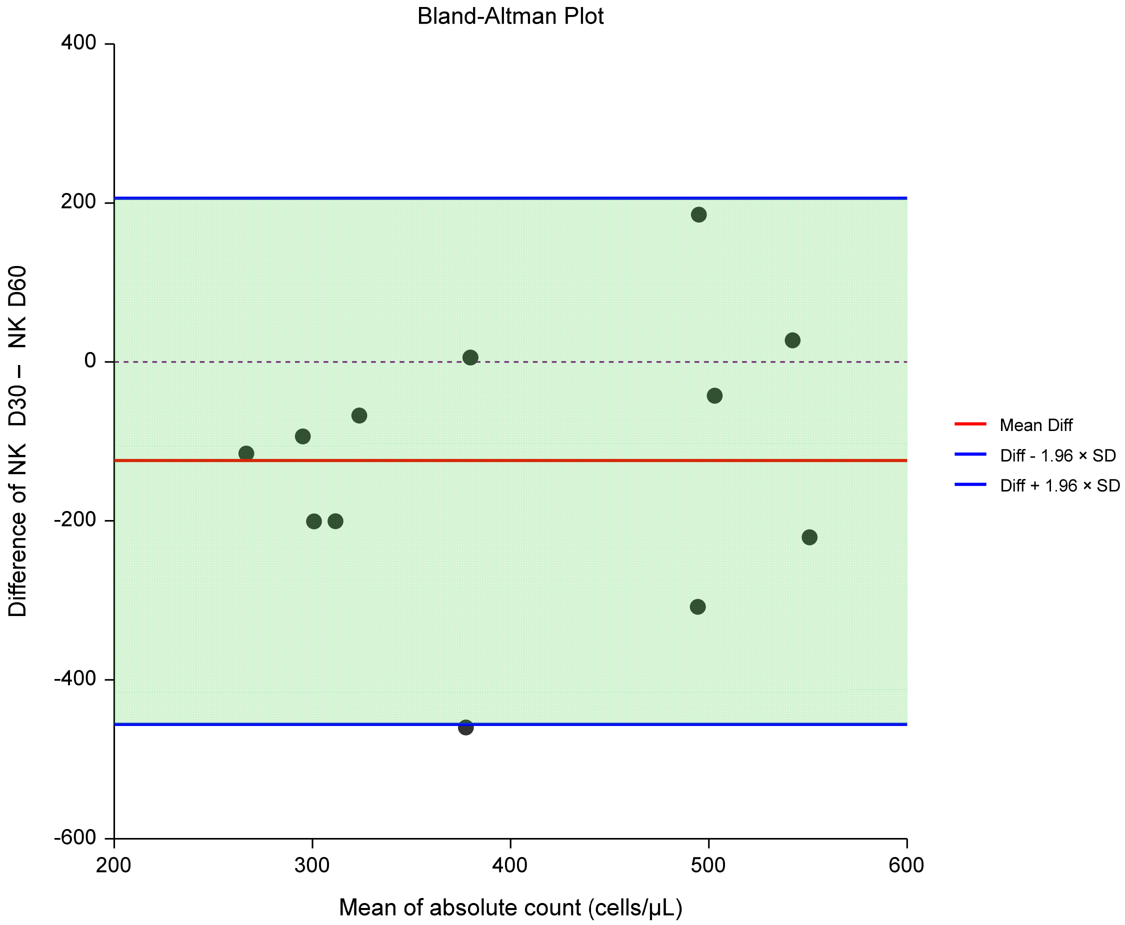

Figure S23. Bland-Altman plot of Absolute count of NK for Day 30 and Day 60 in placebo crossed over to test group.

Table S23. Bias and Limits of Agreement of Absolute count of NK for Day 30 and Day 60 in placebo crossed over to test group.

Parameter	Count	Value	Standard Deviation	95.0% LCL of Value	95.0% UCL of Value
Bias (Difference)	12	−124.22	168.97	−231.58	−16.86
Lower Limit of Agreement	12	−455.40	85.82	−644.28	−266.51
Upper Limit of Agreement	12	206.96	85.82	18.08	395.85

Limits of Agreement = Difference ± 1.96 × (Standard Deviation of Difference)

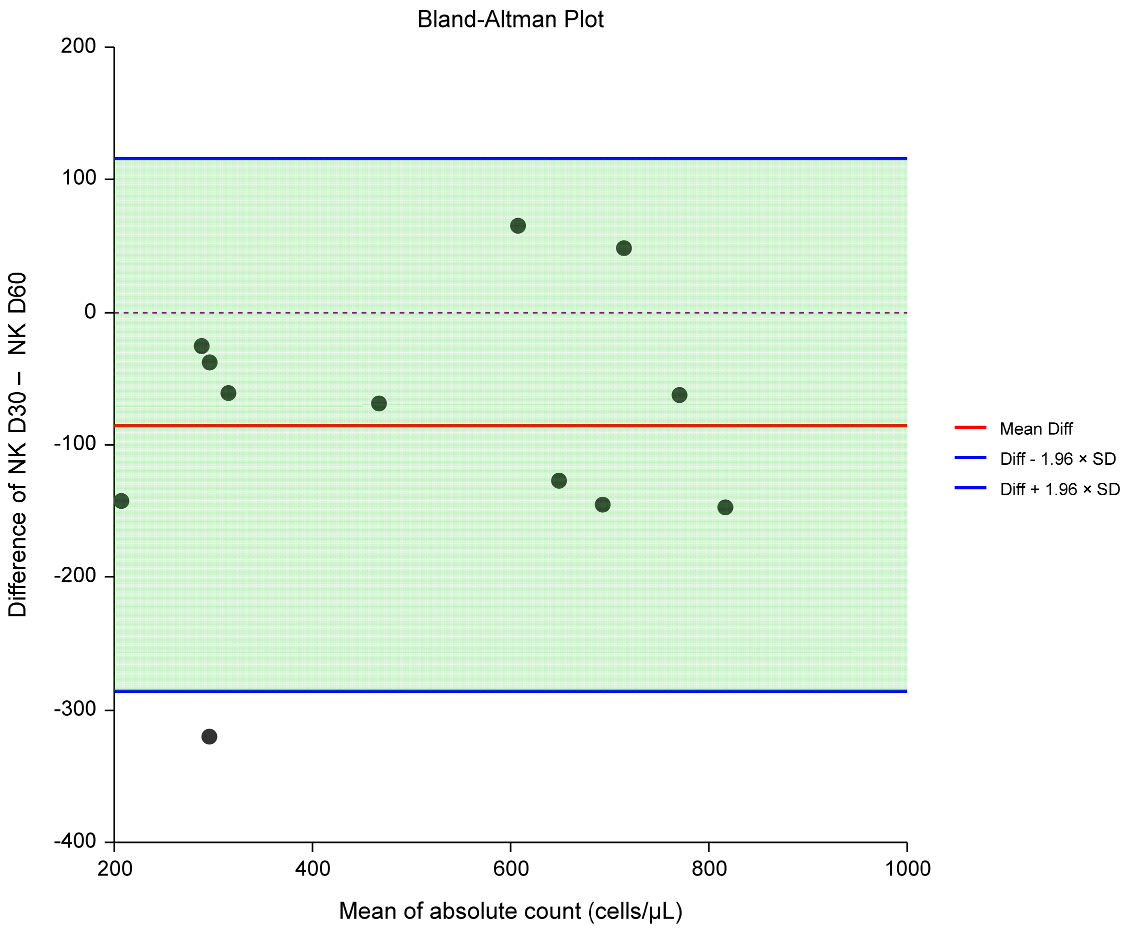

Figure S24. Bland-Altman plot of Absolute count of NK for Day 30 and Day 60 in test group.

Table S24. Bias and Limits of Agreement of Absolute count of NK for Day 30 and Day 60 in test group.

Parameter	Count	Value	Standard Deviation	95.0% LCL of Value	95.0% UCL of Value
Bias (Difference)	12	−85.32	102.41	−150.39	−20.25
Lower Limit of Agreement	12	−286.05	52.01	−400.53	−171.56
Upper Limit of Agreement	12	115.41	52.01	0.92	229.89

Limits of Agreement = Difference ± 1.96 × (Standard Deviation of Difference)
