# Supplementary material for: Immunomodulatory Effect of Withania somnifera (Ashwagandha) Extract—A Randomized, Double-Blind, Placebo Controlled Trial with an Open Label Extension on Healthy Participants
Source: J Clin Med. 2021 Aug 18;10(16):3644. doi: 10.3390/jcm10163644 (PMC8397213; doi:10.3390/jcm10163644)
Supplement: Supplementary file 1 [file jcm-10-03644-s001.zip › supplementary file 5 - final.pdf]

Supplementary file 5. Studies on Ashwagandha

S.N	Study	Parts used	Summary
[1]	Khan, S. et al. 2009 [9]	WS Leaf extract	• Enhanced Th1 cytokine IFN-γ expression in Con A primed splenocytes in vitro.<br>• Dose-dependent proliferation of T cells and improved their ability to secrete IL-2 and IFN-γ<br>• Prominent enhancement in proliferation and differentiation of lymphocytes.
[2]	Kaur T, 2017 [23]	WS Leaf extract	• The stress induced expression of inflammatory and immune response markers GFAP, TNFα, IL-6, OX-18 and OX-42 in VSD animals was found to be modulated by ASH-WEX.<br>• The stress induced apoptosis was suppressed in WSD group as indicated by expression of NF-κB, AP-1, Bcl-xL and Cytochrome c.
[3]	Kuttan, G. 1996 [11]	WS Plant extract	• Significantly increase the total WBC count in normal Balb/c mice<br>• Reduced the leucopenia induced by sublethal dose of gamma radiation.<br>• Increased the bone marrow cellularity significantly<br>• Normalised the ratio of normochromatic erythrocytes and polychromatic erythrocytes in mice after the radiation exposure.
[4]	Ziauddin, M. et al., 1996 [12]	WS Plant extract	• Prevented myelosuppression in mice treated with cyclophosphamide, azathioprin, or prednisolone.<br>• A significant increase in hemoglobin concentration, red blood cell count, white blood cell count, platelet count and body weight was observed in Ashwagandha-treated mice as compared with untreated (control) mice.<br>• Significant increases in hemolytic antibody responses towards human erythrocytes.
[5]	Davis & Kuttan 2002 [13]	WS Plant extract	• Withania somnifera could stimulate the generation of cytotoxic T lymphocyte and it may reduce tumour growth.
[6]	Malik et al. 2007 [14]	WS Root extract	• Stimulated cell mediated immunity, IgM and IgG.<br>• Prominent enhancement in proliferation and differentiation of lymphocytes.

			• Enhanced expression of T helper cells (Th)1 cytokines interferon (IFN)-γ and interleukin (IL)-2 while Th2 cytokine IL-4 observed a moderate decline.<br>• Strongly activated macrophage functions indicated by enhanced secretion of nitrite, IL-12 and TNF-α.<br>• Supports predominantly Th1 immunity with increase in macrophage functions.
[7]	Priyanka G 2020 [24]	WS Root extract	• Statistically significant ($p < 0.05$) increase in total erythrocyte count, total leucocyte count, hemoglobin content, lymphocyte percentage
[8]	Kumar J, 2019 [25]	WS Root extract	• Co-treatment with zinc oxide nanoparticles (WS + ZnO NPs), Withaferin A along with zinc oxide nanoparticles (WA + ZnO NPs)-enhance phagocytic activity by counteracting mechanism of ZnO NPs toxicity
[9]	Barua A, 2013 [26]	WS Root powder	• Ashwagandha supplementation decreased the incidence and progression of OVCA.<br>• Both the stromal and intratumoral NK cell population increased significantly in Ashwagandha supplementated hens.
[10]	Turrini E,2016 [27]	WS Root extract	• Results show that WS has a significant cytotoxic and cytostatic potential, and induces ICD.<br>• Its proapoptotic mechanism involves intracellular Ca(2+) accumulation and the generation of reactive oxygen species.
[11]	Maurya SP, 2019 [28]	WS Root extract	• An overall decline in the percentage of CD38 expressing CD8 + T lymphocytes was observed, though the statistical significance was varied with different categories of HIV patients.
[12]	Bani, S. et al. 2006 [10]	WS Root extract	• Roots aqueous extract at 100 mg/kg resulted significant selective up-regulation of Th1 response.<br>• Significant increase in CD4 and CD8 counts as compared to control and cyclopsorin A, with a faster recovery of CD4+ T cells in immunesuppressed animals.
